# A coding and non-coding transcriptomic perspective on the genomics of human metabolic disease

**DOI:** 10.1093/nar/gky570

**Published:** 2018-07-09

**Authors:** James A Timmons, Philip J Atherton, Ola Larsson, Sanjana Sood, Ilya O Blokhin, Robert J Brogan, Claude-Henry Volmar, Andrea R Josse, Cris Slentz, Claes Wahlestedt, Stuart M Phillips, Bethan E Phillips, Iain J Gallagher, William E Kraus

**Affiliations:** 1 Division of Genetics and Molecular Medicine, King's College London, London, UK; 2 Scion House, Stirling University Innovation Park, Stirling, UK; 3 School of Medicine, Derby Royal Hospital, Derby, UK; 4 Department of Oncology-Pathology, Science For Life Laboratory, Stockholm, Sweden; 5 Miller School of Medicine, University of Miami, USA; 6 McMaster University, Canada; 7 Duke University School of Medicine, Durham, USA; 8 School of Health Sciences and Sport, University of Stirling, Stirling, UK

## Abstract

Genome-wide association studies (GWAS), relying on hundreds of thousands of individuals, have revealed >200 genomic loci linked to metabolic disease (MD). Loss of insulin sensitivity (IS) is a key component of MD and we hypothesized that discovery of a robust IS transcriptome would help reveal the underlying genomic structure of MD. Using 1,012 human skeletal muscle samples, detailed physiology and a tissue-optimized approach for the quantification of coding (>18,000) and non-coding (>15,000) RNA (ncRNA), we identified 332 fasting IS-related genes (CORE-IS). Over 200 had a proven role in the biochemistry of insulin and/or metabolism or were located at GWAS MD loci. Over 50% of the CORE-IS genes responded to clinical treatment; 16 quantitatively tracking changes in IS across four independent studies (*P* = 0.0000053: negatively: *AGL, G0S2, KPNA2, PGM2, RND3* and *TSPAN9* and positively: *ALDH6A1, DHTKD1, ECHDC3, MCCC1, OARD1, PCYT2, PRRX1, SGCG, SLC43A1* and *SMIM8*). A network of ncRNA positively related to IS and interacted with RNA coding for viral response proteins (*P* < 1 × 10^−48^), while reduced amino acid catabolic gene expression occurred without a change in expression of oxidative-phosphorylation genes. We illustrate that combining in-depth physiological phenotyping with robust RNA profiling methods, identifies molecular networks which are highly consistent with the genetics and biochemistry of human metabolic disease.

## INTRODUCTION

Nucleic acid profiling technologies combined with systems biology and in-depth clinical phenotyping provide unparalleled opportunities to study mechanisms of human disease (1–6). Type 2 diabetes (T2DM), whose increased prevalence is proposed to be driven by obesity (7), is a complex metabolic disease involving gene-environment interactions reflective of excess nutrient intake, insufficient physical activity and, potentially, ageing. A feature of T2DM is a variable loss of peripheral insulin sensitivity (IS) (8,9); in humans predominantly reflecting reduced efficacy of insulin signaling (‘insulin resistance’, IR) in skeletal muscle and liver (10,11). GWAS analyses have revealed >200 genomic loci linked to phenotypes underpinning the pathophysiology of T2DM (12). In turn, these loci provide a link to thousands of *potential* ‘disease’ genes as, by design, GWAS projects do not identify the individual gene responsible for the genetic association (13–15). One recent T2DM GWAS analysis identified 128 markers across 113 genomic loci using 26,676 T2DM cases and 132,532 controls, with an additional ∼70,000 samples used for post-hoc validation (16). Follow-up informatics analysis suggested these loci harbor genes that predominantly impact on insulin secretion and adipocyte or hepatocyte biology. However, estimation of the causal risk genes is currently imprecise (13,14), constraining the contextualization of GWAS results, such that additional lines of evidence to help identify the most likely ‘disease gene’ at otherwise validated loci, is warranted (15,17). Recent efforts to apply expression quantitative trait loci (eQTL) analysis to the genomics of metabolic disease has yielded limited progress (18), and this probably reflects the reported assumptions limiting the sensitivity of eQTL analysis (19), as well as suboptimal tissue transcriptomics.

Direct quantification of the human coding and non-coding transcriptome in tissues physiologically responsible for regulating IS provides an alternative, and more quantitative route to associate individual genes with the genomics underpinning metabolic disease (20). Furthermore, characterizing the transcriptional response to treatments which improve IS, would support a causal role for individual genes, particularly if consistent with genetic association analysis (6). Historically, clinical RNA profiling studies have been small, and have relied on DNA microarrays biased towards the 3’ end of protein-coding transcripts (21–23), resulting in a lack of a consistent global molecular narrative for IS in skeletal muscle. This lack of clarity reflects technical caveats, such as concurrent drug-treatment (for T2DM patients), inadequate consideration of clinical phenotype (23,24), as well as incomplete capture of the transcriptome (25) – and limited statistical power (26). More modern transcriptomic techniques, such as short-read RNA sequencing (RNA-seq) and full transcript ‘tiling-type’ microarrays (27) attempt to provide a complete and accurate view of the transcriptome. While potentially more-suited for studying cells in culture (28), RNA-seq analysis of muscle tissue provides a skewed measure of the protein-coding transcriptome and reproducibility in clinical studies remains surprisingly low, e.g. *R*^2^ < 0.7 (29,30) – too low for building reproducible diagnostics. The reason for this, in part, is that each tissue has a unique and skewed transcriptome, such that the resulting RNA-seq DNA library has an over- and -variable representation from – in the case of muscle – contractile and mitochondrial genes (29–32). This characteristic of the DNA library can also invalidate subsequent pathway analysis (33). In addition, RNA-seq profiles of individual tissues (25,30,34,35) do not include the majority of long non-coding RNAs (lncRNAs) and thus to date at least 50% of the tissue transcriptome remains unexplored in most clinical cohorts.

In the present study, we combined the benefits of signal-quantification from newer generation full transcriptome microarrays (dedicated non-competitive sequence-specific probes), with a novel tissue-specific transcriptome assembly approach, to over-come limitations of conventional (36) array analysis, while providing far more extensive analysis of lncRNAs than RNA-seq (32,37). In combination with detailed phenotyping, and multiple clinical intervention studies, we identified causality-linked and dynamic components of the metabolic disease transcriptome, providing an independent global genomic perspective, in support of many GWAS-MD loci, in a very resource efficient manner.

## MATERIALS AND METHODS

**Figure 1.**
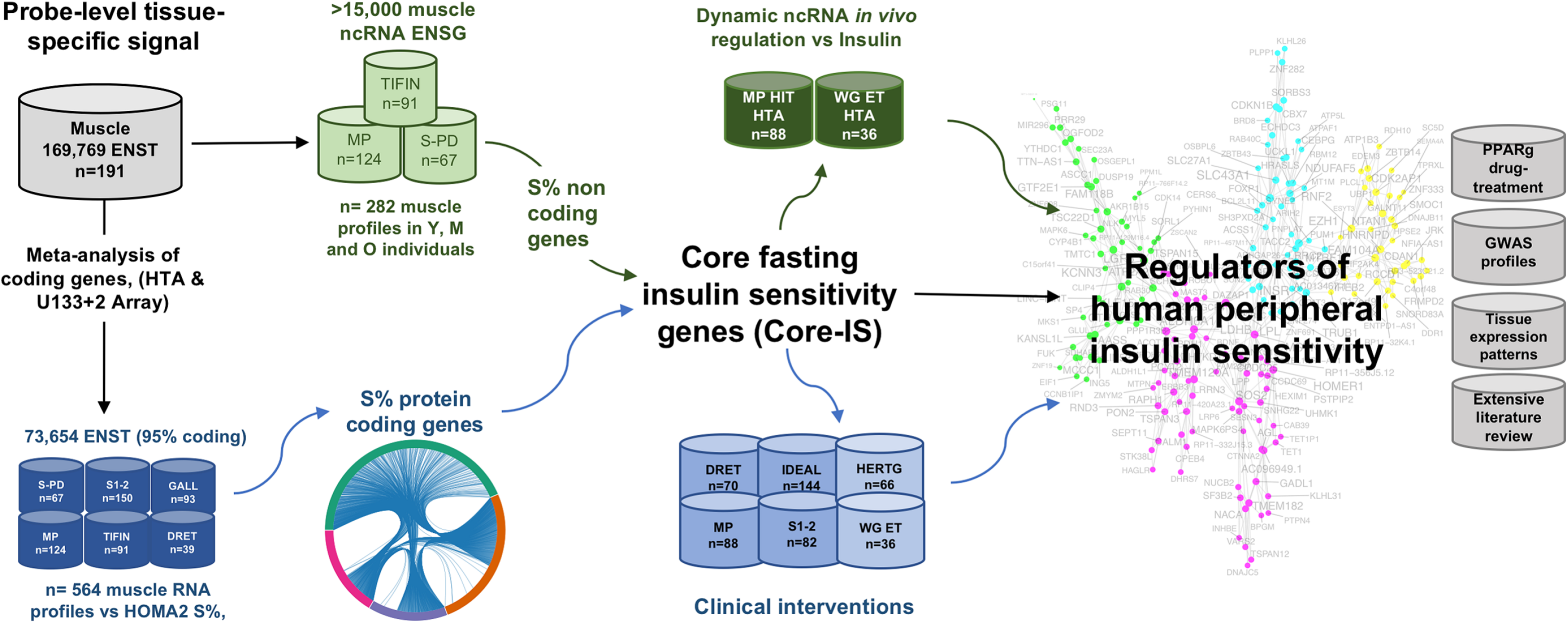
**Analysis process**. Human muscle biopsy RNA profiles came from clinical projects named STRRIDE I, II (S1–2) and III (S-PD), Meta-Predict (MP), TIFIN, DRET, Gallagher *et al*. (GALL), Weigert *et al*. (WG), Sears *et al*. (SEARS), Heritage (HERTG) and IDEAL (2,22,37,38,46,50–54,,56). The present project involved the production and analysis of 470 new samples profiled on the HTA 2.0 Affymetrix platform along with 144 new samples profiled on the Illumina 12 platform; and 466 of our previously published (22,37,53,54) U133+2 muscle RNA profiles and literature cohorts (where appropriate clinical data was available). The HTA 2.0 platform was processed with a 169 796 probe-set CDF (ENST), representing ∼18 000 protein-coding genes and ∼16 000 non-coding genes. The older array platform offered an opportunity to replicate protein-coding gene expression with 73 654 probe-sets (ENST) being common to the HTA 2.0 platform. All samples and clinical data underwent a rigorous quality control check to ensure that the microarray profile was technically robust (minimal variation in NUSE score), and the insulin and glucose values were within the operating range of the HOMA2-IR model (10). Less than 10% of any project samples were lost to lack of one of these two components. The HOMA2-IR model requires either ELISA (more specific insulin determination) or RIA (less specific) in p.mol.l and glucose in mmol.l and incorporates non-linear features of the glucose-insulin relationship. The HOMA2-IR is a *bona fide* physiological model for peripheral insulin resistance correlating with other more precise but less physiological models (10). We utilized the log_10_ transformed insulin sensitivity values (S%) as the input values for the linear covariate adjusted modeling of peripheral insulin sensitivity. A meta-analysis of the individual covariate adjusted *P*-values was aggregated using the Stouffer method and an FDR calculated (60). This process identified ∼1000 genes related to S%, of which >300 were consistently (directionality) regulated; yielding a ‘core fasting insulin sensitivity gene list’ (**CORE-IS**). Having identified this reproducible set of S% genes, the causal nature of these genes during dynamic regulation of S% was investigated using clinical intervention studies (22,37,53,54) and systems biology methodologies (data-driven co-expression analysis (77,136); protein–protein interaction databases (137), transcription factor binding-site analysis (63) and ‘guilt-by-association’ methodologies (138)).

All new arrays has been deposited at GEO (GSE104235). The remainder of our existing array data and data used from other labs are also available at GEO (GSE59880, GSE47881, GSE47969, GSE48278, GSE72462, GSE73142, GSE83619 and GSE13070). The transcriptome was defined by the latest generation gene-chip pipeline and then utilized to select the valid transcriptome from earlier generation technologies (Figure 1).

### Customizing RNA expression quantification to individual tissue types using a custom CDF

We hypothesized that processing of raw data from the Affymetrix HTA 2.0 array (37) would benefit greatly from optimization, to more efficiently detect gene expression, as currently no account is made of study specific probe performance. The HTA 2.0 gene-chip contains 6.9 million short ‘probes’ which are computationally combined into groups (probe-sets) representing individual RNA transcripts (or part of a transcript) using a ‘map’ named the chip definition file (CDF). The CDF combines probe signals irrespective of whether each probe represents an active ‘signal’ in the tissue or cell type being profiled or not (27,36,38,39), i.e. there is no probe-level filtering. This in turn suggests that the present signal ‘summarization’ process is inefficient as it will include poor performing probes and unnecessarily incorporate back-ground noise. More accurate determination of transcript expression, coupled with studies incorporating larger sample sizes should allow for the establishment of the first robust IS RNA signature in humans.

The first step in our approach was to check the sequence specificity of the 6.9 million probes on the HTA 2.0 array (39). The physical design of each probe is fixed and incorporates the features of the reference genome used at the time of design and thus each probe must be discarded if it is no longer specific. We mapped probe sequences to the latest reference genome (GRCh38_82p3) using bowtie (40) and probes which did not map to a single genomic location were discarded. Secondly, using the aroma.affymetrix package (41) we assessed the signal characteristics for each *probe* and those probes with a low and variable signal (e.g. <10 units and co-efficient of variation >25% (for muscle)) were removed. This process was applied to each tissue type investigated. The ∼50,000 probes that have an extreme GC content (i.e. <20%, >80%) were also removed, because the GC adjustment model is not linear at these extreme values. The remaining probes were combined into ‘probe-sets’, with three probes or more, and production of this ‘experiment specific’ CDF resulted in the removal of ∼3 million probes, i.e. >40% of the original chip. Notably, this filtering still allows for >200,000 transcripts (across the tissue types examined in this project). In the present analysis, each *probe-set* was based on ENSEMBL ENST definitions, and R Bioconductor packages (42) were used to update, assemble and summarize the expression data. We also included an exon specific probe-set, based on a recent observation that the *RYR1* gene has one additional variant, which lacks a short 15 bp exon (ENSE00002436759), that correlated with a structural feature of muscle (‘fiber' type) (43).

The HTA 2.0 chip processed with this new muscle CDF yielded 169,769 ENST probe-sets (This CDF is provided at GEO). Notably, the discarded probes (e.g. signal <10 units in this example) *would* have been otherwise incorporated into probe-sets that had an average signal of ∼70 units, i.e. ∼7 times greater than the poor performing probes (Supplemental Figure S1A). Use of a ‘tissue-specific’ CDF map for quantification of the transcriptome should reduce the influence of back-ground noise during normalization (44). Indeed, use of this muscle CDF improved detection of differentially expressed ‘exercise’ genes ∼3–5-fold above a fixed FDR value, and demonstrated a high technical reproducibility for quantifying IS related genes (Pearson coefficient of determination (*R*^2^) of 0.96, Supplemental Figure S1B). The same tissue-specific approach was used to produce CDFs for adipose tissue and islet cell datasets (37,45) and to process previously published HTA 2.0 muscle data. Quality control was performed using normalized unscaled standard error (NUSE) and principal components analysis (PCA).

For the non HTA chip muscle data, quality control was performed as above and <10% of previously published samples were removed (or lacked valid insulin data, see below). Data generated on the U133+2 platform (predominately protein-coding RNA expression) was updated with a CDF that mapped probes to 73,654 probe-sets (ENST) in common with the muscle-specific HTA 2.0 profile. Our newly generated Illumina Human HT-12 V4.0 array data were processed using quantile normalization, as previously described (46). Gene names were used to map results to those ENST probe-sets on the HTA 2.0/U133+2 platforms (with the highest signal being chosen when a gene was represented more than once) yielding 47,216 transcripts (equating to 31 326 genes). The new Illumina array data was produced from the ‘IDEAL’ cohort (46) and was used, along with three literature data-sets, for independent validation of the RNA responses to clinical treatment (Figure 1). As expected the concordance of the Illumina platform results, was less than observed between probe-set based specific tissue confirmed ENSTs.

### Clinical samples, insulin analysis and RNA production

The present project relied on 1,012 human muscle biopsy samples. We produced RNA from 470 human skeletal muscle biopsy samples from our recent clinical studies for profiling on the HTA 2.0 gene-chip (Metapredict (MP), STRRIDE PD (S-PF) and TIFIN, Figure 1) (47–52). Additionally, we re-processed and updated 466 of our previously analyzed muscle gene-chip profiles (STRRIDE I and II (S1–2), Gallagher (GALL), IDEAL, HERITAGE (HERTG) and Derby Resistance Training Study (DRET), Figure 1) (22,37,53–55). A further 176 gene-chip profiles were identified from the literature (Weigert *et al*. (WG) and *in vivo* drug treatment (SEARS) studies in Figure 1) (38,45,56). Further, a subset of 568 muscle gene-chip profiles were paired, from 284 individuals before and after distinct clinical interventions aimed at improving IS (MP, S1–2, S-PD, DRET, IDEAL, HERTG, SEARS and WG, Figure 1) (38,46–48,50,52,53,56–58). Sixty percent of these samples were not included in the baseline fasting CORE-IS analysis (see below), and thus represent entirely independent samples from those used to discover the CORE-IS genes. The clinical methodologies have been extensively presented elsewhere (22,37,38,45–48,50–54,56–58). The demographics for the subjects contributing to the present analyses are presented in Table 1 (i.e. baseline data for IS modeling) and Supplemental Table S1 (clinical intervention cohorts for clinical response modeling), and Supplemental Table S3 (for the post-hoc gender comparison). Studies complied, as *per* the original publications, with the 2008 Declaration of Helsinki and were approved by the relevant ethics committees stated in each published clinical article, while all participants gave their written, informed consent to participate.

**Table 1.** Demographics of the clinical cohorts used for establishing the fasting peripheral insulin sensitivity transcriptome (2,22,37,46,50,51,53,54). The HTA 2.0 gene-chip platform data represent entirely new data while the U133+2 gene-chip data reflects our prior data, updated and re-processed. Values are median (range) or adjusted *R*^2^ using lm in *R. V*O_2_ peak is a measure of peak oxygen consumption or ‘maximal’ aerobic capacity, and the units are ml min^−1^kg^−1^, fasting glucose is m mol l^−1^, fasting insulin units are pmol l^−1^, while BMI and HOMA2-IR are unit-less and IS is percentile insulin sensitivity calculated from the HOMA2-IR model (10). *V*O_2_ peak is a trait that is highly inherited but also influenced by behavior (intensity and volume of physical activity)

	S-PD+MP	LVL-OLD	GALLAG	DRET	S1–2
Sample size (*n*)	191	91	93	39	150
Platform	HTA 2.0	HTA 2.0	U133+2	U133+2	U133+2
Gender (M/F)	89/103	50/41	64/29	22/17	75/84
AGE	43 (18–75)	71 (65–95)	56 (23–77)	49 (20–75)	50 (24–68)
BMI	30.5 (24.6–45.5)	26.6 (19.6–38.5)	29.6 (16.7–50.2)	25.9 (19.4–32.5)	30.1 (25.0–37.6)
VO_2_ peak	26.8 (13.2–46.9)	-	26.0 (10.6–55.8)	-	26.8 (13.2–43.9)
Fasting Glucose	4.8 (3.99–7.4)	5.5 (4.4–6.7)	5.9 (4.3–23.3)	5.6 (3.4–7.5)	5.3 (3.8–7.3)
Fasting insulin (ELISA)	57.5 (8.9–257.0)	-	63.1 (10.0–251.1)	23.4 (10.5–67.6)	-
Fasting Insulin (RIA)		110.8 (34.8–324.4)			54.6 (7.2–241.9)
HOMA2-IR	1.23 (0.31–9.33)	2.042 (0.67–5.96)	1.32 (0.31–21.88)	0.52 (0.23–1.63)	1.02 (0.13–4.47)
IS	81.7 (17.5–324.3)	48.9 (16.8–151.2)	75.9 (4.6–323.6)	193.1 (61.2–442.7)	98.15 (22.2–751.3)
*R* ^2^ IS versus age	<0.01	<0.01	0.01	0.03	<0.01
*R* ^2^ IS versus BMI	0.11	0.22	0.22	0.05	0.15
*R* ^2^ IS versus *V*O_2_ peak	0.02	-	0.12	-	<0.01

To produce the new HTA 2.0 array profiles, RNA was isolated using TRizol^®^ (Life Technologies) and dissolved in 20 μl RNAse-free water, processed to single-stranded sense fragmented DNA using the GeneChip^®^ WT PLUS Reagent Kit, which relies on a reverse transcription priming strategy that primes both poly-A and non-poly-A RNA. HTA 2.0 gene-chips were processed according to the manufacturer's protocol. Fragmented (5 μg) end-labeled sense strand target cDNA was hybridized to each array and scanned using a Gene Chip Scanner 30007G (Affymetrix Core, MPI A/S, Denmark). From the RNA processed, <5% of samples were excluded as outliers by visually evaluating NUSE plots prior to down-stream analysis. The technical reproducibility for detecting regulated genes was assessed by running RNA samples from the same subject/time-point several months apart and examining the correlation coefficient, without adjustment for any potential batch effects (Supplemental Figure S1B). For the Illumina Human HT-12 V4.0 arrays (Nestle Research Center, Lausanne, Switzerland) RNA was isolated from the skeletal muscle tissue by using TRizol^®^ (Life Technologies), purified using the Qiagen RNeasy Micro kit (Qiagen, Venlo, NL, USA) and RNA quality was checked using an Agilent 2100 bioanalyzer (Agilent Technologies, Amsterdam, NL).

There are numerous assays for insulin, each with their own performance characteristics (59). For one-hundred and ninety-one subjects (MP+S-PD studies) plasma insulin values (high-sensitivity, enzyme‐linked immunosorbent assay (ELISA) analysis, K6219 Dako, Sweden AB) were generated in a single core-lab. The Gallagher *et al*. study (22) also utilized a high-sensitivity ELISA (Dako Sweden AB), while the DRET study used a high-sensitivity ELISA from DRG Instruments (GmbH, Germany). The TIFIN study used the less sensitive radioimmunoassay (RIA, LINCO/Millipore), as did STRRIDE I/II (Access Immunoassay System, Beckman). IDEAL used a RIA (Architect System, Abbott Laboratories). Glucose values were determined with an oxidation reaction (YSI-2300 Stat Plus, Yellow Springs Instrument, Yellow Springs, OH). No individual insulin values were available from the published studies of Sears or Weigert *et al*. (38,56). All samples/subjects from our laboratories underwent updated quality control to ensure that the array profile was technically robust and that the insulin and glucose values were within the operating range for the HOMA2-IR model (10).

For the cell-culture RNA studies, ∼20 000 HepG2 cells (hepatocellular carcinoma cell line) cultured in OPTIMEM (Gibco) were transfected (96-well plates) with 20 nM of ASO (Integrated DNA Technologies; Supplemental data S1) using Lipofectamine 2000 (Thermo Fisher Scientific) for either 24, 48 or 72 h. RNA isolation and DNase treatment were performed by RNeasy Mini Kit (Qiagen, USA), in accordance to manufacturer's instructions. RNA quality and concentrations were determined by Nanodrop 2000 (Thermo Fisher Scientific, USA). Twenty nanograms of RNA were reversely transcribed using the High Capacity cDNA Reverse Transcription Kit (Thermo Fisher Scientific, USA), according to manufacturer's recommendations. qRT-PCR (primers, Supplemental data S1) was performed on a QuantStudio 6 Flex machine (Life Technologies) using probe-based assays (Integrated DNA Technologies, USA). Data was scaled to GUSB and PPIB and were expressed relative to cells treated with a commercial control ASO.

### Primary statistical analysis of gene expression

A table of normalized, log_2_ transformed RNA expression values was produced for each of the studies described above. First, we assessed the linear relationship between gene-expression and IS (log_10_ of the HOMA2 model S% values, (10)) together with age or BMI (Table 1), using analysis of variance (ANOVA) in R (i.e. using the following linear models in R: expression ∼ IS*age and expression ∼ IS*BMI). A correlation coefficient (*R*) and *P* value were calculated for each probe-set in the two bivariate analyses per study. Second, to produce a ‘significant’ sub-set of IS genes, we carried out a meta-analysis of each bivariate analysis across six clinical cohorts (*n* = 564, Figure 1), including the 73 654 ENST probe-sets common to all chip-types (99% of which were protein-coding), generating a false discovery rate (FDR) for each bivariate model separately, using the Stouffer method, in the R-package MetaDE (60), from the ANOVA *P*-values.

Third, any probe-set with an FDR <10% was filtered by univariate regression coefficient such that only those with a directionally consistent *R* value across the four largest studies was retained. This resulted in the set of ‘CORE-IS’ genes with a much lower average FDR. Post hoc analysis and interpretation of these findings considered gene-expression versus IS, when a covariate, e.g. BMI, was set as the primary variable in the ANOVA model (i.e. using the following linear models in R: expression ∼ BMI*IS). Further interpretation of the CORE-IS genes was based on gender dependent expression using a group of males and females (*n* = 89 per group) where HOMA2_IR, age and peak VO_2_ were balanced (Supplemental Table S3). The limma packge in R (eBayes and lmFIT functions) was used to determine DE with *P*-values adjusted using the fdr method (61).

For the ncRNA analysis there were a total of 282 HTA 2.0 profiles available. The analysis was performed as described above (i.e. using the two bivariate models, followed by filtering) for the combined S-PD and MP HTA 2.0. based studies (Figure 1, SMP, *n* = 191). Similar to the protein coding genes above, only ncRNAs with an FDR less than 10% (Benjamini-Hochberg FDR using the mult-test R package) for IS in both models and with a consistent (direction) *R* value in two studies; S-PD (*n* = 67) and MP (*n* = 124) were considered (93% were consistent). A third cohort consisting of older-subjects (65–95 years) was profiled on the HTA 2.0 array (TIFIN, *n* = 91, Figure 1) was used to further explore identified ncRNAs by comparing coefficients from univariate models. 52% of the IS relationships being consistent between the younger cohorts (S-PD and MP) and this older cohort. It was expected that there would be some differences due to both the impact of population stratification and potential interactions between IS and muscle age (62). The identified 86 ncRNAs completed the 332 CORE-IS gene list that was taken forward for validation and modeling.

Individual CORE-IS genes that causally regulate IS, should respond to treatments that prevent T2DM. We utilized both differential expression (DE) analysis using limma (61) and Spearman rank correlation on data from six distinct life-style intervention studies (three independent of the groups above, IDEAL, HERTG and WG) and separately, one diabetes drug treatment study (SEARS). For DE, *P*-values were subjected to meta-analysis and a Stouffer FDR was calculated as above (60) to establish which genes were consistently regulated across each (very distinct) life-style intervention study. For each of four studies with valid individual insulin measures (MP, S1–2, IDEAL and DRET, Figure 1), log_2_ fold changes were calculated for the 332 CORE-IS genes and their correlation (Spearman) with changes in IS, per subject was calculated. DE was also calculated in a seventh drug-treatment study (56), where paired t-tests were used to examine if the CORE-IS genes were responsive to 12 weeks of treatment with a PPARγ agonist (The authors (56) would not provide individual clinical values). Transcription factor binding site analysis was carried out using SSA in oPOSSUM (63), with the 24,752 genes in the database as background (JASPAR CORE profiles (64)). Conservation cut-off was set at 0.6 and a matrix score threshold of 85%, searching 10K up- and down-stream of each CORE-IS gene transcription start site. Results were interpreted in light of the outcome found by analyzing the entire CORE-IS genes and the treatment modulated DE IS list, such that enrichment over these larger lists was considered interesting and results plotted (using Gviz, biomaRt and GenomicRanges packages). Heat-maps were generated using Morpheus (https://software.broadinstitute.org/morpheus/).

### Validation using published literature and GWAS analysis

To compare the CORE-IS genes identified in the present study with GWAS-MD loci identified during the past decade, we utilized three main resources from the GWAS research community; the Type 2 Diabetes knowledge portal (www.type2diabetesgenetics.org/home/portalHome) which includes many phenotypes beyond T2DM, and the NHGRI-EBI Catalog (www.ebi.ac.uk/gwas/home), together with gene lists presented in the most recent GWAS-MD publications (7,16,65–76), i.e. those that recently reported genetic associations for risk of development of T2DM, insulin action (IS [insulin or HOMA_IR], fasting glucose, fasting lipids [HDL, LDL and triglycerides] and body composition [BMI or adiposity]). The NHGRI-EBI Catalog and published gene lists were included at the 5 × 10^−8^ GWAS significant threshold. The Type 2 Diabetes knowledge portal provides three demarcations for significance; ‘GWAS-level’ 5 × 10^−8^, ‘suggestive association’, 5 × 10^−4^ and ‘red’ indicating that the loci was non-significant for any GWAS phenotype. A systematic literature review, whereby the CORE-IS gene name was searched with and without the terms; ‘insulin’, ‘diabetes’ or ‘obesity’ (Pubmed, June 2017) followed by an extensive assessment of whether a direct biochemical interaction had been reported, was recorded (see below and Supplementary files).

### Gene-network structure and pathway analysis

As we produced *continuous* quantitative data, for gene expression, from significant GWAS loci, we could utilize probabilistic network analysis incorporating inferred causal relationships between molecules and disease (4,5) to explore the biology of IS. The R-package MEGENA (77) was used to identify network structures within the largest and most detailed sub-group (SMP, *n* = 191, HTA 2.0 data and single-core high-sensitivity insulin assay). Our aim was to characterize the interacting components of the CORE-IS gene-list (78), determine if they formed significant sub-networks using strict thresholds (FDR < 1% for Spearman correlation; *P* < 0.01 for module significance and *P* < 0.01 for network connectivity; and 1000 permutations for calculating FDR and connectivity *P*-values). Stability of individual sub-clusters of a network was assessed based on the impact of a data-split on compactness on a parent cluster, versus the impact of randomly permuting or inserting nodes. Network plots were produced using Fruchterman–Reingold force-directed plotting (77) within MEGENA. Each presented significant network was populated with visual identifiers (color-coded circles) for GWAS-MD loci.

Interpretation of each significant network or module was carried out in several ways. Firstly, using NetworkAnalyst (http://www.networkanalyst.ca/) and the IMEx Interactome curated protein–protein interaction database (79). Each gene list was analyzed using default settings and minimum order network was chosen for gene ontology analysis (GO, Biological Process, GOBP). Enriched GOBP, with FDR < 1 × 10^−5^, were considered of interest. For each analysis, the result was confirmed after removing Ubiquitin because we noted it appeared as the highest ranked gene (‘betweeness’ score) in every protein interactome, regardless of the input gene ID list (probably reflecting technology bias, (33)). To further describe each network module, summary statistics were calculated for the proportion of ncRNAs, number of positively associating IS genes, the sub-phenotypes underlying the link with the GWAS-MD loci, and the number of genes responsive to interventions. Finally, for significant modules (e.g. treatment responsive genes) TF analysis (as above) and guilt-by-association (GBA) analysis, was performed (80). For the latter, the ‘Genefriends’ human transcriptome database (81) was used to help locate additional links between CORE-IS genes, pathways and metabolic disease GWAS loci.

## RESULTS

A total of 326 *pre*-intervention new muscle samples were profiled on the HTA 2.0 gene-chip (MP, S-PD and TIFIN, Figure 1). We updated the processing of 424 of our previously published muscle gene-chip profiles (Affymetrix U133+2 gene-chip [S1–2, GALL, DRET and HERTG, Figure 1 (2,22,37,46,50,51,53,54)] and several literature-based cohorts (38,53,56).

### Identification of a robust set of genes which covary with fasting peripheral insulin sensitivity

To produce a high-stringency set of IS related *protein* coding genes, we performed meta-analysis of 564 samples originating from these six independent cohorts (73,654 ENST, on HTA 2.0 arrays (two studies) and U133+2 arrays (four studies), Figure 1). Each study had a comparable distribution of IS (Supplemental Figure S2) and clinical characteristics (Table 1). The *P*-values derived from the relationship between RNA abundance and IS in each of six studies were subjected to meta-analysis using the Stouffer method (60) identifying 1005 protein coding genes (mean FDR = 1.7%). To be considered further, the R value for RNA expression versus fasting IS had to have a consistent directionality across the four largest cohorts. This led to the identification of 246 protein-coding genes, forming part of the CORE-IS gene list (Supplemental data S2).

**Figure 2.**
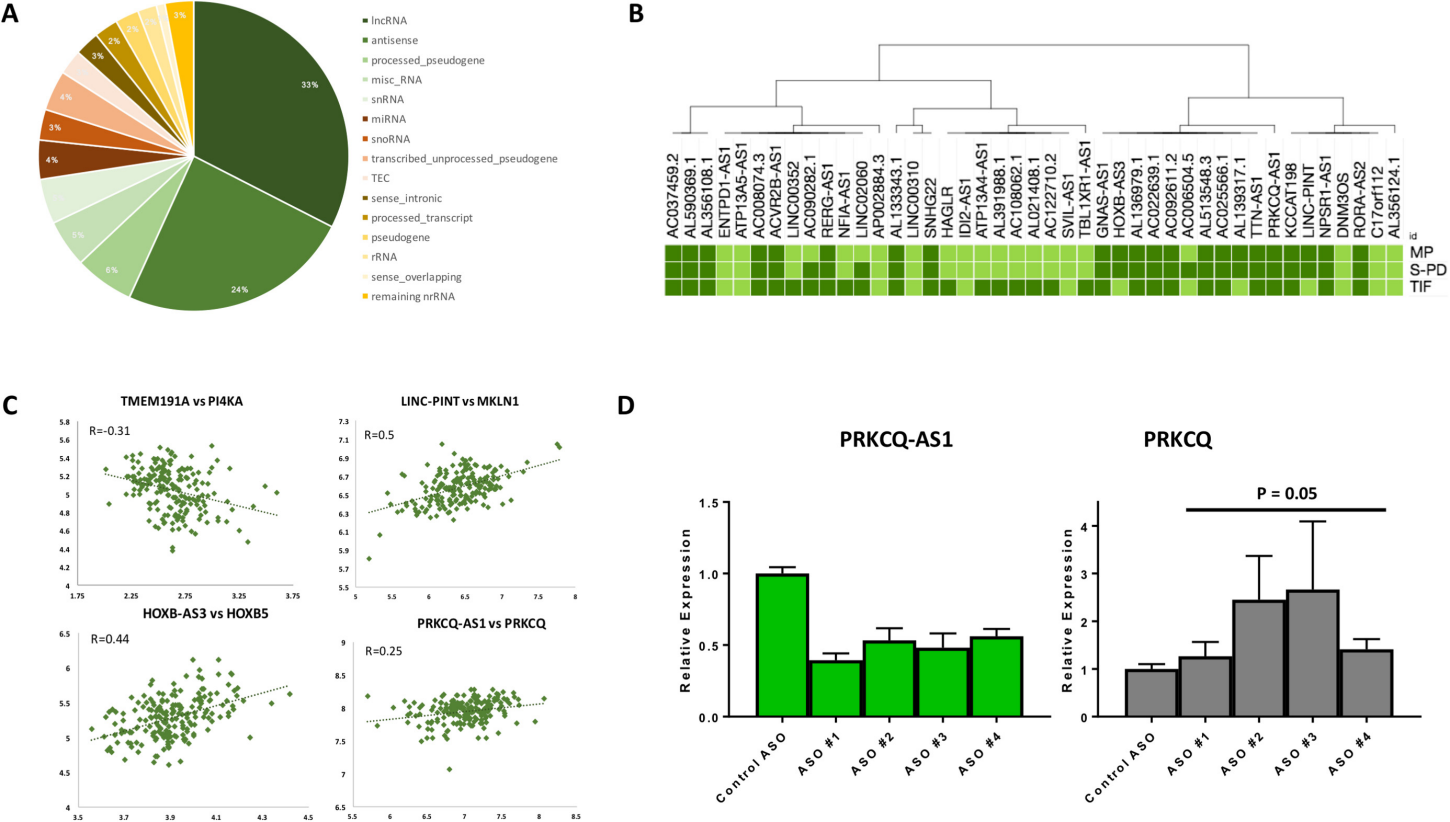
**The human muscle non-coding RNA transcriptome: relationship with insulin sensitivity**. (**A**) The non-competitive nature of RNA quantification and tissue configured CDF for the HTA 2.0 microarray facilitated the first detailed and quantitative view of long non-coding RNA molecule expression in human skeletal muscle *in vivo* (>20 000 ENST representing 16 223 genes (ENSG)). The majority of non-coding RNAs (ncRNAs) identified in human muscle were classed as either ‘anti-sense’ molecules or long non-coding RNA (lncRNA). (**B**) We utilized three clinical cohorts (*n* = 282), with varying ranges of chronological age, to examine which ncRNAs were related to insulin sensitivity (IS). Eighty-six long ncRNA associated with IS, the majority (43) being either antisense or lncRNA (Figure 2B, See Supplemental data S3). The vast majority of these were also expressed in adipose and pancreatic cells (*in vitro* beta cells, Supplemental data S3) indicating they could influence insulin biology across multiple organs. The relationship between each ncRNA and IS was largely consistent in the three sub-groups, with greater variability noted among the oldest subjects, which may reflect population stratification (62). LncRNAs like *NEAT1* and *MALAT1* were very highly expressed and regulate gene expression via interactions with chromatin (139), while the majority of the IS related ncRNAs were expressed at a level similar to coding mRNA. (**C**) Four examples of lncRNA molecules related to fasting IS and how they co-vary with their *cis* expressed protein-coding transcript *in vivo* (*n* = 191, younger samples). The protein-coding transcripts were not themselves significantly correlated with IS. (**D**) Human HepG2 cells (hepatocellular carcinoma cell line) were transfected with 20 nM of phosphorothioate antisense oligonucleotides (PS ASO, one control ASO and four different ASO targeting *PRKCQ-AS1*) in triplicates, and expression of *PRKCQ-AS1* (A) and *PRKCQ* (B) was measured by quantitative real-time PCR. ASO-induced knockdown was statistically significant (*P* < 0.01). Values were normalized to *GUSB/PPIB* and expressed relative to levels in cells treated with control ASO. Values are mean ± SEM.

The majority of the detectable ncRNAs in muscle were classed as ‘antisense’ or ‘lncRNA’ molecules (Figure 2A). To examine which lncRNAs were related to IS, we utilized three sub-cohorts of muscle data produced on the HTA 2.0 array (Table 1, *n* = 282) and found 86 ncRNA associated with IS (Mean FDR = 5%). Half of these were classed as ‘antisense’ or ‘lncRNA’ molecules and were largely consistently expressed in S-PD and MP cohorts, (Figures 1 and 2B); but less so in the cohort of older subjects (TIFIN, *n* = 91). To compare the expression pattern of IS-related muscle ncRNAs to other T2DM relevant human tissue-types, we produced tissue-specific HTA 2.0 CDFs for adipose tissue (37) and human pancreatic cells (45). The majority of the muscle IS-related lncRNAs were expressed at comparable levels in human adipose and islet-cells (Supplemental data S3, and Figure 6 below), so they may plausibly impact on insulin biology and metabolism across multiple organs.

Approximately half of the IS lncRNAs had *cis*-expressed transcripts, most of which were protein-coding (Supplemental data S3). Approximately 30% of these lncRNAs demonstrated a sizeable positive or negative co-expression pattern, across 191 samples, with their *cis* protein coding partner; these included *LINC-PINT, HOXB-AS3, TMEM191A* and *PRKCQ-AS1* (Figure 2C), a natural antisense for the diabetes drug-target *PRKCQ* (codes for PKC-θ) (82–85). We initially sought to study three antisense lncRNAs *in vitro*: *PRKCQ-AS1, NPSR1-AS1* and *RP11–582J16.4* (AC037459.2). All were robustly expressed in both normal hepatocytes and HepG2 cells (insulin target cells) but the partner of *NPSR1-AS1, NPSR1*, was not detected *in vitro* and thus only *PRKCQ-AS1*, and *AC037459.2* were studied. For each lncRNA, four phosphorothioate antisense oligonucleotides (PS-ASO) were designed. The PS-ASOs did not reduce the expression of *AC037459.2*. All four PS-ASO targeting *PRKCQ-AS1*, however, provided ∼50–60% knockdown in the HEPG2 human liver cell line, resulting in increased expression from the *cis*-expressed protein-coding gene, at the time-points examined (24–72 hours; Figure 2D). Given the role PRKCQ plays in insulin signaling, and the extensive complexity of the transcripts expressed from the PRKCQ loci, detailed studies are merited to explore the interaction with each splice-variant and subsequence impact on cell metabolism and signaling.

### CORE-IS list is composed of genes with biochemical and genetic links with type 2 diabetes

**Figure 3.**
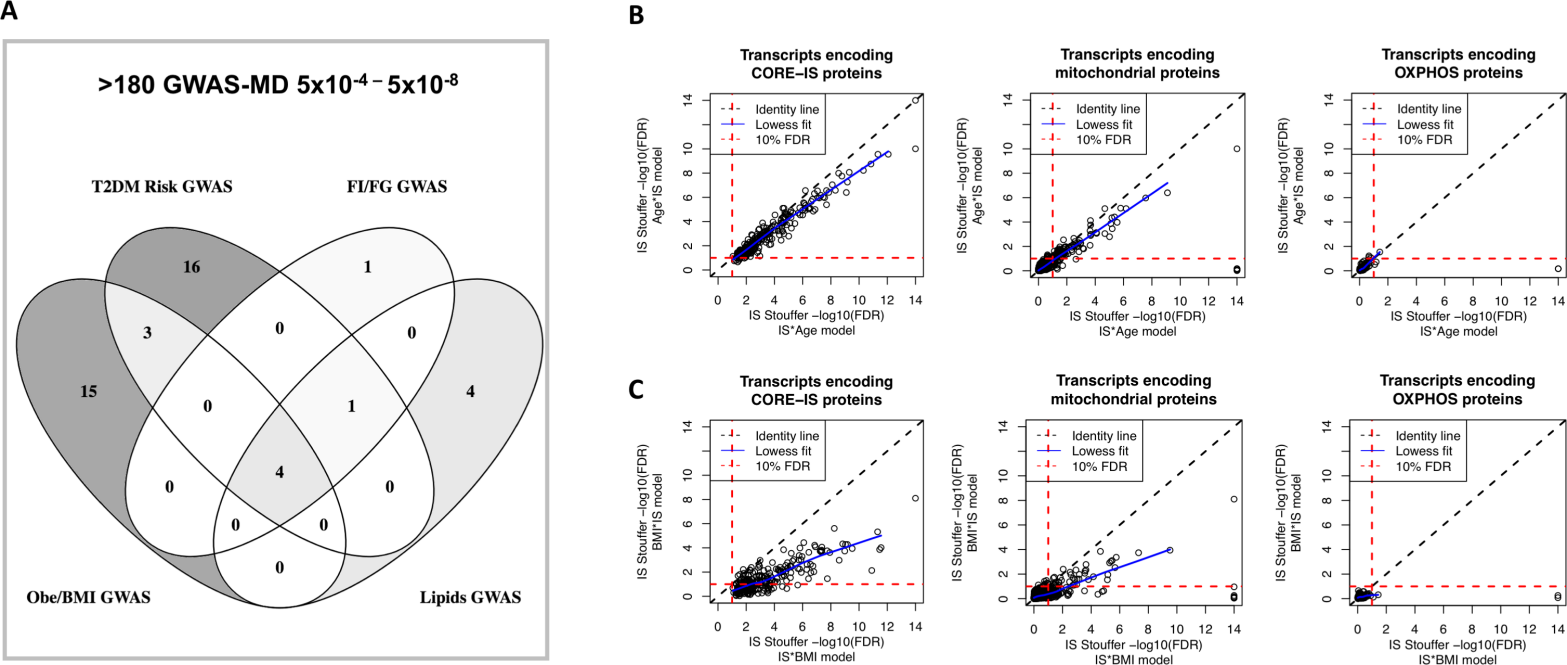
Relationship with GWAS-MD loci and the impact of covariate modeling on insulin sensitivity relationship with gene expression. (**A**) The majority of CORE-IS genes located at genome wide significant metabolic disease loci (GWAS, 5 × 10^−8^), were related to either ‘risk of developing T2DM’ (24) or ‘Obesity/BMI’ (22), partly reflecting the greater number of such loci, over those known to influence fasting insulin or glucose. See Table 2 for details of ∼180 genes; for what the Diabetes consortium defined as marginal GWAS loci (5 × 10^−4^). (**B**) When the relationship between IS and RNA is studied using ANOVA and a covariate, e.g., age or BMI, the model yields *P*-values for both variables, where the outcome depends on the order in the linear model and the inter-relationship between IS and Age/BMI. The log_10_ false discovery rate (FDR) values (using the Stouffer method and a calculated FDR (60)) are plotted for three gene-sets (CORE-IS ENST, mitochondrial located genes and the Mootha *et al*. (26) defined OXPHOS gene-set) from the two different models, IS*age and age*IS. As can be seen, the vast majority of CORE-IS genes remain significant when adjusted for age (i.e. age*IS) as compared to IS*age, but with a small systematic loss in statistical significance. There are only two significant OXPHOS genes, one down-regulated with better IS, and one of which is related to an interaction with age. (**C**) The log_10_ false discovery rate (FDR) values are plotted for three gene-sets (the CORE-IS ENST, mitochondrial located genes and the OXPHOS gene-set) from the two different models, IS*BMI and BMI*IS. As can be seen, there is a systematic loss in statistical significance when BMI is adjusted for (BMI*IS) in the model as compared to IS*BMI. Approximately 38% of the CORE-IS protein coding genes FDR-values are no longer significant when BMI is considered first, in the ANOVA Type I model, while a further 33 are identified, with a proportionate gain in new GWAS-MD loci (mainly for BMI or Obesity) when this alternate model is used.

An extensive literature review, searching for a substantial link between the CORE-IS genes and insulin, diabetes or obesity provided overwhelming support for the validity of our ENST-based analysis (Table 2; for the citations see Supplemental Table S2 within the supplemental materials document). We subsequently established that this subset of literature supported IS genes demonstrated the strongest univariate linear relationship with IS, as a sub-group of all CORE-IS genes (*P* < 0.03, Bonferroni corrected *t* test). To further explore the CORE-IS list we considered the hundreds of genomic loci that have been linked to the risk of developing T2DM or associated with variation in insulin, BMI or glucose homeostasis (7,12,72–76,86,16,65–71). For the majority of these loci it remains unclear which gene (or genes, given the potential for linkage disequilibrium (LD)) represent the causal link (14). Overlaps between the CORE-IS gene list and these GWAS-MD loci would further substantiate our analysis but more importantly help identify which gene underlies the reported genetic association. Indeed, at least 45 of the 332 CORE-IS genes originate from GWAS-MD loci at a genome wide significant level (*P* < 5 × 10^−8^), while a further 180 have suggestive associations according to www.type2diabetesgenetics.org/home/portalHome (see Materials and Methods, Table 2 and Supplemental data S4). The majority of genes linked to either risk of T2DM or risk of Obesity, with a smaller number to more discrete phenotypes (fasting insulin, fasting glucose or circulating lipids—reflective of smaller GWAS samples and/or phenotypes with greater random and technical error) (Figure 3A).

**Table 2.** CORE-IS genes with established links with insulin biology or metabolic disease. A large-scale version of this table is available as supplementary material online

Gene ID	[RNA] relationship with IS	Published Biochemical relationship	T2DM Portal Evidence System (Grey-shade=5x10-8, unshaded=5x10-4)	GWAS Significant (GWAS Catalog/Articles)	Gene ID	[RNA] relationship with IS	Published Biochemical relationship	T2DM Portal Evidence System (Grey-shade=5x10-8, unshaded=5x10-4)	GWAS Significant (GWAS Catalog/Articles)
AASS	positive	> better S%	WC		MAGI3	negative		IS, TG	
ABCG1	positive	> better S%	IS, Adiposity		MAPKAP1	negative	< better S%	WHR	Obesity
ABHD2	negative	< better S%	T2DM, LDL		MAPRE3	negative		FG, TG	T2DM
ACADL	positive	> better S%			MAST3	negative		BMI, WC, WHR	
ACOT11	positive	> better S%	IS, T2DM, BMI, LDL		MAT2B	positive		T2DM, LDL, Adiposity	
ACSS1	positive	> better S%	BMI		MCC	negative		T2DM, IS, HDL, TG, BMI, WC, LDL, HOMA-B	T2DM, Obesity, IR, Lipids
ADPRHL1	negative				MCCC1	positive	> better S%	BMI	
AGL	negative	< better S%	TG		ME2	negative	< better S%	TG	
AKR1B15	negative		WHR		MRC2	negative		T2DM, BMI, IS	
ALDH1L1	positive	> better S%	BMI, LDL, WHR, Adiposity		MROH7	positive		WC	
ALDH1L2	negative		BMI, WC		MROH7-TTC4	positive		T2DM	
ALDH6A1	positive	> better S%			MSRB3	negative		WHR, WC	T2DM
ANKRD50	negative		T2DM		MSTN	negative	< better S%	BMI	
ANKS1B	negative		IS, T2DM, BMI, FG, LDL	Obesity	MTDH	negative		T2DM, Adiposity	
ASCC1	negative		IS		MTF2	positive		HDL (LDL, WHR)	Lipids
ATAD2	positive		T2DM, WHR		MTPN	negative			Obesity
ATP1A1	negative	< better S%	HDL		MTRF1	negative		T2DM	
ATP1B3	negative		HDL		MUSK	negative		T2DM, IS, WHR, Adiposity	
ATP2A2	positive		BMI		MYBPC2	negative		BMI	
B4GALT6	negative	< better S%	IS		MYL1	negative		IS	
BCL2L11	positive		BMI, T2DM, WHR, Adiposity		MYL12A	positive		IS	
BDH1	positive	> better S%	IS, T2DM, BMI		MYLK4	negative		IS, Adiposity	
BRD8	positive	> better S%	T2DM, BMI		N4BP2L1	positive		T2DM, WC, HDL, LDL, BMI	
C15orf41	positive		IS, BMI, WC		NCAM1	negative		T2DM, IS, FG, BMI, HOMA-B	Obesity
C1orf158	negative			Lipids	NDUFAF5	negative	< better S%	T2DM, LDL, Adiposity	
CAB39L	negative		BMI, WHR, WC		NETO2	negative		IS	
CACNB1	negative		T2DM		NIN	negative		BMI, TG, Adiposity	
CALM1	negative	< better S%	IS		NSF	negative	< better S%	BMI, WC, WHR, pro-insulin	
CARNS1	negative	< better S%	BMI, TG		NUCB2	negative	< better S%	WC (BMI, T2DM, WHR)	T2DM, Obesity
CBX7	positive		WC		NUP210	positive		BMI, WC, WHR, HC	
CCDC25	negative		WC		OARD1	positive		HC	
CCDC69	negative		IS, T2DM, TG, LDL		OSBPL6	negative		T2DM, WC, WR, Adiposity	
CCNB1IP1	positive		IS, T2DM		PARK2	negative	< better S%	BMI (T2DM, TG, IS, LDL, WC, Adiposity)	Obesity
CD151	negative		HOMA-B		PCYT2	positive	> better S%		
CD38	negative	< better S%	T2DM		PDHX	negative	< better S%	WC	
CDK14	negative	< better S%	IS, BMI, T2DM, WHR		PDLIM7	negative	< better S%		
CDK2AP1	negative	< better S%	HDL, T2DM, LDL	T2DM	PGK1	negative	< better S%		
CEBPD	positive	> better S%	T2DM		PGM2	negative	< better S%	IS	
CEBPG	negative		IS, WC, HDL, WHR	T2DM	PHKB	negative	< better S%	BMI, WHR	
CERS6	negative	< better S%	FG, (BMI, T2DM)	FG, (BMI, T2DM)	PKM	negative	< better S%	BMI, TG	
CLIP4	positive		BMI		PLXDC1	negative		T2DM, LDL	
COL15A1	negative		TG, WC		PON2	negative	< better S%		
COL4A3BP	positive		BMI, LDL	Obesity	PPM1L	negative	< better S%	BMI, T2DM	
COL5A2	negative		IS, HDL		PPP1R3B	negative	< better S%	T2DM, BMI, TG, WC, LDL, WHR	T2DM, IR, Lipids
COL6A1	negative		T2DM, WHR, FG, LDL	Obesity	PPP2R5C	negative	< better S%	IS, T2DM, BMI, WC, WHR	
CORO1C	negative	< better S%	LDL		PRDX6	positive	> better S%		
CRBN	positive	> better S%	T2DM		PRKAG3	negative	< better S%		Obesity
CTH	positive	> better S%			PRRX1	positive		LDL, WC, WHR	Obesity
CTSF	positive		IS		PRSS42	positive		HDL	
DAAM1	negative		T2DM, BMI, Adiposity, WHR	T2DM	RAB29	negative		n/a	Obesity
DAPK1	negative		IS, HOMA-B, TG, WHR, Adiposity		RAB30	negative		T2DM, IS	
DAZAP1	positive		T2DM (WC)	T2DM (WC)	RAB40C	positive		FG, WC, HC	
DDR1	positive	> better S%	T2DM, LDL, TG, Adiposity	T2DM	RAPH1	negative	< better S%	T2DM, LDL	
DHRS7	negative	< better S%	BMI, LDL		RCBTB1	negative		IS, BMI	
DHTKD1	positive	> better S%	TG, WHR	T2DM	RDH10	negative	< better S%		
DPYSL3	positive		T2DM		RND3	negative	< better S%	T2DM, LDL, BMI, WC	T2DM
E2F3	negative		T2DM, BMI, LDL, WC	T2DM	RNF10	negative	< better S%	BMI, WC	
ECT2	negative		BMI		ROBO1	negative	< better S%	IS, BMI, WHR, WC, Adiposity, LDL, HDL	
EDEM3	negative		T2DM		RXRG	negative	< better S%		
ELP2	positive		TG, HDL, FG, WHR		SATB1	positive		WHR, WC	
EML4	negative		T2DM (BMI, LDL, IS, FG, WC)	T2DM (BMI, LDL, IS, FG, WC)	SEC61A1	negative	< better S%		
EPDR1	negative		IS		SEPT11	negative	< better S%	FG, BMI, Adiposity	
ERBB3	negative	< better S%	WC		SESN1	positive		T2DM, LDL	
EZH1	positive		WHR		SESN3	negative	< better S%	LDL	
FAM118B	negative		LDL		SETDB2	negative	< better S%	IS	
FAM160A1	positive		IS, Adiposity		SFRP4	negative	< better S%	IS, WHR, WC	
FARP2	negative		TDM, HDL		SGCG	positive		T2DM, IS, LDL, TG, WHR	T2DM
FAT1	negative		IS, LDL, BMI	Obesity	SGMS2	negative	< better S%	T2DM	
FERMT2	negative		IS, FG, BMI, Adiposity		SH3PXD2A	positive		T2DM, IS, Adiposity, WC	
FOXP2	positive		T2DM, BMI, IS, Adiposity, WHR	Obesity	SH3RF2	negative		T2DM, BMI, WHR	
FRMD3	negative	< better S%	T2DM, HDL, BMI, LDL		SHISA2	negative		HC	
G0S2	negative	< better S%	FG		SHISA4	negative		BMI (T2DM, IS)	BMI (T2DM, IS)
GADL1	negative		IS, BMI, LDL, WHR	T2DM	SLC16A10	negative	< better S%	T2DM, BMI	
GCLC	positive		T2DM, IS, BMI, WHR, TG		SLC27A1	positive	> better S%		
GLUL	positive	> better S%			SLC43A1	positive	> better S%	LDL, WC, WHR	
GPAT3	positive	> better S%	n/a		SLC4A4	negative		T2DM, IS, BMI, WC	
GPD1	negative	< better S%	WC		SMIM8	positive		LDL	
GRB14	negative		T2DM, IS, LDL, HDL, TG, WHR	T2DM, IS, LDL, HDL, TG, WHR	SMTNL2	negative		BMI	
GRIA3	negative		TDM, Adiposity		SOS2	negative		IS, Adiposity	
GSR	negative		T2DM, IS, HOMA-B		SPARC	negative	< better S%	WHR	
HBP1	positive	> better S%	TG, WC		SSX2IP	negative		FG	Obesity
HEXIM1	negative	< better S%	Adiposity		STK38	negative		T2DM	
HNRNPD	positive		WC		STK38L	negative		BMI, LDL, WHR	
HOMER1	negative	< better S%	IS		TACC2	positive		T2DM, IS, BMI, WHR, WC, Adiposity	
HOXB-AS3	positive			Obesity	TANC2	negative		T2DM, BMI, LDL, WC, WHR	
IGF1R	positive	> better S%	T2DM, BMI, WC, FG, LDL, WHR	Lipids	TPRXL	positive		T2DM, Adiposity	
ING5	positive		WHR, WC		TSPAN15	negative		WHR	T2DM
INSR	positive	> better S%	TG (T2DM, BMI, HOMA-B, HDL)	T2DM, Obesity, IR, Lipids	TSPAN3	negative		T2DM	T2DM
ITIH5	negative	< better S%	T2DM, WHR, FG		TSPAN9	negative		BMI, HDL, WHR, Adiposity	
KCNN3	negative	< better S%	T2DM, IS, WC		TTC8	negative		T2DM	
KIAA1109	positive		T2DM, IS, BMI, WHR, TG		TUBGCP4	positive		IS, TG, BMI, T2DM	
KIAA1841	positive		T2DM		TULP4	negative		T2DM, IS, WC	
KLF12	positive		T2DM, IS, BMI, FG, LDL, WHR, TG	T2DM, Obesity	UBE2G1	positive		HDL	
KLF15	positive	> better S%			UBE4A	positive		T2DM, HDL	
KLHL2	negative		T2DM, WHR		UNKL	positive		IS, WC	
KLHL31	negative		T2DM, WHR, Adiposity, HOMA-B	Obesity	USP25	positive		FG, WC, HC	
KPNA2	negative		T2DM, TG		VCL	negative		T2DM, BMI, Adiposity	
KPNA5	negative		IS, BMI, HOMA-B		VPS8	positive		FG	
LAMB2	negative		IS		WWOX	negative		T2DM, IS, FG, TG, BMI, WHR, WC, Adiposity	T2DM, Obesity
LDHA	negative	< better S%	FG, WHR		XPO4	positive		T2DM, HDL, HC	
LDHB	positive	> better S%	T2DM, WC		ZEB1	negative		WC	
LGALS1	negative	< better S%			ZFAND5	positive		WHR	
LGR5	positive	> better S%	T2DM, WC	T2DM	ZMAT3	negative		T2DM	
LIN52	positive		T2DM, BMI		ZMYM2	negative		WHR	
LPL	positive	> better S%	TG, HDL (T2DM, LDL)	T2DM, Obesity, IR, Lipids	ZNF274	positive		Adiposity	Lipids
LRRFIP2	negative		FG		ZNF282	positive		WC	
LRRN3	negative		T2DM, Adiposity						

Two-hundred and 13 genes from 332 have prior links with T2DM phenotypes (54 GWAS-Significant for T2DM phenotypes, 82 with functional/biochemical studies linking to insulin resistance, biology or metabolic disease and 180 GWAS ‘suggestive’ associations (5x10-4). Adiposity = linked to adipose mass phenotype. BMI = Body mass index. FG = Fasting glucose. HDL = HDL cholesterol. IS = measure of insulin sensitivity. LDL = LDL cholesterol. Pro-insulin = pro-insulin levels. TG = Triglycerides. WC = Waist circumference. WHR = Waist-to-hip ratio. T2DM = Risk for T2DM. () = at the lower significance. Data resources are: http://www.type2diabetesgenetics.org/home/portalHome, https://www.ebi.ac.uk/gwas/home and Pubmed (June-July 2017).

Substantial differences in age, aerobic fitness etc. between T2DM cases and controls, especially in combination (23,24,87), will yield gene expression differences inappropriately assigned to T2DM or IR, as well as potentially obscuring genuine differences. For some, but not all, of our cohorts, age or BMI modestly covaried with IS (Table 1). When examining the results of the ANOVA analysis after first considering the co-variate (‘adjusting’), there was no overall impact of Age on IS gene selection except for some slight reductions the FDR achieved for some mitochondrial genes (Figure 3B). Notably, there was no IS dependent expression pattern for oxidative phosphorylation (OXPHOS) genes regardless of which ANOVA model was used (Figure 3B). In contrast, when BMI was utilized to ‘adjust’ the ANOVA model for IS, a number of CORE-IS genes were removed (Figure 3C) while an additional 33 genes passed the FDR threshold (Supplemental data S4), of which seven occurred at GWAS significant loci for obesity or blood lipids, but none for the other phenotypes. The genes lost from the CORE-IS, when the ANOVA model assigned covariance between IS and BMI to the covariate, included nine genes from Obesity risk loci, eight genes from T2DM risk loci and two others. This demonstrates that neither approach to linear modeling is fully compliant with the complexity of the biological relationship between BMI and insulin as the second ANOVA model enriched the IS correlated gene list with genetic loci associated with obesity rather than insulin. Finally, the lack of any relationship between OXPHOS gene expression and IS (26) supports recent mechanistic studies dissociating OXHPOS status and insulin signaling (88). We did, however, observe the expected univariate correlation of OXPHOS genes with chronological age and aerobic capacity.

### Response of the CORE-IS genes to therapeutic clinical interventions

**Figure 4.**
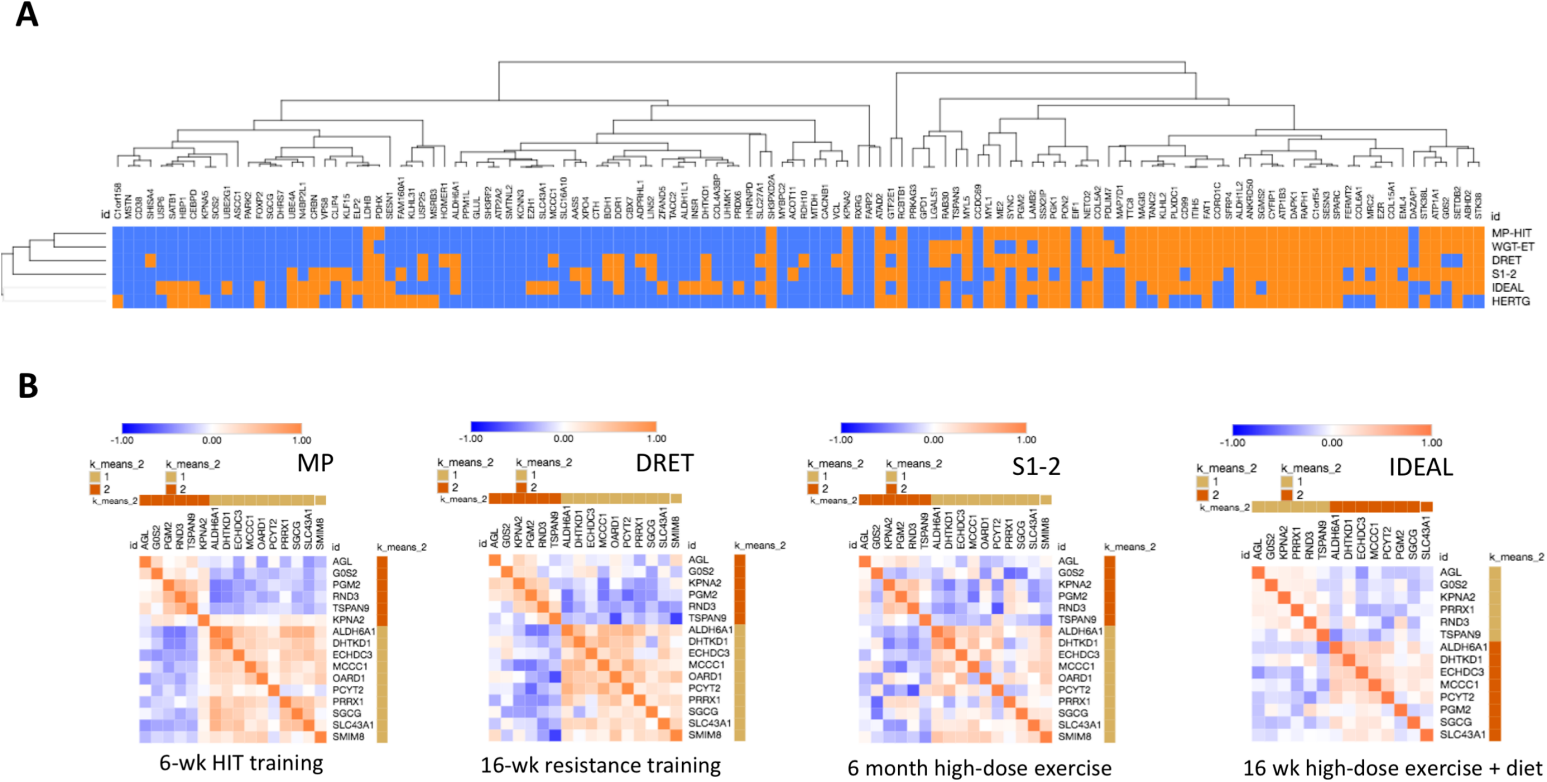
Treatment-responsive insulin-sensitivity genes. Supervised exercise-training (with or without calorie restriction) can improve peripheral IS (48,50) and is considered the primary tool to reduce the risk of developing type 2 diabetes. If the Core-IS genes regulate IS they should respond to clinical interventions and do so in a manner consistent with the observed improvement in IS. (**A**) Differential gene-expression for 332 Core-IS genes was calculated, across six intervention studies (three were independent of the baseline IS analyses), *P*-values aggregated using the Stouffer method; and a false discovery rate (FDR) was calculated (60). Approximately 40% of genes were differentially regulated (FDR < 5%) by treatment, and critically in a direction consistent with their observed relationship with fasting IS, and despite the highly divergent nature of the exercise/life-style modification interventions. (**B**) The relationship between the magnitude and direction of CORE-IS gene expression and the magnitude and direction of IS responses to clinical intervention were calculated using Spearman rank correlation. Individual HOMA2-derived IS were not available for the HERTG (insulin assay) or the Weigert *et al*. (38) (data availability) studies. This lead to the identification of a subset of 16 genes, from ∼250, that were consistently correlated with change in IS, in all the four independent studies. The 16 genes segregated into two subsets—one (*DHTKD1, SLC43A1, PCYT2, MCCC1, SGCG, ECHDC3, ALDH6A1, SMIM8* and *OARD1*) positively associated with improvements in IS, and one (*PGM2, RND3, G0S2, AGL, TSPAN9* and *KPNA2*) negatively associated. Many CORE-IS genes, especially belonging to network 1 and 2 (Figure 5 and Table 3) were also, on average, DE following 3 months of a PPARγ agonist (Supplemental data S5, (56)) - unfortunately no individual IS values were accessible from the authors to study the quantitative correlative relationship with IS.

Supervised exercise-training (with or without calorie restriction) can improve IS in humans, and is considered a primary prevention strategy for reducing risk of T2DM (89). Thus, if the CORE-IS genes are responsible for regulating peripheral insulin sensitivity, treatment should cause differential expression (DE) of the CORE-IS RNA in muscle. Six independent life-style intervention studies (Supplemental Table S1) were used for meta-analysis and an FDR was calculated (60) (Figure 1)). We found that at least 135 (>40%) of the protein-coding IS-CORE genes were differentially regulated in the clinical intervention studies (meta-analysis FDR < 5%) *and* in a consistent direction (Figure 4A, Supplemental data S5 and Supplemental Figure S3). Further, in a seventh study where 12 weeks of PPARγ agonist drug-treatment improved IS (56), 70 protein coding IS genes were DE (*P* < 0.05 using a *t*-test, Supplemental data S5, i.e. ∼29% of the detected CORE-IS genes). In the two clinical intervention studies where HTA 2.0 array-data were available, 20 of the CORE-IS ncRNAs were DE by treatment (Supplemental data S5). Thus, there is substantial evidence that the CORE-IS genes are regulated by life-style interventions aimed at treating and/or preventing T2DM.

While DE analysis further supports a direct role for the CORE-IS genes regulating IS in humans, the most important regulators of IS are likely to be regulated in proportion with the change in clinical status. Individual pre- and post-intervention IS values were available for four of the intervention studies. Spearman rank correlation coefficients were calculated for delta CORE-IS gene-expression versus delta IS, identifying 16 genes consistently regulated in *proportion* with the clinical improvement in all four independent studies (Figure 4B, cumulative binomial probability, *P* = 0.0000053) and, critically, in a directionally consistent manner with the fast IS relationship. These 16 genes segregated into two clusters, one positively (*DHTKD1, SLC43A1, PCYT2, MCCC1, SGCG, ECHDC3, ALDH6A1, SMIM8* and *OARD1*) and the other negatively associated (*PGM2, RND3, G0S2, AGL, TSPAN9* and *KPNA2*) with improvements in IS. PPARγ agonist drug-treatment collectively tended to influence these 16 genes more than the average across the entire CORE-IS gene list (median *P* = 0.07 versus *P* = 0.18). Conserved transcription factor (TF) binding-site analysis (63) identified RREB1 and INSM1 binding-sites (*Z*-score 13.9 and 11.6) in the 16 genes activated in *proportion* to the improvement in IS (Supplemental Figure S4). These binding sites were not enriched in the (larger) set of ‘all exercise-responsive’ CORE-IS genes nor in the entire 332 Core-IS gene list (Supplemental data S6), suggesting a more specific relationship with these 16 IS genes ('Delta-IS'). In the two studies examining ncRNAs, some changed in proportion to clinical improvements (e.g. *RORA-AS2, SNORA70, AL136979.1, PRKCQ-AS1, SNORD83A, ATP13A4-AS1* and *NPSR1-AS1*). In each case the pattern of change was positively correlated with changes in fasting IS (Supplemental data S6).

The treatment regulated CORE-IS genes can be further investigated by ‘guilt-by-association’ (GBA) analysis (80), relying on completely independent data (81). We tested the hypothesis that the nine CORE-IS genes positively correlated with a change in IS in intervention studies and the transcription-factors distinctly associated with this subset of Delta-IS genes, could regulate an additional ‘layer’ of genes responsible for regulating IS. Multi-tissue (81) analysis identified 52 genes (*P* < 1 × 10^−6^) co-expressed with Delta-IS genes (Supplemental data S7). This 52-gene subset was 77-fold enriched for branched-chain amino acid (BCAA) catabolic genes (*P* < 1 × 10^−5^) located in the mitochondrial matrix, and included nine additional GWAS-MD loci beyond the 45 already in the CORE-IS list (*ALDH2, C3, CD36, HNF4A, HMGCS2, KHK, IDH2, RREB1* and *SERPINA6* (65)). The cluster of genes covarying with the 7 negatively correlating Delta-IS genes contained a further two GWAS-MD loci (*SLC2A4* and *PPARG*). Thus, both the GBA analysis and the CORE-IS analysis identified genes linked to GWAS-MD and in particular, catabolism of BCAAs (valine, leucine and isoleucine) and aromatic amino acids (phenylalanine and tyrosine). Indeed, studies from 1960 onwards have identified associations between circulating levels of BCAA and aromatic AA with IR (47,90). More recently, pre-clinical studies identified that catabolism of valine may act as a signal to promote muscle lipid uptake (91). Here, we show that altered muscle gene expression in the AA catabolic pathways occurs in the absence of any systematic loss of mitochondrial OXPHOS gene expression (Figure 3B).

### Networks analysis reveals the inter-relationship between the GWAS-MD enriched IS genes

We next examined if the CORE-IS genes formed robust networks, and if protein–protein (P–P) interaction analysis, relying on further independent data (79), would demonstrate that each ‘gene’ network contained proteins known to physically interact (78). Typically, genes from validated GWAS loci are used to weight nodes within gene-expression networks (5), however, in this case we used the identified RNA-RNA network relationships to gain insight into how GWAS-MD loci genes may interact, within the quantitative and continuous framework of RNA abundance. Moreover, this strategy allowed us to associate ncRNAs (mostly of unknown function) with specific proteins and hence biochemical pathways. Using MEGENA (77) we identified seven discrete planar filtered networks (Table 3) (*n* = 191, FDR <1% for Spearman correlation; *P* < 0.01 for module significance and *P* < 0.01 for network connectivity).

**Table 3.** Summary of CORE-IS RNA networks

Network	Size	Relation to IS	Biotype	P–P interaction profile	GBA transcriptome profile*
1 Figure 5A	87	64% negative	13% ncRNA	Insulin receptor signaling pathway (FDR<1 × 10^−5^ 6.7 FE); interaction with host (FDR<1 × 10^−4^, 4.0 FE)	Extracellular matrix organization (FDR<1 × 10^−32^, 5.3 FE)
2 Figure 5B	81	75% negative	22% ncRNA	Positive regulation of nucleobase-containing compound metabolic process (FDR < 1 × 10^−8^, 3.0 FE); positive regulation of metabolic process (FDR <1 × 10^−7^, 2.3 FE)	Muscle filament sliding (FDR < 1 × 10^−35^, 17.1 FE)
3 Supplemental Figure S5	83	66% negative	20% ncRNA	Interaction with host (FDR < 1 × 10^−7^, 6.2 FE); enzyme linked receptor protein signaling pathway (FDR < 1 × 10^−7^, 3.6 FE)	n/s
4 Figure 6	49	16% negative	70% ncRNA	Viral reproductive process (FDR < 1 × 10^−48^, 5.5 FE); translational initiation (FDR < 1 × 10^−37^, 8.7 FE)	n/s
5 -	32	13% negative	19% ncRNA	Protein catabolic process (FDR < 1 × 10^−12^, 3.1 FE)	n/s

MEGENA planar filtered networks were produced using 191 HTA 2.0 gene-chip samples (See methods). protein–protein interaction analysis was carried out using www.networkanalyst.ca and the IMEx Interactome (79). The guilty-by-association analysis (80) used GeneFriends (81). *when using a database for GBA analysis, it is expected that co-correlation will occur between gene expression from the input tissue type (regardless of physiological status), and with genes in that tissue type within the data-base. This is a type of sampling bias that skews Gene Ontology analysis when inputting large lists from distinct tissues (in this case skeletal muscle).

**Figure 5.**
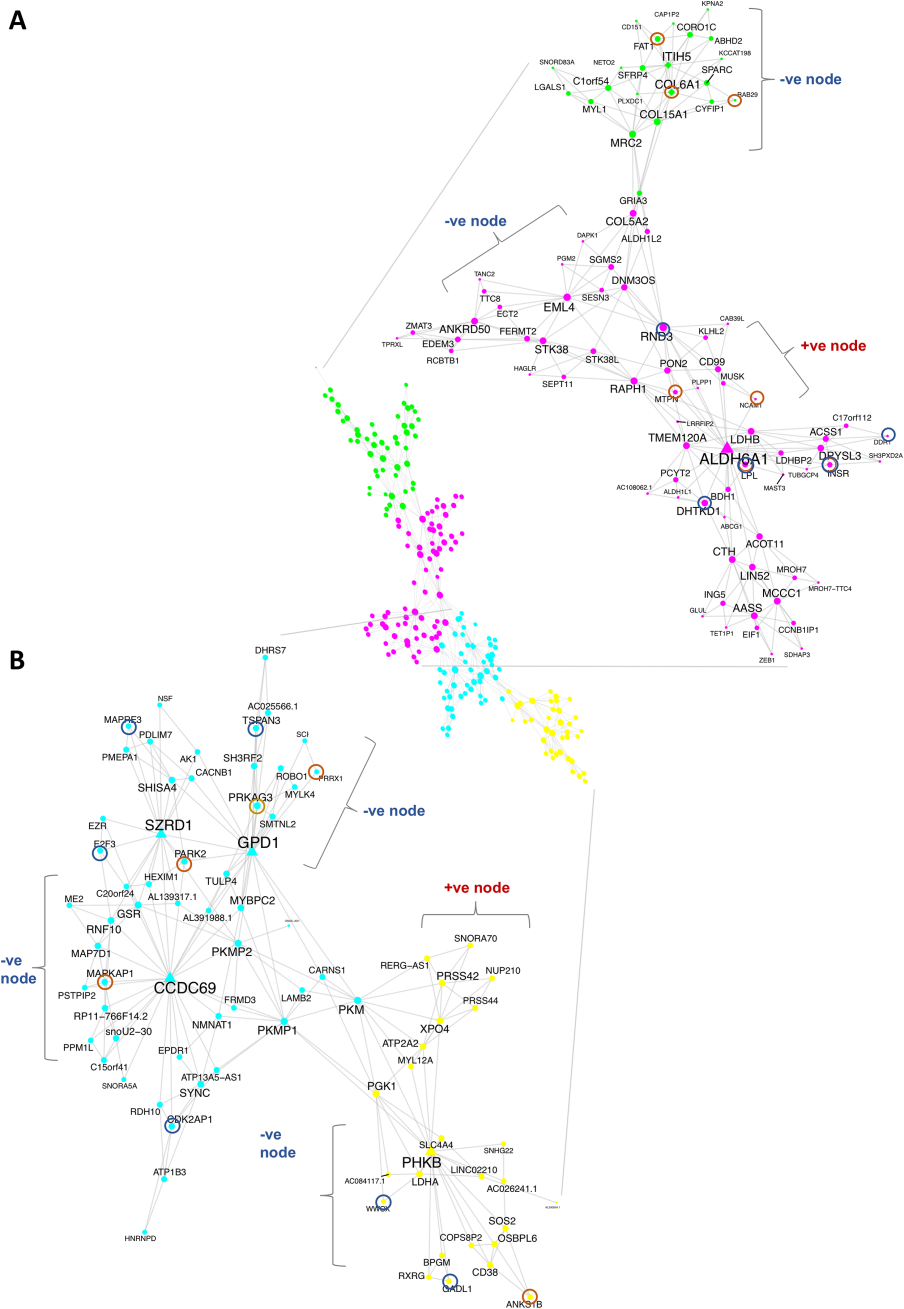
Treatment-responsive IS networks regulate human peripheral insulin sensitivity and link to metabolic disease risk genes. Probabilistic networks have been utilized to establish a causal relationship between molecules and disease (5). We utilized the dynamic response to diverse forms of treatment aimed at reversing insulin resistance, along with measured fasting relationships to estimate if planar filtered networks (77) derived from 191 of the younger individuals (<70yr), were enriched in genes dynamically regulated or were lncRNAs, harboring causal genetic variant related to metabolic disease, or dominated by proven T2DM disease pathways. MEGENA (77) was used to identify discrete planar filtered networks using the CORE-IS gene list as input (FDR <1% and spearman rank correlation; *P*<0.01 for module significance and *P*< 0.01 for network connectivity). Follow-up protein–protein interaction analysis (79) facilitated the interpretation of the identified networks providing additional biological plausibility to each network (78). Node and label size are proportional to the node degree value within each distinct module. (**A**) represents the largest discrete planar filtered network (87 genes, 13% ncRNA and 64% negatively co-varying with *in vivo* fasting IS) that protein–protein interaction analysis identified as being involved with hormone signaling, including insulin, FDR<1%. This network contained 44% (7; *ALDH6A, DHTKD1, KPNA2, MCCC1, PCYT2, PGM2* and *RND3*) of the 16 genes regulated *in vivo* proportional to changes in IS, across four independent treatment studies. Further, 33% of the life-style treatment-response genes (*ALDH6A DHTKD1, PCYT2* and *RND3*) were responsive to three months of PPARγ agonist, given to insulin resistant subjects ((Supplemental data S5, (56)). There were numerous significant GWAS loci for T2DM (gene ID enclosed in blue-ring) or Obesity (gene ID enclosed in brown circle). (**B**) The second largest and discrete planar filtered network (81 genes, 22% ncRNA and 75% negatively co-varying with *in vivo* fasting IS) contained genes from GWAS loci for T2DM, Obesity and Lipids (gene ID enclosed in gold circle). Twenty-five percent of this network was responsive to anti-diabetes drug treatment (PPARγ agonist), given to insulin resistant subjects for three months (56). Protein–protein interaction analysis indicated that this module was enriched by genes (33 in the network, FDR < 1 × 10^−5^) involved in the positive regulation of RNA metabolism and transcription.

The largest discrete planar filtered network contained 87 CORE-IS genes (13% ncRNA) with an enrichment in genes negatively co-varying with *in vivo* IS (64%, *cf* 30% of *all* CORE-IS genes demonstrated a negative co-variation with IS, Table 3 and Figure 5a). Protein–protein interaction analysis characterized the function of the protein-coding members of a minimum network (79) as ‘hormone signaling including Insulin’ (Table 3); and included the insulin-receptor, itself. This network contained 7 (*ALDH6A, DHTKD1, KPNA2, MCCC1, PCYT2, PGM2* and *RND3*) out of the 16 CORE-IS genes regulated *proportionately* to changes in IS across four clinical studies. Remarkably, ∼50% of these protein-coding genes were also DE following anti-diabetes drug-treatment (56); including *ALDH6A DHTKD1, PCYT2* and *RND3* (this compares with <5% of the transcriptome responding to drug -treatment (56)). A module centered around *ALDH6A* (encoding methyl-malonate semi-aldehyde dehydrogenase), a protein which catabolizes valine. Closely co-expressed with *ALDH6A*, was the insulin-receptor, and *MCCC1* (methyl-crotonoyl-CoA carboxylase 1, which is involved in leucine catabolism (92,93)), and *AASS* (alpha-aminoadipate delta-semialdehyde synthase, which catabolizes lysine). DHTKD1 (dehydrogenase E1 and transketolase domain containing 1) also catabolizes amino-acids, as does ALDH1L1. In each case, greater (BCAA catabolising) gene expression was associated with better IS. Seven members of the network overlapped with genes from GWAS significant Obesity loci (Figure 5A, brown circles) and five were from GWAS significant T2DM-risk loci (blue circles). Many of the remaining members of the network are involved in metabolism; with clear roles in T2DM (glycolysis or glycogen break-down, *LDHB* or *PGM2*). Thus, the first discrete network is strongly modulated by all clinical interventions, and biochemically linked to amino-acid catabolism.

The second discrete network (81 genes) was composed of 22% ncRNA (Table 3 and Figure 5B) and was predominantly *negatively* associated with IS (75%). A third of the protein-coding components were responsive to PPARγ agonist drug-treatment (56); P–P interaction analysis indicated the network included genes involved with ‘positive regulation of metabolic process’ (FDR < 1 × 10^−7^, 2.3-fold enriched (FE), *n* = 44 genes) and ‘positive regulation of nucleobase-containing compound metabolic processes’ (Table 3). Five members of the network overlapped with genes from GWAS significant Obesity loci (Figure 5B, brown circles) and 6 were from GWAS significant T2DM-risk loci (blue circles) with one at a GWAS loci associated with lipids (gold circle). *CCDC69* is a network 2 hub gene (coiled-coil domain containing 69, a cytokinesis protein (94)) and it regulates the cell-cycle, as does the GWAS-linked *CDK2AP1*. Given that the majority of skeletal muscle nuclei are post-mitotic, the gene–environment interactions and/or genetic influence we note in muscle may also be reflective of events in other tissue-types, e.g. ß-cells and/or adipocytes. Indeed, the lncRNA *LINC02210* expression in muscle is positively related to IS but more highly expressed in ß-cells as compared with muscle or adipose tissue (Supplemental data S3). In sum, this second network appears to reflect reduced activity of processes that may ultimately reflect muscle use: muscle remodeling and substrate cycling as 47 genes were differentially expressed by exercise.

The third network (Supplemental Figure S5) contained eight genes from loci identified in T2DM and insulin sensitivity GWAS, with a further three linked with obesity and fasting glucose GWAS loci. While 66% of genes had a negative relationship to IS, there was no enrichment in ncRNA (20% ncRNA). P–P interaction analysis contained 33 protein-coding genes involved with the ‘positive regulation of cellular metabolic processes, signaling’ (FDR < 1 × 10^−5^, 2.3 FE) and ‘interaction with host’ (Table 3). Interestingly, the majority of this network was non-responsive to (‘resistant’) PPARγ agonist treatment (56). However, it did contain 4 genes out of the 16 genes regulated *in vivo* following therapeutic lifestyle interventions (Figure 4B) in *proportion* to changes in IS (*GOS2, AGL, LAT3* and *ECHDC3*). Network 3 included a number of insulin- and lipid-related signaling molecules (*B4GALT6, CDK14, CERS6, GRB14, FATP1, GOS2, IGF1R, NLK, SLC27A1* and *SESN1*) (95–102). *GRB14* has been consistently linked to ‘metabolic disease’ and is negatively correlated (*R* = –0.36) with IS in the present and other published analyses (103–105). The network also included regulators of carbohydrate homeostasis, (amylo-alpha-1,6-glucosidase, 4-alpha-glucanotransferase (AGL), a glycogen debranching enzyme) which were negatively associated with IS. Down-regulation was associated with improved IS following clinical intervention. Increased *SLC27A1* (i.e. fatty acid transport protein 1, FATP1), a plasma membrane long-chain fatty acid transporter activated by mTORC1 signaling (98), and less *G0S2* (G0/G1 switch 2)—a negative regulator of lipase activity (and a treatment response gene)—was related to better IS, expanding the evidence base in humans, for the ‘lipid paradox’ and muscle health (106). Thus, network 3 contains genes influenced by ‘substrate turn-over’ (exercise) rather than metabolic signaling (PPARγ activation) with clear links to substrate storage and utilization.

**Figure 6.**
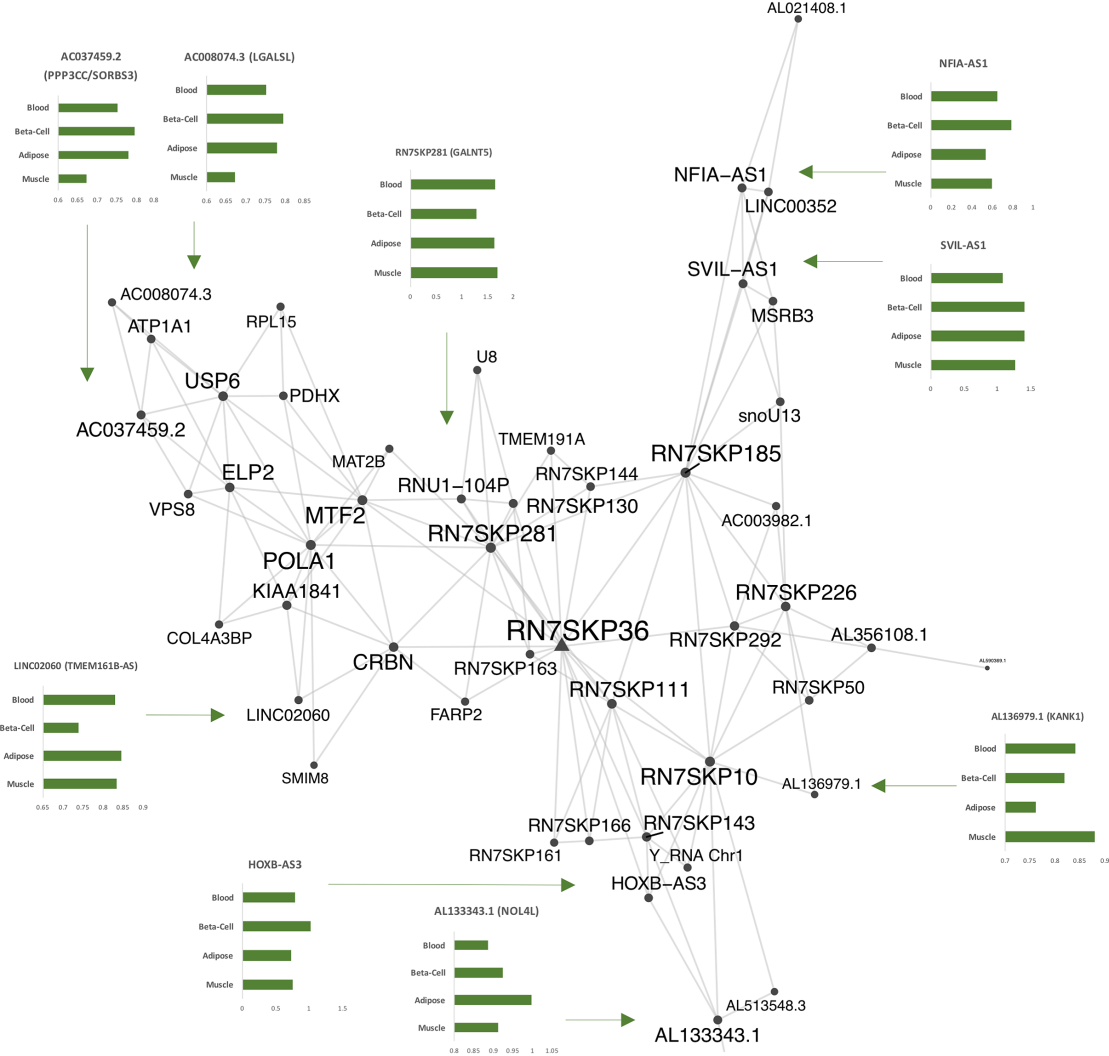
A noncoding RNA network is positively linked to peripheral insulin sensitivity and translational control. Probabilistic networks are utilized to establish a causal relationship between molecules and disease (5). We utilized the response to diverse forms of treatment aimed at reversing insulin resistance, along with measured fasting relationships to estimate if planar filtered networks (77), based on data from 191 individuals, were enriched in genes dynamically regulated or were lncRNAs, harbored causal genetic variant related to metabolic disease, or dominated by proven T2DM disease pathways. MEGENA (77) was used to identify discrete planar filtered networks using the CORE-IS gene list as input (FDR<1% and spearman gene correlation; *P*< 0.01 for module significance and *P*< 0.01 for network connectivity). Protein–protein interaction analysis (79) facilitated the interpretation of the identified networks while providing additional biological plausibility to each module (78). Node and label size are proportional to the node degree value within each distinct module. This, the fourth large discrete planar filtered network was dominated by non-coding RNAs (49 genes, 70% ncRNA, and 84% of the network members positively co-varying with *in vivo* IS). Thirteen of the ncRNA had *cis* antisense expressed protein-coding genes and these 13 genes, along with the remaining 30% protein-coding CORE-IS genes in the network, formed a protein–protein interaction network strongly associated with viral responsive protein-coding genes (*P*< 1 × 10^−48^) and translational initiation (*P*< 1 × 10^−37^). The network included several shorter ncRNAs, including the highly conserved 7SK ncRNA pseudogenes which are 271 to 326 base-pairs long. The function of these is unknown despite the 7SK ncRNA gene itself being described half a century ago. 7SK RNA binds to HEXIM1 to inhibit the positive transcription elongation factor b (P-TEFb)—transcript elongation (*HEXIM1* is negatively associated with IS, Table 2 (140) and also regulated by a lncRNA (141).

A fourth network was dominated by non-coding RNAs (70%, 34 out of the 86 ncRNAs included among CORE-IS gene list, Table 3 and Figure 6), and required information gained from GBA and sense–antisense protein-coding relationship analysis to provide some biochemical context. Twelve of the lncRNAs had a total of 16 antisense *cis*-expressed protein-coding partners demonstrating positive (6) or negative (10) co-expression (Supplemental data S3). The combination of these 16 *cis*-expressed protein-coding genes and the 15 protein-coding CORE-IS genes from the module were analyzed for P–P interactions (79). This yielded a distinct interactive proteome: four gene-sets (mainly represented by the same 55 genes) were defined as representing ‘SRP-dependent co-translational protein targeting to membrane’, or ‘viral transcription’; >60 times enriched over the genome (Bonferroni < 1 × 10^−83^). A second group of 38, non-overlapping from the first, were defined as belonging to ‘viral process’ (19-fold enriched over the genome, Bonferroni < 1 × 10^−34^). Gene ontology analysis using DAVID, confirmed these extremely enriched categories (Supplemental data S8). This ncRNA-dominated network included some classic metabolic genes, (*COL4A3BP* — also known as *CERT* or *GPBP*), a GWAS loci gene for obesity, and a protein shuttling ceramides and diacylglycerol. Plausibly, greater *CERT* expression combats the negative impact of lipid molecules on insulin signaling (106,107) and thus may help to distinguish muscle lipid profile in T2DM patients from the ‘similarly’ highly lipid-laden but high IS state of endurance trained muscle (108), revealing further molecular details of the ‘lipid paradox’ (106).

## DISCUSSION

Tissue responses to the hormone insulin (i.e. IS) underpin glycemic control in humans. Skeletal muscle is the largest target organ for insulin in humans, and in the post-prandial state insulin promotes cellular glucose transport for *non-oxidative* storage as glycogen. It is a striking observation that various modes of exercise or drug treatment (50,109–112) alter IS to a highly variable extent (50,111) such that it is essential we generate greater understanding of the molecular regulation of IS. In the present study, we have established the first robust gene expression signature for IS and utilized three strategies to validate our findings and explore the functional implications of our observations: systematic literature review, quantification of responses to clinical interventions; and application of systems biology methods. We independently identified many known IS regulators, including *GRB14*, a receptor tyrosine kinase adaptor protein (113) which inhibits insulin signaling (105,114), located at a GWAS significant metabolic disease loci, while providing the first detailed map of the relationship between lncRNAs and IS. We may have expected a greater number of GWAS loci related specifically to insulin, however as noted above, linear modeling does not fully capture the complex interactions between genes, insulin, obesity and risk of development of T2DM. Further, GWAS have revealed rather limited numbers of candidate loci for fasting insulin, perhaps reflecting a lack of a standard approach to measuring fasting insulin (59) across GWAS cohorts. Overall, we clearly demonstrate that quantitative modeling of RNA can reveal biological features consistent with both DNA analysis and biochemical analysis, demonstrating a close and expected (115) relationship between these three molecular universes (Table 2).

### Biochemical and physiological influences on the molecular associates of IS

Studying the influence of associated risk-factors for T2DM, such as self-reported physical activity or diet is fraught with challenges (116) making the study of the molecular nature of IS in humans very challenging. Preliminary transcriptomics studies in muscle from T2DM patients claimed to find a specific PGC1α-mediated down-regulation (26) of OXPHOS genes, concluding that this was a therapeutic-target for preventing T2DM (117). However, this observation did not generalize to larger clinical cohorts (22); while any potential impact of loss of OXPHOS on insulin signaling has been shown to be capricious even in controlled cell models (88). Instead, single-study observations of reduced muscle ‘OXPHOS’ gene expression arise because of a number of experimental covariates, including using ‘control’ muscle post ‘hyper-insulinemic clamp’—this acutely up-regulates OXPHOS pathway transcription in healthy subjects (i.e. the ‘control’ samples); or when subjects are not matched for aerobic capacity, physical activity, age or any combination of the above (24,87,118,119). In fact, even in skeletal muscle, the IS signature involves a *selective* alteration in AA, and lipid and ketone metabolizing genes (Table 2) in the complete absence of any loss of OXPHOS gene expression (*n* = 564, Figure 3). This indicates *qualitative* changes in substrate metabolism, rather than a crude loss of global oxidative capacity potentially underpins the correlative relationship between circulating BCAA and diabetes (47,120), and this would be teleologically consistent with the high absolute oxidative capacity found in muscle (purposed for contraction, and not representing a limit to substrate oxidation rates in resting muscle).

There is evidence that males and females with T2DM suffer different clinical sequela, particularly with respect to cardiovascular disease (121). The CORE-IS was not enriched in genes on the X or Y chromosomes; nor did it contain genes known to be subject to gender-specific imprinting. We examined the impact of sexual dimorphism on muscle gene expression, as this has not been reliably assessed before, and found 650 genes with sexual dimorphic gene expression (1%FDR, *n* = 89 per sex, Supplemental data S9) and this included 15% of the CORE-IS genes. Interestingly, the expression of a type II muscle fiber-type splice-variant (from the RYR1 gene) was lower expressed in women (Supplemental data S9); however, this prototype muscle fiber-type biomarker (43) did not correlate with IS. Thus, with our global transcriptomic methods, a modest relationship between sex, IS and muscle gene expression is detectable; potentially reflecting small differences in fiber-type composition, along with environmental factors, and this may contribute to differences in T2DM risk and disease progression between the genders (122). Far larger intervention studies, including many hundreds of both sexes, matched for physiological status, are required to robustly analyze if any variation in the responses to treatment reflects sexual dimorphism influenced genes, and thus this was not attempted in the present study.

### Non-coding transcriptome generates novel insights into IS

Using RNA-seq, the Illumina Human BodyMap and Genotype-Tissue Expression Project (GTEx, (30)) has reported a median count of less than 5000 lncRNA genes across a large number of human tissue and cell types (123), often with only a few hundred per tissue-type. The present analysis, which relies on a probe-level signal filter (study specificity), a secondary transcript-level ‘noise’ filter and confirmation that the low-end of the dynamic expression range equates to known low-expressed RNA and proteins (Supplemental data S10 and raw data), demonstrates the advantages of our new approach to customising array processing to tissue/study specific patterns of RNA detection. Further, based again on RNA-seq observations, there is a belief that lncRNAs are expressed in a highly cell-type specific manner. For example, the lncRNA antisense for *PDX1* (*PDX1-AS* or ’*PLUTO*’) - promotes expression of *PDX1* in in ß-cells via regulation of chromatin - is down-regulated in T2DM (45). While reported as ‘ß-cell specific’, we observed *PLUTO* to be expressed in skeletal muscle at a value equal to islet cell-line HTA 2.0 data (EndoC-ßH1 cells), while *PDX1* is 8-fold lower expressed in muscle than in islets (Supplemental data S10). Thus, while there are clearly further methodological factors to consider before we have a definitive view on the human transcriptome, the present analysis indicates that current representations of the ncRNA transcriptome by the Illumina Human BodyMap and GTEx are far from comprehensive, with reported tissue specific expression patterns being inaccurate. Notably, the IS-related ncRNAs in the present study are equally expressed in muscle, islet cells and adipose tissue (Figure 6 and Supplemental data S3) implying that they have the potential to impact on multiple aspects of insulin biology.

Akerman *et al* recently reported that modulation of lncRNA in islet cells impacts genes expression both in *cis* and *trans*, and impacts insulin production and secretion *in vitro* (45). Further, six ncRNAs were dysregulated in islet samples from people with impaired glucose tolerance and T2DM. Of these ncRNAs, we could only locate AC009487.1 in the current reference genome, and this gene was not detected in skeletal muscle. We did find *in vivo* regulation of ncRNAs in relation to peripheral IS before and after treatment aimed at improving IS (Figure 2; Supplemental data S3, S5, and S8). Strikingly, eleven ncRNAs were regulated by treatment and some directly in proportion to IS improvements; these included *PRKCQ-AS1*, which is anti-sense to the gene coding for PKC-θ, a known regulator of insulin signaling and also a drug-target for T2DM (83–85). We were one of the first groups to report *in vivo* interactions between lncRNA and a cis-expressed protein coding gene in humans (124). In the present study, we found that expression of *PRKCQ-AS1* and the mRNA for PKC-θ (*PRKCQ*) co-varied *in vivo* (Figure 2)—although the slope of the relationship indicates a modest interaction. *In vitro* loss of *PRKCQ-AS1* was associated with greater expression of *PRKCQ* (Figure 2D, (*P* = 0.05, *n* = 12 transfections). This suggests that PRKCQ-AS1 may act to maintain a relatively constant level of PRKCQ, despite fluctuations in transcription from that locus. Like other sense-antisense partners, e.g. *PDX1/PDX1-AS*, co-expression can also be reflective of regional chromatin status and hence transcription from that genomic locus (45). A ncRNA antisense transcript at the Sorbin and SH3 domain containing three (*SORBS3*) loci, *AC037459.2*, was also related to fasting IS. *SORBS3*—which codes for the protein vinexin—is down-regulated in the muscle of obese individuals, with a 5–25% increase in cytosine methylation at the *SORBS3* locus (125). Nonetheless, despite changes in expression of *ENTPD1-AS1, NPSR1-AS1* and *TTN-AS1* being positively associated with improvements in IS, and negatively associated for *ATP13A4-AS1* and *RORA-AS2*, transcripts at the *SORBS3* locus were stable to clinical intervention in our analysis. Further, detailed studies are merited, especially with respect to the complex *PRKCQ-AS1/PRKCQ* locus, given the importance of PKC-θ for insulin biology (84).

### Quantitative measures of RNA and interpretation of metabolic disease GWAS loci

Notably, when we compared the CORE-IS list with a recently published study that appraised *extreme* IR in a small group of obese individuals there was 100% concordance for directionality for the genes in common to both studies (Supplemental data S11, (126)). This would support the interpretation that the present CORE-IS signature represents the natural trajectory of obesity-related metabolic disease and, due to the continuous/quantitative nature of RNA data, we believe that the CORE-IS signature is potentially suitable for use as a drug-screen aimed at finding treatments for improving IS. So far, the majority of genome-wide association markers related to human disease do not occur within the sequence coding the processed transcript, but rather occur at regulatory regions potentially impacting on transcription, as well as RNA stability and translation (127). There has been limited progress moving from GWAS loci candidate regions to the identification of the potential gene responsible for the clinical associations, partly reflective of extensive linkage disequilibrium (14). Thus, identification of 45 GWAS-MD genome-wide significant loci (plus an additional nine GWAS significant from the network-analysis and >150 statistically weaker associations) indicates that network modeling of RNA (77) provides an opportunity to examine the continuous quantitative relationship between a genomic disease risk loci and the phenotype in question, something that is not possible with DNA sequence alone. It is important to state that there were no occasions when a CORE-IS gene only occurred at *non*-GWAS-MD gene using the Type 2 Diabetes knowledge portal (it contains GWAS results beyond metabolic disease). Further, in ∼5% of occasions GWAS-MD significant CORE-IS genes were associated with a non-metabolic disease at the ‘GWAS’ or a ‘suggestive association’ level, (but in most cases this non-metabolic phenotype was ‘Height’, which is related to body size and BMI calculation).

Gibson *et al* has demonstrated that global eQTL analysis can be an effective strategy for identifying the functional gene at GWAS loci for individual diseases (128). Indeed, large scale eQTL analysis of adipose tissue samples provides support for 14 of the GWAS-MD loci mentioned above, of which 7 increased the risk for T2DM. Genotype-transcript associations have also been observed in human pancreas islets, using cis-exon-eQTL mapping (129); where ∼2000 samples were required to identify an eQTL capturing 1% heritability (19). Van de Bunt *et al*. reported 2341 (or 6% of RNA-seq detectable genes) with an exon-eQTL within 250 kb of the transcription start site (an arbitrary distance) and 44 of these eQTL genes were related to peripheral IS in our analyses (Supplemental data S12). Further exploration of the CORE-IS gene-list using network analysis (77) in hepatic and adipose tissue would no doubt reveal additional commonalities across tissues; however, this will require new RNA datasets using RNA quantification methods consistent with the present study, which should overcome limitations in short-read RNA-seq (30,37), thus potentially circumventing some of the challenges faced when implementing eQTL methodologies (19).

### Network structures and dynamics responses of the CORE-IS genes

Using scale-free planar filtered network analysis (Table 3), we mapped the network structure of the CORE-IS genes (77). This approach—well suited to RNA profiles from a relatively cell-type homogenous tissue like skeletal muscle—reveals the regulatory interactions underlying insulin sensitivity as well as smaller functional sub-clusters of genes (modules). For example, we found *GRB14* closely co-expressed with six additional genes, including an aromatic AA transporter—*SLC16A10* (TAT1, Supplemental Figure S5). Using transcription factor analysis (63), we noted that four of these genes (*GRB14, HOMER1, KPNA5* and *SLC16A10*) had HNF1A type binding sites (*Z*-score = 9.3); *HNF1A* occurs at a GWAS risk loci for T2DM (71) and loss-of-function of HNF1A is related to familial early-onset diabetes (130). HNF1A type transcription factor binding sites were also enriched in CORE-IS genes differentially expressed following exercise-training (*Z*-score = 15.3) and interestingly polymorphisms in *HNF1A* are also linked to muscle histological phenotype (131), while this transcription factor also plays an established role in the pancreas. This illustrates the potentially pervasive influence of disease risk genes across multiple organs and the layers of genomic variants which influence the regulation of the expression of the CORE-IS genes.

Unique to the present analysis, we were able to identify a molecular signature that is regulated in proportion to the success of clinical interventions aimed at combating T2DM. A subset of 16 of the CORE-IS genes *quantitatively* related to improvement in insulin action in four diverse clinical studies, providing a major step toward identifying a molecular basis for the variable improvements in IS observed following standardized exercise/life-style interventions (48,50). One quarter were related to amino-acid metabolism (*ALDH6A1, DHTKD1, LAT3* and *MCCC1*) and were positively linked to IS; four genes were ‘substrate’ metabolism genes (*AGL, G0S2, KPNA2* and *PGM2*) involved in either blocking substrate mobilization or up-regulating glycolytic capacity, all negatively linked to IS and four were located at GWAS significant loci. *PRRX1* is involved in pancreatic developmental and modulating adipogenesis via TGFβ activity (132), a key pathway involved in the positive cardiovascular adaptation to endurance exercise (20,133), was positively associated with IS responses. This 16-gene signature represents a potentially useful insulin-target tissue biomarker for examining the responses to novel treatments aimed at improving IS.

In many of the largest GWAS studies, variation in fasting insulin was related to BMI with a shared variance of ∼33% (86) and statistical adjustment for BMI facilitated GWAS analysis. Recent studies have linked (implied causality) risk phenotypes for IR (e.g. elevated BMI, fasting glucose, fasting triglycerides (TG) or reduced HDL (69)) with GWAS significant loci. This has led to the emerging concept of failure in ‘adipose tissue capacity’ to adapt to over-feeding, promoting ectopic muscle and liver IR (69). Lotta *et al* combined information from 53 loci associated with higher fasting insulin, as well as lipids; a 53-SNP genetic score which associated with poor insulin sensitivity in >7000 independent subjects and a 12% increased relative risk for T2DM (69) and a lower rate of peripheral adipose tissue ‘accumulation’. This genetic estimate of IR status, was related to higher concentrations of all 3 BCAA’s (134) and included 5 CORE-IS genes from the present study (*GRB14, MCC, LPL, PPP1R3B* and *INSR*). A gain of function mutation in the lipid ‘mobilizing’ gene, *LPL*, is associated with better IS and protection from T2DM (69). Consistent with this, muscle LPL mRNA expression was positively associated with IS (*n* = 564, *R* = 0.35, FDR < 1%). It is understood that under normal physiological conditions in humans, a high proportion of body mass is attributable to skeletal muscle (∼40%), such that the same risk genes may relate to IS in both adipose and muscle tissue compartments, incorporating aspects of tissue plasticity that are common to adipose and muscle (20,135). Development and application of an appropriately validated multi-RNA risk-score will capture more clinical variance, and hence offers greater potential than the aforementioned 53-SNP genetic score.

In conclusion, based on the production and data modeling of a total of 1012 human muscle RNA profiles, as well as a new tissue-specific approach to substantially enhance the analysis of high-density DNA arrays, we produced a robust and highly validated global molecular model of human peripheral insulin sensitivity. The model revealed a novel interpretative structure for potentially exploring the dynamic interactions of metabolic-disease GWAS loci and, for the first time, illustrates the network interactions between the coding and non-coding IS transcriptome. Among other implications, this work lays the foundation for the discovery of novel biomarkers for metabolic disease and a tool for *in vitro* drug-screening.

## DATA AVAILABILITY

All new array data has been deposited at GEO (GSE104235).

## Supplementary Material

gky570_Supplemental_Files

## References

[1] Mercer T.R., Gerhardt D.J., Dinger M.E., Crawford J., Trapnell C., Jeddeloh J.A., Mattick J.S., Rinn J.L. Targeted RNA sequencing reveals the deep complexity of the human transcriptome. Nat. Biotechnol. 2012; 30:99–104. 10.1038/nbt.2024 PMC3710462 22081020

[2] Sood S., Gallagher I.J., Lunnon K., Rullman E., Keohane A., Crossland H., Phillips B.E., Cederholm T., Jensen T., van Loon L.J.C. et al. A novel multi-tissue RNA diagnostic of healthy ageing relates to cognitive health status. Genome Biol. 2015; 16:185. 26343147 10.1186/s13059-015-0750-x PMC4561473

[3] Wahlestedt C. Targeting long non-coding RNA to therapeutically upregulate gene expression. Nat. Rev. Drug Discov. 2013; 12:433–446. 23722346 10.1038/nrd4018

[4] Cohain A., Divaraniya A.A., Zhu K., Scarpa J.R., Kasarskis A., Zhu J., Chang R., Dudley J.T., Schadt E.E. Exploring the reproducibility of probabilistic causal molecular network models. 2017; 120–131.In *Biocomputing 2017*. 10.1142/9789813207813_0013 PMC5161348 27896968

[5] Zhang B., Gaiteri C., Bodea L.-G.G., Wang Z., McElwee J., Podtelezhnikov A.A., Zhang C., Xie T., Tran L., Dobrin R. et al. Integrated systems approach identifies genetic nodes and networks in late-onset Alzheimer's disease. Cell. 2013; 153:707–720. 23622250 10.1016/j.cell.2013.03.030 PMC3677161

[6] Chen Y., Zhu J., Lum P.Y., Yang X., Pinto S., MacNeil D.J., Zhang C., Lamb J., Edwards S., Sieberts S.K. et al. Variations in DNA elucidate molecular networks that cause disease. Nature. 2008; 452:429–435. 18344982 10.1038/nature06757 PMC2841398

[7] Shungin D., Winkler T.W., Croteau-Chonka D.C., Ferreira T., Locke A.E., Mägi R., Strawbridge R.J., Pers T.H., Fischer K., Justice A.E. et al. New genetic loci link adipose and insulin biology to body fat distribution. Nature. 2015; 518:187–196. 25673412 10.1038/nature14132 PMC4338562

[8] DeFronzo R.A., Tripathy D. Skeletal muscle insulin resistance is the primary defect in type 2 diabetes. Diabetes Care. 2009; 32(Suppl. 2):S157–S163. 19875544 10.2337/dc09-S302 PMC2811436

[9] Bornfeldt K.E., Tabas I. Insulin resistance, hyperglycemia, and atherosclerosis. Cell Metab. 2011; 14:575–585. 22055501 10.1016/j.cmet.2011.07.015 PMC3217209

[10] Wallace T.M., Levy J.C., Matthews D.R., Homa T. Use and abuse of HOMA modeling. Diabetes Care. 2004; 27:1487–1495. 15161807 10.2337/diacare.27.6.1487

[11] Matthews D.R., Hosker J.P., Rudenski A.S., Naylor B.A., Treacher D.F., Turner R.C. Homeostasis model assessment: insulin resistance and beta-cell function from fasting plasma glucose and insulin concentrations in man. Diabetologia. 1985; 28:412–419. 3899825 10.1007/BF00280883

[12] Mohlke K.L., Boehnke M. Recent advances in understanding the genetic architecture of type 2 diabetes. Hum. Mol. Genet. 2015; 24:R85–R92. 26160912 10.1093/hmg/ddv264 PMC4572004

[13] Flannick J., Florez J.C. Type 2 diabetes: genetic data sharing to advance complex disease research. Nat. Rev. Genet. 2016; 17:535–549. 27402621 10.1038/nrg.2016.56

[14] Visscher P.M., Wray N.R., Zhang Q., Sklar P., McCarthy M.I., Brown M.A., Yang J. 10 Years of GWAS discovery: biology, function, and translation. Am. J. Hum. Genet. 2017; 101:5–22. 28686856 10.1016/j.ajhg.2017.06.005 PMC5501872

[15] Zhu Z., Zhang F., Hu H., Bakshi A., Robinson M.R., Powell J.E., Montgomery G.W., Goddard M.E., Wray N.R., Visscher P.M. et al. Integration of summary data from GWAS and eQTL studies predicts complex trait gene targets. Nat. Genet. 2016; 48:481–487. 27019110 10.1038/ng.3538

[16] Scott R.A., Scott L.J., Mägi R., Marullo L., Gaulton K.J., Kaakinen M., Pervjakova N., Pers T.H., Johnson A.D., Eicher J.D. et al. An expanded genome-wide association study of type 2 diabetes in Europeans. Diabetes. 2017; 66:db161253. 10.2337/db16-1253 PMC5652602 28566273

[17] Jenkinson C.P., Göring H.H.H., Arya R., Blangero J., Duggirala R., DeFronzo R.A. Transcriptomics in type 2 diabetes: bridging the gap between genotype and phenotype. Genomics Data. 2016; 8:25–36. 27114903 10.1016/j.gdata.2015.12.001 PMC4832048

[18] Keildson S., Fadista J., Ladenvall C., Hedman A.K., Elgzyri T., Small K.S., Grundberg E., Nica A.C., Glass D., Richards J.B. et al. Expression of phosphofructokinase in skeletal muscle is influenced by genetic variation and associated with insulin sensitivity. Diabetes. 2014; 63:1154–1165. 24306210 10.2337/db13-1301 PMC3931395

[19] Hormozdiari F., van de Bunt M., Segrè A.V., Li X., Joo J.W.J., Bilow M., Sul J.H., Sankararaman S., Pasaniuc B., Eskin E. Colocalization of GWAS and eQTL signals detects target genes. Am. J. Hum. Genet. 2016; 99:1245–1260. 27866706 10.1016/j.ajhg.2016.10.003 PMC5142122

[20] Keller P., Vollaard N.B.J., Gustafsson T., Gallagher I.J., Sundberg C.J., Rankinen T., Britton S.L., Bouchard C., Koch L.G., Timmons J.A. A transcriptional map of the impact of endurance exercise training on skeletal muscle phenotype. J. Appl. Physiol. 2011; 110:46–59. 20930125 10.1152/japplphysiol.00634.2010 PMC3253010

[21] Parikh H., Carlsson E., Chutkow W.A., Johansson L.E., Storgaard H., Poulsen P., Saxena R., Ladd C., Schulze P.C., Mazzini M.J. et al. TXNIP regulates peripheral glucose metabolism in humans. PLoS Med. 2007; 4:e158. 17472435 10.1371/journal.pmed.0040158 PMC1858708

[22] Gallagher I.J., Scheele C., Keller P., Nielsen A.R., Remenyi J., Fischer C.P., Roder K., Babraj J., Wahlestedt C., Hutvagner G. et al. Integration of microRNA changes in vivo identifies novel molecular features of muscle insulin resistance in type 2 diabetes. Genome Med. 2010; 2:9. 20353613 10.1186/gm130 PMC2847700

[23] Jin W., Goldfine A.B., Boes T., Henry R.R., Ciaraldi T.P., Kim E.-Y., Emecan M., Fitzpatrick C., Sen A., Shah A. et al. Increased SRF transcriptional activity in human and mouse skeletal muscle is a signature of insulin resistance. J. Clin. Invest. 2011; 121:918–929. 21393865 10.1172/JCI41940 PMC3049368

[24] Väremo L., Scheele C., Broholm C., Mardinoglu A., Kampf C., Asplund A., Nookaew I., Uhlén M., Pedersen B.K., Nielsen J. Proteome- and transcriptome-driven reconstruction of the human myocyte metabolic network and its use for identification of markers for diabetes. Cell Rep. 2015; 11:921–933. 25937284 10.1016/j.celrep.2015.04.010

[25] Deveson I.W., Hardwick S.A., Mercer T.R., Mattick J.S. The dimensions, dynamics, and relevance of the mammalian noncoding transcriptome. Trends Genet. 2017; 33:464–478. 28535931 10.1016/j.tig.2017.04.004

[26] Mootha V.K., Lindgren C.M., Eriksson K.-F.F., Subramanian A., Sihag S., Lehar J., Puigserver P., Carlsson E., Ridderstråle M., Laurila E. et al. PGC-1alpha-responsive genes involved in oxidative phosphorylation are coordinately downregulated in human diabetes. Nat. Genet. 2003; 34:267–273. 12808457 10.1038/ng1180

[27] Xu W., Seok J., Mindrinos M.N., Schweitzer A.C., Jiang H., Wilhelmy J., Clark T.A., Kapur K., Xing Y., Faham M. et al. Human transcriptome array for high-throughput clinical studies. Proc. Natl. Acad. Sci. U.S.A. 2011; 108:3707–3712. 21317363 10.1073/pnas.1019753108 PMC3048146

[28] Larsson O., Tian B., Sonenberg N. Toward a genome-wide landscape of translational control. Cold Spring Harb. Perspect. Biol. 2013; 5:a012302. 23209130 10.1101/cshperspect.a012302 PMC3579401

[29] Scott L.J., Erdos M.R., Huyghe J.R., Welch R.P., Beck A.T., Wolford B.N., Chines P.S., Didion J.P., Narisu N., Stringham H.M. et al. The genetic regulatory signature of type 2 diabetes in in human skeletal muscle. Nat. Commun. 2016; 7:11764. 27353450 10.1038/ncomms11764 PMC4931250

[30] Mele M., Ferreira P.G., Reverter F., DeLuca D.S., Monlong J., Sammeth M., Young T.R., Goldmann J.M., Pervouchine D.D., Sullivan T.J. et al. The human transcriptome across tissues and individuals. Science. 2015; 348:660–665. 25954002 10.1126/science.aaa0355 PMC4547472

[31] Lindholm M.E., Huss M., Solnestam B.W., Kjellqvist S., Lundeberg J., Sundberg C.J. The human skeletal muscle transcriptome: sex differences, alternative splicing, and tissue homogeneity assessed with RNA sequencing. FASEB J. 2014; 28:4571–4581. 25016029 10.1096/fj.14-255000

[32] Lei R., Ye K., Gu Z., Sun X. Diminishing returns in next-generation sequencing (NGS) transcriptome data. Gene. 2015; 557:82–87. 25497830 10.1016/j.gene.2014.12.013

[33] Timmons J.A., Szkop K.J., Gallagher I.J. Multiple sources of bias confound functional enrichment analysis of global -omics data. Genome Biol. 2015; 16:186. 26346307 10.1186/s13059-015-0761-7 PMC4561415

[34] Jaffe A.E., Shin J., Collado-Torres L., Leek J.T., Tao R., Li C., Gao Y., Jia Y., Maher B.J., Hyde T.M. et al. Developmental regulation of human cortex transcription and its clinical relevance at single base resolution. Nat. Neurosci. 2014; 18:154–161. 25501035 10.1038/nn.3898 PMC4281298

[35] Miller J.A., Guillozet-Bongaarts A., Gibbons L.E., Postupna N., Renz A., Beller A.E., Sunkin S.M., Ng L., Rose S.E., Smith K.A. et al. Neuropathological and transcriptomic characteristics of the aged brain. Elife. 2017; 6:1–26. 10.7554/eLife.31126 PMC5679757 29120328

[36] Arner P., Sahlqvist A.S., Sinha I., Xu H., Yao X., Waterworth D., Rajpal D., Loomis A.K., Freudenberg J.M., Johnson T. et al. The epigenetic signature of systemic insulin resistance in obese women. Diabetologia. 2016; 59:2393–2405. 27535281 10.1007/s00125-016-4074-5 PMC5506095

[37] Sood S., Szkop K.J., Nakhuda A., Gallagher I.J., Murie C., Brogan R.J., Kaprio J., Kainulainen H., Atherton P.J., Kujala U.M. et al. iGEMS: An integrated model for identification of alternative exon usage events. Nucleic Acids Res. 2016; 44:1–14. 27095197 10.1093/nar/gkw263 PMC4914109

[38] Böhm A., Hoffmann C., Irmler M., Schneeweiss P., Schnauder G., Sailer C., Schmid V., Hudemann J., Machann J., Schick F. et al. TGF-β contributes to impaired exercise response by suppression of mitochondrial key regulators in skeletal muscle. Diabetes. 2016; 65:2849–2861. 27358493 10.2337/db15-1723

[39] Dai M., Wang P., Boyd A.D., Kostov G., Athey B., Jones E.G., Bunney W.E., Myers R.M., Speed T.P., Akil H. et al. Evolving gene/transcript definitions significantly alter the interpretation of GeneChip data. Nucleic Acids Res. 2005; 33:e175. 16284200 10.1093/nar/gni179 PMC1283542

[40] Langmead B., Salzberg S.L. Fast gapped-read alignment with Bowtie 2. Nat. Methods. 2012; 9:357–359. 22388286 10.1038/nmeth.1923 PMC3322381

[41] Bengtsson H., Simpson K., Bullard J., Hansen K. aroma.affymetrix: a generic framework in R for analyzing small to very large Affymetrix data sets in bounded memory. Dep. Stat. Univ. California, Berkeley. 2008; 745:1–9.

[42] Gentleman R.C., Carey V.J., Bates D.M., Bolstad B., Dettling M., Dudoit S., Ellis B., Gautier L., Ge Y., Gentry J. et al. Bioconductor: open software development for computational biology and bioinformatics. Genome Biol. 2004; 5:R80. 15461798 10.1186/gb-2004-5-10-r80 PMC545600

[43] Willemse H., Theodoratos A., Smith P.N., Dulhunty A.F. Unexpected dependence of RyR1 splice variant expression in human lower limb muscles on fiber-type composition. Pflügers Arch. - Eur. J. Physiol. 2016; 468:269–278. 26438192 10.1007/s00424-015-1738-9

[44] Bolstad B.M., Irizarry R.A., Astrand M., Speed T.P. A comparison of normalization methods for high density oligonucleotide array data based on variance and bias. Bioinformatics. 2003; 19:185–193. 12538238 10.1093/bioinformatics/19.2.185

[45] Akerman I., Tu Z., Beucher A., Rolando D.M.Y.Y., Sauty-Colace C., Benazra M., Nakic N., Yang J., Wang H., Pasquali L. et al. Human pancreatic beta-cell lncRNAs control cell-specific regulatory networks. Cell Metab. 2017; 25:400–411. 28041957 10.1016/j.cmet.2016.11.016 PMC5300904

[46] Nakhuda A., Josse A.R., Gburcik V., Crossland H., Raymond F., Metairon S., Good L., Atherton P.J., Phillips S.M., Timmons J.A. Biomarkers of browning of white adipose tissue and their regulation. Am. J. Clin. Nutr. 2016; 104:557–565. 27488235 10.3945/ajcn.116.132563 PMC4997298

[47] Glynn E.L., Piner L.W., Huffman K.M., Slentz C.A., Elliot-Penry L., AbouAssi H., White P.J., Bain J.R., Muehlbauer M.J., Ilkayeva O.R. et al. Impact of combined resistance and aerobic exercise training on branched-chain amino acid turnover, glycine metabolism and insulin sensitivity in overweight humans. Diabetologia. 2015; 58:2324–2335. 26254576 10.1007/s00125-015-3705-6 PMC4793723

[48] Slentz C.A., Bateman L.A., Willis L.H., Granville E.O., Piner L.W., Samsa G.P., Setji T.L., Muehlbauer M.J., Huffman K.M., Bales C.W. et al. Effects of exercise training alone vs a combined exercise and nutritional lifestyle intervention on glucose homeostasis in prediabetic individuals: a randomised controlled trial. Diabetologia. 2016; 59:2088–2098. 27421729 10.1007/s00125-016-4051-z PMC5026926

[49] Tieland M., Dirks M.L., van der Zwaluw N., Verdijk L.B., van de Rest O., de Groot L.C.P.G.M., van Loon L.J.C. Protein supplementation increases muscle mass gain during prolonged resistance-type exercise training in frail elderly people: a randomized, double-blind, placebo-controlled trial. J. Am. Med. Dir. Assoc. 2012; 13:713–719. 22770932 10.1016/j.jamda.2012.05.020

[50] Phillips B., Kelly B., Lilja M., Ponce-González J., Brogan R., Morris D., Gustafsson T., Kraus W., Atherton P., Vollaard N. et al. A practical and time-efficient high-intensity interval training programme modifies cardio-metabolic risk-factors in adults with risk-factors for Type II diabetes. Front. Endocrinol. (Lausanne). 2017; 8:229. 28943861 10.3389/fendo.2017.00229 PMC5596071

[51] Hangelbroek R.W.J., Fazelzadeh P., Tieland M., Boekschoten M.V., Hooiveld G.J.E., van Duynhoven J.P.M., Timmons J.A., Verdijk L.B., de Groot L.C.P., van Loon L.J.C. et al. Expression of protocadherin gamma in skeletal muscle tissue is associated with age and muscle weakness. J. Cachexia. Sarcopenia Muscle. 2016; 7:604–614. 27239416 10.1002/jcsm.12099 PMC4863830

[52] Josse A.R., Atkinson S.A., Tarnopolsky M.A., Phillips S.M. Increased consumption of dairy foods and protein during diet- and exercise-induced weight loss promotes fat mass loss and lean mass gain in overweight and obese premenopausal women. J. Nutr. 2011; 141:1626–1634. 21775530 10.3945/jn.111.141028 PMC3159052

[53] Phillips B.E., Williams J.P., Gustafsson T., Bouchard C., Rankinen T., Knudsen S., Smith K., Timmons J.A., Atherton P.J. Molecular networks of human muscle adaptation to exercise and age. PLoS Genet. 2013; 9:e1003389. 23555298 10.1371/journal.pgen.1003389 PMC3605101

[54] Barberio M.D., Huffman K.M., Giri M., Hoffman E.P., Kraus W.E., Hubal M.J. Pyruvate dehydrogenase phosphatase regulatory gene expression correlates with exercise training insulin sensitivity changes. Med. Sci. Sports Exerc. 2016; 48:2387–2397. 27846149 10.1249/MSS.0000000000001041 PMC5141615

[55] AbouAssi H., Slentz C.A., Mikus C.R., Tanner C.J., Bateman L.A., Willis L.H., Shields A.T., Piner L.W., Elliott-Penry L.E., Kraus E.A. et al. The effects of aerobic, resistance and combination training on insulin sensitivity and secretion in overweight adults from STRRIDE AT/RT: a randomized trial. J. Appl. Physiol. 2015; 118:1474–1482. 25882384 10.1152/japplphysiol.00509.2014 PMC4469920

[56] Sears D.D., Hsiao G., Hsiao A., Yu J.G., Courtney C.H., Ofrecio J.M., Chapman J., Subramaniam S. Mechanisms of human insulin resistance and thiazolidinedione-mediated insulin sensitization. Proc. Natl. Acad. Sci. U.S.A. 2009; 106:18745–18750. 19841271 10.1073/pnas.0903032106 PMC2763882

[57] Cotie L.M., Josse A.R., Phillips S.M., MacDonald M.J. Endothelial function increases after a 16-week diet and exercise intervention in overweight and obese young women. Biomed. Res. Int. 2014; 2014:327395. 24772421 10.1155/2014/327395 PMC3977448

[58] Slentz C.A., Aiken L.B., Houmard J.A., Bales C.W., Johnson J.L., Tanner C.J., Duscha B.D., Kraus W.E. Inactivity, exercise, and visceral fat. STRRIDE: a randomized, controlled study of exercise intensity and amount. J. Appl. Physiol. 2005; 99:1613–1618. 16002776 10.1152/japplphysiol.00124.2005

[59] Marcovina S., Bowsher R.R., Miller W.G., Staten M., Myers G., Caudill S.P., Campbell S.E., Steffes M.W. Standardization of insulin immunoassays: report of the American Diabetes Association Workgroup. Clin. Chem. 2007; 53:711–716. 17272483 10.1373/clinchem.2006.082214

[60] Wang X., Kang D.D., Shen K., Song C., Lu S., Chang L.C., Liao S.G., Huo Z., Tang S., Ding Y. et al. An r package suite for microarray meta-analysis in quality control, differentially expressed gene analysis and pathway enrichment detection. Bioinformatics. 2012; 28:2534–2536. 22863766 10.1093/bioinformatics/bts485 PMC3463115

[61] Smyth G.K. Gentleman R, Carey V, Dudoit S, Irizarry R, Huber W Limma: linear models for microarray data. Bioinformatics and Computational Biology Solutions using R and Bioconductor. 2005; NY: Springer 397–420.

[62] Timmons J.A., Gallagher I.J. Molecular studies of exercise, skeletal muscle, and ageing. F1000Research. 2016; 5:doi:10.12688/f1000research.8255.1. 10.12688/f1000research.8255.1 PMC4892337 27303646

[63] Sui HoS.J., Fulton D.L., Arenillas D.J., Kwon A.T., Wasserman W.W. OPOSSUM: integrated tools for analysis of regulatory motif over-representation. Nucleic Acids Res. 2007; 35:245–252. 10.1093/nar/gkm427 PMC1933229 17576675

[64] Bryne J.C., Valen E., Tang M.H., Marstrand T., Winther O., da Piedade I., Krogh A., Lenhard B., Sandelin A. JASPAR, the open access database of transcription factor-binding profiles: new content and tools in the 2008 update. Nucleic Acids Res. 2008; 36:D102–D106. 18006571 10.1093/nar/gkm955 PMC2238834

[65] Fuchsberger C., Flannick J., Teslovich T.M., Mahajan A., Agarwala V., Gaulton K.J., Ma C., Fontanillas P., Moutsianas L., McCarthy D.J. et al. The genetic architecture of type 2 diabetes. Nature. 2016; 536:41–47. 27398621 10.1038/nature18642 PMC5034897

[66] Willer C.J., Schmidt E.M., Sengupta S., Peloso G.M., Gustafsson S., Kanoni S., Ganna A., Chen J., Buchkovich M.L., Mora S. et al. Discovery and refinement of loci associated with lipid levels. Nat. Genet. 2013; 45:1274–1283. 24097068 10.1038/ng.2797 PMC3838666

[67] Dupuis J.J., Langenberg C., Prokopenko I., Saxena R., Soranzo N., Jackson A.U., Wheeler E., Glazer N.L., Bouatia-Naji N., Gloyn A.L. et al. New genetic loci implicated in fasting glucose homeostasis and their impact on type 2 diabetes risk. Nat. Genet. 2010; 42:105–116. 20081858 10.1038/ng.520 PMC3018764

[68] Voight B.F., Scott L.J., Steinthorsdottir V., Morris A.D.P., Dina C., Welch R.P., Zeggini E., Huth C., Aulchenko Y.S., Thorleifsson G. et al. Twelve type 2 diabetes susceptibility loci identified through large-scale association analysis. Nat. Genet. 2010; 42:579–589. 20581827 10.1038/ng.609 PMC3080658

[69] Lotta L.A., Gulati P., Day F.R., Payne F., Ongen H., van de Bunt M., Gaulton K.J., Eicher J.D., Sharp S.J., Luan J. et al. Integrative genomic analysis implicates limited peripheral adipose storage capacity in the pathogenesis of human insulin resistance. Nat. Genet. 2016; 49:17–26. 27841877 10.1038/ng.3714 PMC5774584

[70] Locke A.E., Kahali B., Berndt S.I., Justice A.E., Pers T.H., Day F.R., Powell C., Vedantam S., Buchkovich M.L., Yang J. et al. Genetic studies of body mass index yield new insights for obesity biology. Nature. 2015; 518:197–206. 25673413 10.1038/nature14177 PMC4382211

[71] Scott R.A., Fall T., Pasko D., Barker A., Sharp S.J., Arriola L., Balkau B., Barricarte A., Barroso I., Boeing H. et al. Common genetic variants highlight the role of insulin resistance and body fat distribution in type 2 diabetes, independent of obesity. Diabetes. 2014; 63:4378–4387. 24947364 10.2337/db14-0319 PMC4241116

[72] Gaulton K.J., Ferreira T., Lee Y., Raimondo A., Mägi R., Reschen M.E., Mahajan A., Locke A., Rayner N.W., Robertson N. et al. Genetic fine mapping and genomic annotation defines causal mechanisms at type 2 diabetes susceptibility loci. Nat. Genet. 2015; 47:1415–1425. 26551672 10.1038/ng.3437 PMC4666734

[73] Manning A.K., Hivert M.-F., Scott R.A., Grimsby J.L., Bouatia-Naji N., Chen H., Rybin D., Liu C.-T., Bielak L.F., Prokopenko I. et al. A genome-wide approach accounting for body mass index identifies genetic variants influencing fasting glycemic traits and insulin resistance. Nat. Genet. 2012; 44:659–669. 22581228 10.1038/ng.2274 PMC3613127

[74] Horikoshi M., Mägi R., van de Bunt M., Surakka I., Sarin A.P., Mahajan A., Marullo L., Thorleifsson G., Hägg S., Hottenga J.J. et al. Discovery and fine-mapping of glycaemic and obesity-related trait loci using high-density imputation. PLoS Genet. 2015; 11:1–24. 10.1371/journal.pgen.1005230 PMC4488845 26132169

[75] Zhao W., Rasheed A., Tikkanen E., Lee J.-J., Butterworth A.S., Howson J.M.M., Assimes T.L., Chowdhury R., Orho-Melander M., Damrauer S. et al. Identification of new susceptibility loci for type 2 diabetes and shared etiological pathways with coronary heart disease. Nat. Genet. 2017; 49:1450–1457. 28869590 10.1038/ng.3943 PMC5844224

[76] Liu D.J., Peloso G.M., Yu H., Butterworth A.S., Wang X., Mahajan A., Saleheen D., Emdin C., Alam D., Alves A.C. et al. Exome-wide association study of plasma lipids in >300,000 individuals. Nat. Genet. 2017; 49:1758–1766. 29083408 10.1038/ng.3977 PMC5709146

[77] Song W.M., Zhang B. Multiscale embedded gene Co-expression network analysis. PLoS Comput. Biol. 2015; 11:e1004574. 26618778 10.1371/journal.pcbi.1004574 PMC4664553

[78] Xia J., Gill E.E., Hancock R.E.W. NetworkAnalyst for statistical, visual and network-based meta-analysis of gene expression data. Nat. Protoc. 2015; 10:823–844. 25950236 10.1038/nprot.2015.052

[79] Breuer K., Foroushani A.K., Laird M.R., Chen C., Sribnaia A., Lo R., Winsor G.L., Hancock R.E.W., Brinkman F.S.L., Lynn D.J. InnateDB: systems biology of innate immunity and beyond–recent updates and continuing curation. Nucleic Acids Res. 2013; 41:D1228–D1233. 23180781 10.1093/nar/gks1147 PMC3531080

[80] Wolfe C.J., Kohane I.S., Butte A.J. Systematic survey reveals general applicability of “guilt-by-association” within gene coexpression networks. BMC Bioinformatics. 2005; 6:227. 16162296 10.1186/1471-2105-6-227 PMC1239911

[81] van Dam S., Craig T., de Magalhães J.P. GeneFriends: a human RNA-seq-based gene and transcript co-expression database. Nucleic Acids Res. 2015; 43:D1124–D1132. 25361971 10.1093/nar/gku1042 PMC4383890

[82] Condorelli G., Vigliotta G., Trencia A., Maitan M.A., Caruso M., Miele C., Oriente F., Santopietro S., Formisano P., Beguinot F. Protein kinase C (PKC)-alpha activation inhibits PKC-zeta and mediates the action of PED/PEA-15 on glucose transport in the L6 skeletal muscle cells. Diabetes. 2001; 50:1244–1252. 11375323 10.2337/diabetes.50.6.1244

[83] Hegarty B.D., Furler S.M., Ye J., Cooney G.J., Kraegen E.W. The role of intramuscular lipid in insulin resistance. Acta Physiol. Scand. 2003; 178:373–383. 12864742 10.1046/j.1365-201X.2003.01162.x

[84] Szendroedi J., Yoshimura T., Phielix E., Koliaki C., Marcucci M., Zhang D., Jelenik T., Müller J., Herder C., Nowotny P. et al. Role of diacylglycerol activation of PKCθ in lipid-induced muscle insulin resistance in humans. Proc. Natl. Acad. Sci. U.S.A. 2014; 111:9597–9602. 24979806 10.1073/pnas.1409229111 PMC4084449

[85] Capel F., Acquaviva C., Pitois E., Laillet B., Rigaudière J.-P., Jouve C., Pouyet C., Gladine C., Comte B., Vianey Saban C. et al. DHA at nutritional doses restores insulin sensitivity in skeletal muscle by preventing lipotoxicity and inflammation. J. Nutr. Biochem. 2015; 26:949–959. 26007287 10.1016/j.jnutbio.2015.04.003

[86] Scott R.A., Lagou V., Welch R.P., Wheeler E., Montasser M.E., Luan J., Mägi R., Strawbridge R.J., Rehnberg E., Gustafsson S. et al. Large-scale association analyses identify new loci influencing glycemic traits and provide insight into the underlying biological pathways. Nat. Genet. 2012; 44:991–1005. 22885924 10.1038/ng.2385 PMC3433394

[87] Patti M.E., Butte A.J., Crunkhorn S., Cusi K., Berria R., Kashyap S., Miyazaki Y., Kohane I., Costello M., Saccone R. et al. Coordinated reduction of genes of oxidative metabolism in humans with insulin resistance and diabetes: potential role of PGC1 and NRF1. Proc. Natl. Acad. Sci. U.S.A. 2003; 100:8466–8471. 12832613 10.1073/pnas.1032913100 PMC166252

[88] Martin S.D., Morrison S., Konstantopoulos N., McGee S.L. Mitochondrial dysfunction has divergent, cell type-dependent effects on insulin action. Mol. Metab. 2014; 3:408–418. 24944900 10.1016/j.molmet.2014.02.001 PMC4060359

[89] Knowler W.C., Fowler S.E., Hamman R.F., Christophi C.A., Hoffman H.J., Brenneman A.T., Brown-Friday J.O., Goldberg R., Venditti E., Nathan D.M. 10-year follow-up of diabetes incidence and weight loss in the Diabetes Prevention Program Outcomes Study. Lancet. 2009; 374:1677–1686. 19878986 10.1016/S0140-6736(09)61457-4 PMC3135022

[90] Felig P., Marliss E., Cahill G.F. Plasma Amino Acid Levels and Insulin Secretion in Obesity. N. Engl. J. Med. 1969; 281:811–816. 5809519 10.1056/NEJM196910092811503

[91] Jang C., Oh S.F., Wada S., Rowe G.C., Liu L., Chan M.C., Rhee J., Hoshino A., Kim B., Ibrahim A. et al. A branched-chain amino acid metabolite drives vascular fatty acid transport and causes insulin resistance. Nat. Med. 2016; 22:421–426. 26950361 10.1038/nm.4057 PMC4949205

[92] Cao Z., Xia Z., Zhou Y., Yang X., Hao H., Peng N., Liu S., Zhu Y. Methylcrotonoyl-CoA carboxylase 1 potentiates RLR-induced NF-κB signaling by targeting MAVS complex. Sci. Rep. 2016; 6:33557. 27629939 10.1038/srep33557 PMC5024325

[93] Sajuthi S.P., Sharma N.K., Chou J.W., Palmer N.D., McWilliams D.R., Beal J., Comeau M.E., Ma L., Calles-Escandon J., Demons J. et al. Mapping adipose and muscle tissue expression quantitative trait loci in African Americans to identify genes for type 2 diabetes and obesity. Hum. Genet. 2016; 135:869–880. 27193597 10.1007/s00439-016-1680-8 PMC4947558

[94] Pal D., Wu D., Haruta A., Matsumura F., Wei Q. Role of a novel coiled-coil domain-containing protein CCDC69 in regulating central spindle assembly. Cell Cycle. 2010; 9:4117–4129. 20962590 10.4161/cc.9.20.13387 PMC3055196

[95] Mayo L., Trauger S.A., Blain M., Nadeau M., Patel B., Alvarez J.I., Mascanfroni I.D., Yeste A., Kivisäkk P., Kallas K. et al. Regulation of astrocyte activation by glycolipids drives chronic CNS inflammation. Nat. Med. 2014; 20:1147–1156. 25216636 10.1038/nm.3681 PMC4255949

[96] Li S., Song W., Jiang M., Zeng L., Zhu X., Chen J. Phosphorylation of cyclin Y by CDK14 induces its ubiquitination and degradation. FEBS Lett. 2014; 588:1989–1996. 24794231 10.1016/j.febslet.2014.04.019

[97] Turpin S.M.M., Nicholls H.T.T., Willmes D.M.M., Mourier A., Brodesser S., Wunderlich C.M.M., Mauer J., Xu E., Hammerschmidt P., Brönneke H.S.S. et al. Obesity-induced CerS6-dependent C16:0 ceramide production promotes weight gain and glucose intolerance. Cell Metab. 2014; 20:678–686. 25295788 10.1016/j.cmet.2014.08.002

[98] Arif A., Terenzi F., Potdar A.A., Jia J., Sacks J., China A., Halawani D., Vasu K., Li X., Brown J.M. et al. EPRS is a critical mTORC1–S6K1 effector that influences adiposity in mice. Nature. 2017; 542:357–361. 28178239 10.1038/nature21380 PMC5480610

[99] Yuan H.-X., Wang Z., Yu F.-X., Li F., Russell R.C., Jewell J.L., Guan K.-L. NLK phosphorylates Raptor to mediate stress-induced mTORC1 inhibition. Genes Dev. 2015; 29:2362–2376. 26588989 10.1101/gad.265116.115 PMC4691891

[100] Singh R., De Aguiar R.B., Naik S., Mani S., Ostadsharif K., Wencker D., Sotoudeh M., Malekzadeh R., Sherwin R.S., Mani A. LRP6 enhances glucose metabolism by promoting TCF7L2-Dependent insulin receptor expression and IGF receptor stabilization in humans. Cell Metab. 2013; 17:197–209. 23395167 10.1016/j.cmet.2013.01.009 PMC3589523

[101] Desbuquois B., Carré N., Burnol A.-F.F. Regulation of insulin and type 1 insulin-like growth factor signaling and action by the Grb10/14 and SH2B1/B2 adaptor proteins. FEBS J. 2013; 280:794–816. 23190452 10.1111/febs.12080

[102] Budanov A.V. SESTRINs regulate mTORC1 via RRAGs: the riddle of GATOR. Mol. Cell. Oncol. 2015; 2:e997113. 27308486 10.1080/23723556.2014.997113 PMC4905323

[103] Taira J., Higashimoto Y. Phosphorylation of Grb14 BPS domain by GSK-3 correlates with complex forming of Grb14 and insulin receptor. J. Biochem. 2014; 155:353–360. 24535599 10.1093/jb/mvu011

[104] Park J.-J., Berggren J.R., Hulver M.W., Houmard J.A., Hoffman E.P. GRB14, GPD1, and GDF8 as potential network collaborators in weight loss-induced improvements in insulin action in human skeletal muscle. Physiol. Genomics. 2006; 27:114–121. 16849634 10.1152/physiolgenomics.00045.2006

[105] Popineau L., Morzyglod L., Carré N., Caüzac M., Bossard P., Prip-Buus C., Lenoir V., Ragazzon B., Fauveau V., Robert L. et al. A novel Grb14-mediated cross-talk between insulin and p62/Nrf2 pathways regulates liver lipogenesis and selective insulin resistance. Mol. Cell. Biol. 2016; 36:2168–2181. 27215388 10.1128/MCB.00170-16 PMC4968215

[106] Kelley D.E., Goodpaster B.H., Storlien L. Muscle triglyceride and insulin resistance. Annu. Rev. Nutr. 2002; 22:325–346. 12055349 10.1146/annurev.nutr.22.010402.102912

[107] Tuuf J., Kjellberg M.A., Molotkovsky J.G., Hanada K., Mattjus P. The intermembrane ceramide transport catalyzed by CERT is sensitive to the lipid environment. Biochim. Biophys. Acta - Biomembr. 2011; 1808:229–235. 10.1016/j.bbamem.2010.09.011 20875392

[108] Dube J.J., Amati F., Stefanovic-Racic M., Toledo F.G.S., Sauers S.E., Goodpaster B.H. Exercise-induced alterations in intramyocellular lipids and insulin resistance: the athlete's paradox revisited. AJP Endocrinol. Metab. 2008; 294:E882–E888. 10.1152/ajpendo.00769.2007 PMC3804891 18319352

[109] Holten M.K., Zacho M., Gaster M., Juel C., Wojtaszewski J.F., Dela F. Strength training increases insulin-mediated glucose uptake, GLUT4 content, and insulin signaling in skeletal muscle in patients with type 2 diabetes. Diabetes. 2004; 53:294–305. 14747278 10.2337/diabetes.53.2.294

[110] Houmard J.A., Tanner C.J., Slentz C.A., Duscha B.D., McCartney J.S., Kraus W.E. Effect of the volume and intensity of exercise training on insulin sensitivity. J. Appl. Physiol. 2004; 96:101–106. 12972442 10.1152/japplphysiol.00707.2003

[111] Krotkiewski M., Lönnroth P., Mandroukas K., Wroblewski Z., Rebuffé-Scrive M., Holm G., Smith U., Björntorp P., Bjorntorp P. The effects of physical training on insulin secretion and effectiveness and on glucose metabolism in obesity and Type 2 (non-insulin-dependent) diabetes mellitus. Diabetologia. 1985; 28:881–890. 3912243 10.1007/BF00703130

[112] Johnson J.L., Slentz C.A., Houmard J.A., Samsa G.P., Duscha B.D., Aiken L.B., McCartney J.S., Tanner C.J., Kraus W.E. Exercise training amount and intensity effects on metabolic syndrome (from Studies of a Targeted Risk Reduction Intervention through Defined Exercise). Am. J. Cardiol. 2007; 100:1759–1766. 18082522 10.1016/j.amjcard.2007.07.027 PMC2190779

[113] García-Palmero I., Pompas-Veganzones N., Villalobo E., Gioria S., Haiech J., Villalobo A. The adaptors Grb10 and Grb14 are calmodulin-binding proteins. FEBS Lett. 2017; 591:1176–1186. 28295264 10.1002/1873-3468.12623

[114] Taira J., Kida Y., Inatomi K., Komatsu H., Higashimoto Y., Sakamoto H. Phosphorylation of clustered serine residues in the N-terminus of BPS domain negatively regulates formation of the complex between human Grb14 and insulin receptor. J. Biochem. 2017; 162:113–122. 28130417 10.1093/jb/mvx007

[115] Jingyi B., Li J., Biggin M.D. Statistics requantitates the central dogma. Science. 2015; 347:2015–2017. 10.1126/science.aaa8332 25745146

[116] Knott C., Bell S., Britton A. Alcohol consumption and the risk of type 2 diabetes: a systematic review and dose-response meta-analysis of more than 1.9 million individuals from 38 observational studies. Diabetes Care. 2015; 38:1804–1812. 26294775 10.2337/dc15-0710

[117] Spiegelman B.M. Transcriptional control of mitochondrial energy metabolism through the PGC1 coactivators. Novartis Found. Symp. 2007; 287:60–69. 18074631

[118] Pihlajamäki J., Lerin C., Itkonen P., Boes T., Floss T., Schroeder J., Dearie F., Crunkhorn S., Burak F., Jimenez-Chillaron J.C. et al. Expression of the splicing factor gene SFRS10 is reduced in human obesity and contributes to enhanced lipogenesis. Cell Metab. 2011; 14:208–218. 21803291 10.1016/j.cmet.2011.06.007 PMC3167228

[119] Elbein S.C., Kern P.A., Rasouli N., Yao-Borengasser A., Sharma N.K., Das S.K. Global gene expression profiles of subcutaneous adipose and muscle from glucose-tolerant, insulin-sensitive, and insulin-resistant individuals matched for BMI. Diabetes. 2011; 60:1019–1029. 21266331 10.2337/db10-1270 PMC3046820

[120] Giesbertz P., Daniel H. Branched-chain amino acids as biomarkers in diabetes. Curr. Opin. Clin. Nutr. Metab. Care. 2015; 19:1. 10.1097/MCO.0000000000000235 26485337

[121] Kautzky-Willer A., Harreiter J., Pacini G. Sex and gender differences in risk, pathophysiology and complications of type 2 diabetes mellitus. Endocr. Rev. 2016; 37:278–316. 27159875 10.1210/er.2015-1137 PMC4890267

[122] Sattar N. Gender aspects in type 2 diabetes mellitus and cardiometabolic risk. Best Pract. Res. Clin. Endocrinol. Metab. 2013; 27:501–507. 24054927 10.1016/j.beem.2013.05.006

[123] Xu J., Bai J., Zhang X., Lv Y., Gong Y., Liu L., Zhao H., Yu F., Ping Y., Zhang G. et al. A comprehensive overview of lncRNA annotation resources. Brief. Bioinform. 2017; 18:236–249. 26944085 10.1093/bib/bbw015

[124] Scheele C., Petrovic N., Faghihi M.A., Lassmann T., Fredriksson K., Rooyackers O., Wahlestedt C., Good L., Timmons J.A. The human PINK1 locus is regulated in vivo by a non-coding natural antisense RNA during modulation of mitochondrial function. BMC Genomics. 2007; 8:74. 17362513 10.1186/1471-2164-8-74 PMC1831481

[125] Day S.E., Coletta R.L., Kim J.Y., Campbell L.E., Benjamin T.R., Roust L.R., De Filippis E.A., Dinu V., Shaibi G.Q., Mandarino L.J. et al. Next-generation sequencing methylation profiling of subjects with obesity identifies novel gene changes. Clin. Epigenetics. 2016; 8:77. 27437034 10.1186/s13148-016-0246-x PMC4950754

[126] Møller A.B., Kampmann U., Hedegaard J., Thorsen K., Nordentoft I., Vendelbo M.H., Møller N., Jessen N. Altered gene expression and repressed markers of autophagy in skeletal muscle of insulin resistant patients with type 2 diabetes. Sci. Rep. 2017; 7:43775. 28252104 10.1038/srep43775 PMC5333153

[127] Gibson G., Powell J.E., Marigorta U.M. Expression quantitative trait locus analysis for translational medicine. Genome Med. 2015; 7:60. 26110023 10.1186/s13073-015-0186-7 PMC4479075

[128] Marigorta U.M., Denson L.A., Hyams J.S., Mondal K., Prince J., Walters T.D., Griffiths A., Noe J.D., Crandall W. V, Rosh J.R. et al. Transcriptional risk scores link GWAS to eQTLs and predict complications in Crohn's disease. Nat. Genet. 2017; 49:1517–1521. 28805827 10.1038/ng.3936 PMC5745037

[129] van de Bunt M., Manning Fox J.E., Dai X., Barrett A., Grey C., Li L., Bennett A.J., Johnson P.R., Rajotte R.V., Gaulton K.J. et al. Transcript expression data from human islets links regulatory signals from Genome-Wide association studies for type 2 diabetes and glycemic traits to their downstream effectors. PLoS Genet. 2015; 11:e1005694. 26624892 10.1371/journal.pgen.1005694 PMC4666611

[130] Triggs-Raine B.L., Kirkpatrick R.D., Kelly S.L., Norquay L.D., Cattini P.A., Yamagata K., Hanley A.J.G., Zinman B., Harris S.B., Barrett P.H. et al. HNF-1alpha G319S, a transactivation-deficient mutant, is associated with altered dynamics of diabetes onset in an Oji-Cree community. Proc. Natl. Acad. Sci. U.S.A. 2002; 99:4614–4619. 11904371 10.1073/pnas.062059799 PMC123696

[131] Kayan A., Uddin M.J., Kocamis H., Tesfaye D., Looft C., Tholen E., Schellander K., Cinar M.U. Association and expression analysis of porcine HNF1A gene related to meat and carcass quality traits. Meat Sci. 2013; 94:474–479. 23628452 10.1016/j.meatsci.2013.04.015

[132] Du B., Cawthorn W.P., Su A., Doucette C.R., Yao Y., Hemati N., Kampert S., McCoin C., Broome D.T., Rosen C.J. et al. The transcription factor paired-related homeobox 1 (Prrx1) inhibits adipogenesis by activating transforming growth factor-β (TGFβ) signaling. J. Biol. Chem. 2013; 288:3036–3047. 23250756 10.1074/jbc.M112.440370 PMC3561528

[133] Lessard S.J., Rivas D.A., Alves-Wagner A.B., Hirshman M.F., Gallagher I.J., Constantin-Teodosiu D., Atkins R., Greenhaff P.L., Qi N.R., Gustafsson T. et al. Resistance to aerobic exercise training causes metabolic dysfunction and reveals novel exercise-regulated signaling networks. Diabetes. 2013; 62:2717–2727. 23610057 10.2337/db13-0062 PMC3717870

[134] Wang Q., Holmes M. V., Davey Smith G., Ala-Korpela M. Genetic support for a causal role of insulin resistance on circulating branched-chain amino acids and inflammation. Diabetes Care. 2017; 40:1779–1786. 29046328 10.2337/dc17-1642 PMC5701741

[135] Crewe C., An Y.A., Scherer P.E. The ominous triad of adipose tissue dysfunction: inflammation, fibrosis, and impaired angiogenesis. J. Clin. Invest. 2017; 127:74–82. 28045400 10.1172/JCI88883 PMC5199684

[136] McKenzie A.T., Katsyv I., Song W.-M., Wang M., Zhang B. DGCA: a comprehensive R package for Differential gene correlation analysis. BMC Syst. Biol. 2016; 10:106. 27846853 10.1186/s12918-016-0349-1 PMC5111277

[137] Xia J., Benner M.J., Hancock R.E.W. NetworkAnalyst - integrative approaches for protein–protein interaction network analysis and visual exploration. Nucleic Acids Res. 2014; 42:167–174. 10.1093/nar/gku443 PMC4086107 24861621

[138] van Dam S., Võsa U., van der Graaf A., Franke L., de Magalhães J.P. Gene co-expression analysis for functional classification and gene–disease predictions. Brief. Bioinform. 2017; doi:10.1093/bib/bbw139. 10.1093/bib/bbw139 PMC6054162 28077403

[139] West J.A., Davis C.P., Sunwoo H., Simon M.D., Sadreyev R.I., Wang P.I., Tolstorukov M.Y., Kingston R.E. The long noncoding RNAs NEAT1 and MALAT1 bind active chromatin sites. Mol. Cell. 2014; 55:791–802. 25155612 10.1016/j.molcel.2014.07.012 PMC4428586

[140] Li Q., Price J.P., Byers S.A., Cheng D., Peng J., Price D.H. Analysis of the large inactive P-TEFb complex indicates that it contains one 7SK molecule, a dimer of HEXIM1 or HEXIM2, and two P-TEFb molecules containing Cdk9 phosphorylated at threonine 186. J. Biol. Chem. 2005; 280:28819–28826. 15965233 10.1074/jbc.M502712200

[141] Morchikh M., Cribier A., Raffel R., Schwartz O., Morchikh M., Cribier A., Raffel R., Amraoui S., Cau J., Severac D. et al. HEXIM1 and NEAT1 long non-coding RNA form a multi-subunit complex that regulates DNA-mediated innate immune response. Mol. Cell. 2017; 67:387–399. 28712728 10.1016/j.molcel.2017.06.020

