# Supplementary material for: A coding and non-coding transcriptomic perspective on the genomics of human metabolic disease
Source: Nucleic Acids Res. 2018 Jul 9;46(15):7772–92. doi: 10.1093/nar/gky570 (PMC6125682; doi:10.1093/nar/gky570)
Supplement: gky570_Supplemental_Files [file gky570_supplemental_files.zip › Supplemental methods and results revised.pdf]

Supplementary methods and results

Table S1

	MP	DRET	S1-2	IDEAL	HERTG	SEARS*	Weigert*
**Sample size (n) (pre & post)**	47	35	42	72	49	53	20
**Platform**	HTA 2.0	U133+2	U133+2	Illumina	U133+2	U133+2	HTA 2.0
**Gender (M/F)**	16/31	21/14	22/20	0/95	29/20	44/9	7/13
**AGE**	39 (20-51)	50 (20-75)	51 (29-66)	26 (19-45)	26.7 (17-62.5)	51±1	46.4±11
**BMI**	31 (27-43)	25.9 (19.4-32.5)	30.25 (26.2-37.6)	31.13 (26.75-40.1)	25 (19.7-34.2)	35.5±0.8	32.5±4.7
**VO_2 MAX**	27.08 (19.1-46.9)	-	28.3 (17.5-39.1)	-	35.6 (19.6-57.0)	-	22.9±5.1
**Fasting Glucose**	4.64 (3.99-5.2)	5.56 (3.4-7.5)	5.2 (4.3-6.8)	4.85 (3.6-7.1)	5.1 (4.36-8.0)	8.78±0.48	5.6±0.5
**Fasting insulin (ELISA)**	47.86 (9.6-165.9)	21.88 (10.5-67.6)	-	-	-	-	-
**Fasting Insulin (RIA)**			46.6 (8.6-241.9)	52 (15-211)	50 (15-157)	147.6±11.5	-
**HOMA2-IR**	1.0 (0.3-3.4)	0.51 (0.23-1.63)	0.88 (0.17-4.5)	0.96 (0.26-3.68)	1.13 (0.28-2.82)	-	8.3±6.4**
**IS**	100.5 (29.7-324.3)	197.2 (61.2-442.6)	113 (22.2-578.9)	103.9 (27.2-378.2)	105.9 (35.5-354.8)	-	-

Table S1. Pre-intervention demographics of the new and previously published gene-chip data from clinical cohorts (133–142). subject to life-style intervention to improve metabolic health and fitness. Values are median(range). VO_2 MAX is a measure of peak oxygen consumption or aerobic capacity and the units are $ml.min^{-1}.kg^{-1}$ Fasting glucose is $m.mol.l^{-1}$, Fasting insulin units are $pmol.l^{-1}$ while BMI and HOMA2-IR are unit-less and IS is percentile insulin sensitivity from the HOMA2-IR model(143). It is a trait that is highly inherited but also somewhere influenced by behavior (levels of physical activity). *mean and SD from published article. **Matsuda index rather than HOMA2-IR; individual fasting insulin data not available (Weigert) or consistent with modern assays (HERITAGE), and thus values could not be used for individual response analysis.

Table S2: The CORE-IS genes with established links to insulin biology or diabetes and the PubMed citations

Gen ID	Present Study	Pre-existing insulin and diabetes related biochemistry and physiology
AASS	> better S%	**Amino-acids**; aminoadipate-semialdehyde synthase, Lysine catabolism, correlated with IR in pregnancy(1)
ABCG1	> better S%	**Metabolism**; ATP binding cassette subfamily G member 1, Cholesterol efflux protein(2) and GWAS for Insulin/T2DMM(3)
ABHD2	< better S%	**Metabolism**; abhydrolase domain containing 2, hydrolase for triglyceride, acetate, butyrate and palmitate (4)
ACADL	> better S%	**Metabolism**; acyl-CoA dehydrogenase, long chain fatty-acid oxidation, metabolic flexibility(5)
ACOT11	> better S%	**Metabolism**; acyl-CoA thioesterase 11 (Them1) hydrolysis of long chain fatty acyl-CoA thioesters; acts as a 'break' to endogenous lipid supply to mitochondria(6, 7)
ACSS1	> better S%	**Metabolism**; Acyl-CoA synthetase short-chain family member 1, histone acetylation; mito isoform oxidation(8, 9)
AGL	< better S%	**Metabolism**; amylo-alpha-1, 6-glucosidase, 4-alpha-glucanotransferase, glycogen debrancher and loss leads to excessive storage(10)
ALDH1L1	> better S%	**Amino-acids;** aldehyde dehydrogenase 1 family member L1; converts 10-formyltetrahydrofolate to tetrahydrofolate – cofactor for AA metabolism; rs1107366 at ALDH1L1 impacts on glycine-to-serine & GWAS for Insulin/Diabetes (3, 11)
ALDH6A1	> better S%	**Amino-acids**; aldehyde dehydrogenase 6 family member A1 codes for MMSDH (Methylmalonate semialdehyde dehydrogenase, coverts Valine to Propionyl-CoA), down in T2DMM adipose(12)
ATP1A1	< better S%	**Ions**; ATPase Na+/K+ transporting subunit alpha 1, part of the insulin secretion pathway & down-regulated in diabetic cardiomyopathy(13)
B4GALT6	< better S%	**Signalling**; beta-1,4-galactosyltransferase 6, produces lactosyl-ceramide (LacCer), ganglioside GM3 precursor which inhibits insulin receptor. LacCer accumulates during development of impaired fasting glucose(14, 15)
BDH1	> better S%	**Metabolism**; 3-hydroxybutyrate dehydrogenase 1, regulated by hypoxia(16)
BRD8	> better S%	**Transcription**; bromodomain containing 8 known as SMAP/p120, a E1A-binding protein p400 subunit regulating PPARγ target-genes during adipogenesis. 9-cis-RA dependent coactivator of PPARγ/RXR(17)
CALM1	< better S%	**Signalling**; calmodulin 1, transcription of leads to activation of Akt in a Ca2+- and insulin-independent manner(18–20)
CARNS1	< better S%	**Amino-acids**; carnosine synthase 1, carnosine considered 'protective'; converts beta-alanine to carnosine(21, 22)
CD38	< better S%	**Signalling**; CD38 molecule, multifunctional with ADP-ribosyl cyclase activity; glucagon signalling & insulin secretion(23, 24)
CDK14	< better S%	**Signalling**; cyclin dependent kinase 14, Regulates LDL receptor LRP6 via wnt ligand, (LRP6 was also negatively related to S%) and LRP6 related to muscle insulin sensitivity(25, 26).
CDK2AP1	< better S%	**Cell-cycle**; cyclin dependent kinase 2 associated protein 1, inhibits CDK2 activity; loss of CDK2 leads to beta-cell dysfunction via FOXO1(27), GWAS for Insulin/T2DMM (3, 28, 29)
CEBPD	> better S%	**Transcription**; CCAAT/enhancer-binding protein delta; linked to inflammation and adipogenesis, loss enhances pro-apoptotic BIM in pancreas beta-cell(30, 31), linked to KLF15 (32–35)
CERS6	< better S%	**Signalling**; ceramide synthase 6, generates C14- and C16-ceramides; Obesity-induced CerS6-dependent C16:0 ceramide production promotes weight gain and glucose intolerance(36)
CORO1C	< better S%	**Signalling**; coronin 1C, actin dependent processes, rac1 relocation (glucose uptake)(37, 38)
CRBN	> better S%	**Metabolism**; cereblon, endogenous substrate of glutamine synthetase (GS), negative regulator of AMPK(39, 40)

CTH	> better S%	**Signalling**; cystathionine γ lyase (CSE) produces hydrogen sulfide (H2S); promotes adipogenesis & targets pyruvate carboxylase, increasing HGO; insulin signalling(41, 42)
DDR1	> better S%	**Signalling**; discoidin domain receptor tyrosine kinase 1, collagen receptor tyrosine-kinase that regulates IGFR trafficking(43), GWAS for Insulin/T2DMM(3, 28, 29)
DHRS7	< better S%	**Metabolism**; dehydrogenase-reductase 7, relative of 11β-hydroxysteroid dehydrogenase 1, a drug target for T2DMM(44)
DHTKD1	> better S%	**Amino-acids**; dehydrogenase E1 and transketolase domain containing 1. Regulator of 2-aminoadipate, impacting on glucose regulation. mitochondrial protein L-lysine-degradation pathway, 2-oxoadipate substrate for production of superoxide(1); GWAS for T2DMM(3, 28, 29)
ERBB3	< better S%	**Signalling**; erb-b2 receptor tyrosine kinase 3, a heregulin receptor (NRG1a), regulates hepatic glucose output. ERBB3 decreases nuclear FoxO1 via activation of AKT and ERK pathways in liver(45, 46), GWAS for Insulin/T2DMM(3)
FRMD3	< better S%	**Signalling**; FERM domain containing 3, membrane protein with genetic links to blood pressure and diabetes (47, 48)
G0S2	< better S%	**Metabolism**; G0/G1 switch 2, inhibitor of lipase and down-regulated in T2DM(49, 50)
GLUL	> better S%	**Amino-acids**; glutamate-ammonia ligase, forms glutamine and increased by exercise and starvation via FOXO1, GWAS for Insulin/T2DMM(3)
GPAT3	> better S%	**Metabolism**; glycerol-3-phosphate acyltransferase 3, synthesis of glycerol-lipids, glucocorticoid and insulin regulated (51)
GPD1	< better S%	**Metabolism**; glycerol-3 phosphate dehydrog 1 Cytosolic redox regulator linked to 11β-HSD1 activity, decreased after weight-loss and over-expresion associated with IR(44, 52)
GRB14	< better S%	**Signalling**; growth factor receptor bound protein 14, Ca2+ -dependent negative regulator of insulin receptor. Reduced by weight-loss (28, 52, 53); GWAS for Insulin/T2DMM (3, 28, 29)
HBP1	> better S%	**Transcription**; HMG-box transcription factor 1, regulated in vitro by high glucose and H_2O_2, downstream effector of the PI3K/Akt (54)
HEXIM1	< better S%	**Signalling**; hexamethylene bisacetamide inducible 1, binds lncRNA NEAT1, influences transcription elongation; Leptin signalling in hypothalamus and muscle repair linked to glucocorticoid receptor (55, 56)
HOMER1	< better S%	**Signalling**; homer scaffolding protein 1, neuromuscular, >in T2DMM model(57, 58)
IGF1R	> better S%	**Signalling**; insulin like growth factor 1 receptor, marker of beta-cell ageing, induced by IR and insulin-binding(59, 60), GWAS for Insulin/T2DMM(3)
INSR	> better S%	**Signalling**; Insulin receptor(61), GWAS for Insulin/T2DMM(3)
ITIH5	< better S%	**Signalling**; inter-alpha-trypsin inhibitor heavy chain family member 5, Both DNA methylation and expression correlated with BMI, adipokine down-regulated in adipose after weight-loss (62, 63)
KCNN3	< better S%	**Signalling**; potassium calcium-activated channel subfamily N member 3 (SK3), islet signalling/insulin secretion (64)
KLF15	> better S%	**Transcription**; Kruppel like factor 15, A circadian nitrogen homeostasis gene, liver glucose and amino acid metabolism. IR regulated in muscle and fat and adipogenesis (via C/EBPd)(32–35), GWAS for Insulin/T2DMM(3)
KLHL31	< better S%	**Signalling**; kelch like family member 31, wnt regulated myogenesis, GWAS for Insulin/T2DMM(3, 28, 29)
LDHA	< better S%	**Metabolism**; lactate dehydrogenase A, deficiency leads to glycogen disease XI; isoform favours pyruvate conversion to lactate (65)
LDHB	> better S%	**Metabolism**; lactate dehydrogenase B, "cardiac form" favours oxidation to pyruvate (NADH), T2DMM regulated (66)
LGALS1	< better S%	**Cell-cycle**; galectin 1, cell proliferation, CD69 ligand, increased in T2DM adipose & by over-feeding (67, 68)
LGR5	> better S%	**Signalling**; leucine rich repeat containing G protein-coupled receptor (GPR49), co-receptor for Wnt signalling (69), down-regulated in severe IR(70), GWAS for Insulin/T2DMM(3, 28, 29)
LPL	> better S%	**Metabolism**; LPL, triglyceride, regulator of Insulin signalling (5, 71, 72), GWAS for Insulin/T2DMM(3)

MAPKAP1	< better S%	**Signalling**; mitogen-activated protein kinase associated protein 1, (MIP1/SIN1), phosphorylation suppresses TORC2 kinase activity independent of IRS1 preventing insulin induced Akt phosphorylation by mTORC2(73)
MCCC1	> better S%	**Amino-acids**; methylcrotonoyl-CoA carboxylase1 mito leucine catabolism; BMI cis-eQTL, antiviral for RNA viruses(74, 75)
ME2	< better S%	**Metabolism**; malic enzyme 2, mito NAD-dependent conversion of malate to pyruvate, in response to elevated amino acids(76)
MSTN	< better S%	**Signalling**; Myostatin, loss linked to improved S% and MSTN receptor (acvr2b) positively correlated with S%(77)
NDUFAF5	< better S%	**Mitochondria**; NADH:ubiquinone oxidoreductase complex assembly factor 5 (C20orf7) adenosylmethionine-depen' methyltransferase introducing hydroxyl group into Complex I (NDUFS7), 'anti-diabetic drug-target '(78, 79)
NSF	< better S%	**Signalling**; N-ethylmaleimide sensitive factor, vesicle fusing ATPase promoting secretion of apolipoproteins and TAG (hepatocyte); Influences insulin secretion and GLUT trafficking(80, 81)
NUCB2	< better S%	**Signalling**; nucleobindin 2 produces 'Nesfatin-1'; CNS role in energy balance and central influence over liver IR(82, 83), GWAS for Insulin/T2DMM (3, 28, 29)
PARK2	< better S%	**Metabolism**; Parkin, E3 ubiquitin ligase, altered in beta-cells by T2DM; Inhibition reduces glucose-stimulated insulin secretion *in vitro*(84)
PCYT2	> better S%	**Metabolism**; phosphate cytidylyltransferase 2 ethanolamine (ET) Consumes CTP, and phosphoethanolamine, CTP linked to IR(85, 86)
PDHX	< better S%	**Amino-acids /Metabolism**; pyruvate dehydrogenase complex component X, binds to the 'E3' of PDH, alpha-ketoglutarate dehydrogenase complex (KGDC), and branched-chain alpha-keto acid dehydrogenase complex (BCKDC)(35, 87)
PDLIM7	< better S%	**Signalling**; PDZ and LIM domain 7, PI3K linked, In muscle Nedd4-1 (E3 ubiquitin ligase) and GLUT4 linked ('enigma')(88, 89)
PGK1	< better S%	**Metabolism**; phosphoglycerate kinase 1, Glycolysis and kinase at PDHK1 (T338) inhibiting PDH. Inhibited by HDAC3 (K220) & insulin promotes K220 deacetylation(90, 91)
PGM2	< better S%	**Metabolism**; phosphoglucomutase-2, converts glucose 1-phosphate (from glycogen) to glucose 6-phosphate and vice versa(92)
PHKB	< better S%	**Metabolism**; glycogen phosphorylase kinase β-subunit (same in muscle/hepatic); activates glycogen phosphorylase to release glucose-1-phosphate from glycogen; exercise regulated
PKM	< better S%	**Metabolism**; pyruvate kinase (muscle), glycolytic ATP yielding; PK M2 to M1 switch linked to Warburg effect; marker of successful fat-loss (93, 94)
PON2	< better S%	**Metabolism**; paraoxonase 2, hydrolyze LDL, Inhibited by valproic acid (HDAC inhibitor, anti-diabetic activity); lactonase activity(95, 96), GWAS for Insulin/T2DMM(3)
PPM1L	< better S%	**Metabolism**; protein phosphatase Mg2+/Mn2+ dependent 1L, ER transmembrane protein, ceramide transport, genetic link obesity (97, 98)
PPP1R3B	< better S%	**Metabolism**; protein phosphatase 1 regulatory subunit 3B, liver/muscle regulator of glycogen synthesis; hepatic steatosis risk gene(99, 100), GWAS for Insulin/T2DMM(3)
PPP2R5C	< better S%	**Signalling**; protein phosphatase 2 regulatory subunit B'gamma, regulatory subunit of PP2A. PP2A inhibits AKT and glucose consumption. Inactivation of PPP2R5C in hepatocytes increases glucose uptake, de novo lipogenesis and insulin sensitivity(101)
PRDX6	> better S%	**Signalling**; peroxiredoxin 6, redox, short chain fatty acid & phospholipid related, down-reg in gestational diabetes (66, 102)
PRKAG3	< better S%	**Signalling**; protein kinase AMP-activated non-catalytic subunit gamma 3; gain of function increases glycogen storage, protein down-regulated by ET, up-reg in severe IR(70, 103)
RAPH1	< better S%	**Signalling**; Ras association (RalGDS/AF-6) and pleckstrin homology domains 1, a lamellipodin protein. PI(3,4)P2 directs a distinct branch of the PI3K pathway and insulin sensitivity & RAPH1 binds PI(3,4)P2(104). Genetic variant linked to Serum butyrylcholinesterase(105), GWAS for Insulin/T2DMM(3)
RDH10	< better S%	**Metabolism**; retinol dehydrogenase 10, reversible first step of retinoic acid formation; inhibited by insulin in liver cells; pancreas organogenesis(106, 107)
RND3	< better S%	**Signalling**; Rho family GTPase 3, but devoid of GTP hydrolytic activity; RND3 physically interacts with Snail1 protein (transcription), enhancing Snail1 ubiquitination(108), and GWAS for Insulin/T2DMM(3)(28, 29)

RNF10	< better S%	**Cell-cycle**; ring finger protein 10, regulated by retinoic acid, genomic variant linked to obesity in Pima Indians(109, 110), GWAS for Insulin/T2DMM(3)
ROBO1	< better S%	**Signalling**; roundabout guidance receptor 1, age-dependent relationship with BMI; receptor found in pancreas beta/alpha cells(111–113)
RXRG	< better S%	**Signalling**; retinoid X receptor gamma, fed-fast regulated nuclear receptor, Vitamin-A sensitive, genetic association with lipids (114, 115), GWAS for Insulin/T2DMM(28, 29)
SEC61A1	< better S%	**Signalling**; Sec61 translocon alpha 1 subunit, SEC61 ER channel complex; point mutation leads to loss of beta-cell function, sensitive to palmitate lipotoxicity (116)
SEPT11	< better S%	**Signalling**; septin 11, a cytoskeletal GTPase, up-regulated by obesity and IR, Interacts with caveolin-1 and FABP5 while loss impairs insulin signalling in adipocytes(117)
SESN3	< better S%	**Signalling**; sestrin 3, inhibits mTORC1, activates mTORC2: Up-reg in T2DMM, over-expression improves IS in liver (118, 119)
SETDB2	< better S%	**Epigenetic**; SET domain bifurcated 2, is a histone H3 methyltransferase, glucocorticoid induced, regulates insig2a expression; insulin-mediated decrease in Insig2a promotes FA synthesis(120)
SFRP4	< better S%	**Signalling**; secreted frizzled related protein 4, Inhibitor of WNT signalling, linked to islet inflammation and defective insulin secretion; protein elevated in T2DMM; impairs first-phase of glucose-stimulated insulin secretion(121, 122)
SGCG	> better S%	**Signalling**; sarcoglycan gamma, binds dystrophin-associated glycoprotein complex, GWAS for Insulin/T2DMM(3, 28, 29, 123)
SGMS2	< better S%	**Signalling**; sphingomyelin synthase 2, Loss enhances Insulin action in liver, adipose and muscle in vivo, while *in vitro* loss impairs insulin(124, 125)
SLC16A10	< better S%	**Amino-acids**; TAT1, solute carrier family 16 member 10. aromatic amino acid transport (tryptophan, tyrosine, phenylalanine); Up-regulated in extreme IR(70, 126)
SLC27A1	> better S%	**Metabolism**; solute carrier family 27a1 (FATP1), long chain fatty acid transporter. mTORC1-S6K1 promotes translocation to membrane. Deletion protective against IR, while 'activator' (Telmisartan) improves insulin sensitivity increasing FATP1(127, 128)
SLC43A1	> better S%	**Amino-acids;** solute carrier family 43 member 1 (LAT3) sodium dependent leucine uptake; down-regulated in IR(70, 129)
SPARC	< better S%	**Signalling**; secreted protein acidic and cysteine rich, secreted by adipocytes and pancreatic stellate cells; increased in gestational diabetes mellitus, reduces survival of beta-cells(60, 130)
SSX2IP	< better S%	**Cell-cycle**; SSX family member 2 interacting protein. a centriolar satellite protein with genetic association with adiposity (72, 131), GWAS for Insulin/T2DMM(3)
WWOX	< better S%	**DNA-repair**; WW domain containing oxidoreductase fragile site-encoded Wwox protein, GWAS for Insulin/T2DMM(3, 132)

Table S2. A systematic PUBMED search was carried out using the gene symbol, and the terms 'Insulin' 'Diabetes' or 'Obesity'. If >20 articles were found with Gene Symbol + Insulin, the articles were examined and evidence for a clear role in the biology of insulin resistance in humans was extracted. If there were fewer than 20 articles, additional search terms were utilized until either no link was found or a credible link was located. In this sense, the 87 connected genes represent a large sub-set of the exhaustive matrix of gene ID vs all combinations of relevant research terms. Location at a GWAS loci was not considered sufficient evidence on its own.

Table S3. Gender comparison phenotype data

	male	female
HOMA2_IR	0.10	0.11
Age	43.49	42.39
Log_insulin	1.76	1.77
Fasting_G	5.10	4.97
G_2hr_pre1	7.10	7.44
BMI	30.94	31.68
vo2max* (ml/kg/min)	30.54	24.52
*fat-mass higher in females (n=89) vs males (n=89) and so calculated vo2max was 20% lower. Estimated vo2max per kg lean mass was similar.		

Figure S1

Probe-set expression: affected vs unaffected by customisation

Technical replication (RNA profiled 3-months apart)

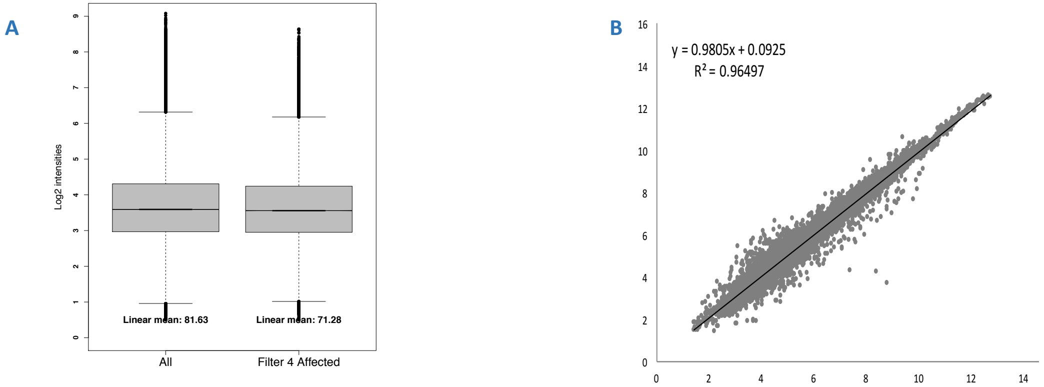

Figure S1. Box-plots of *final* probe-set expression values for those filtered versus those that have had low-expressed probes removed and reproducibility of detection. S1A. Single genomic hit probes were scanned for expression in muscle, and probe signals below a value of 10 units AND a coefficient greater than 25% (i.e. low signal, high variance) were removed. Such probes <u>*would*</u> have ended up in a probe-set that had a mean signal of >70 units (S2A) illustrating that these 'low & variable' probes are inconsistent with the remaining members of the probe-set. **S1B**. Six RNA samples were run 3 months apart and probe-sets were plotted against each other, time-1 versus time-2 (2,700 probe-sets from the main results). R^2=0.96

Figure S2

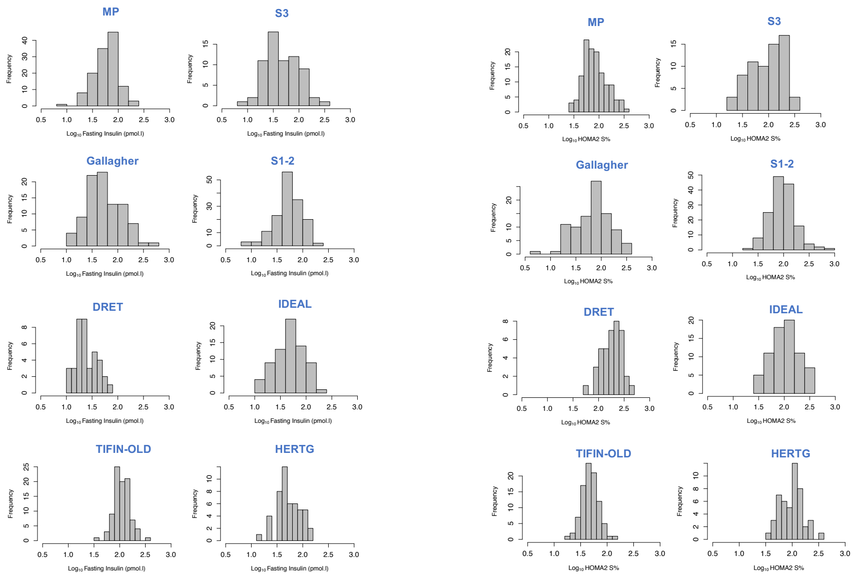

Figure S2. Distribution of Log_{10} (pre-intervention) fasting insulin and HOMA2 calculated insulin sensitivity (Log_{10} IS) values across all cohorts.

Figure S3

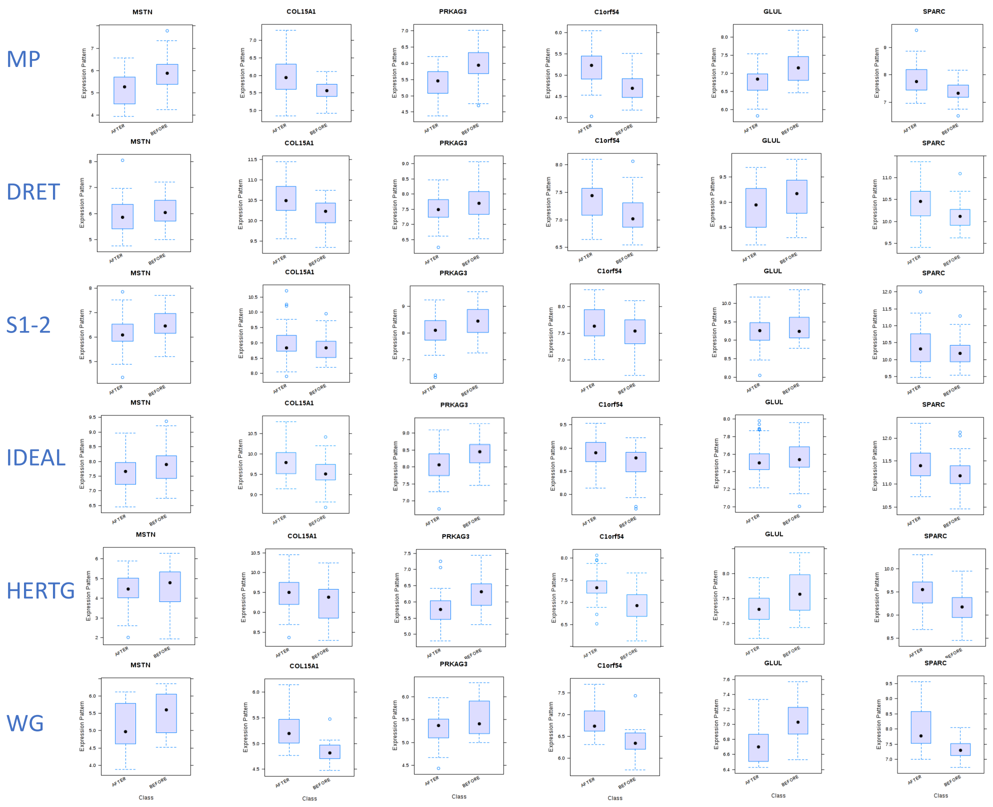

Figure S3. Examples of treatment responsive CORE-IS genes across diverse clinical interventions. Six clinical interventions were carried out and muscle samples obtained before and after the intervention, in the fasted state. MP was a 6-week intervention that utilized a time-efficient low volume high-intensity cycle exercise protocol (3 x 15min exercise per week); DRET represents a high volume 16-week resistance exercise training program; STRRIDE I and II represent 40-week high volume mixed mode exercise training, including aerobic exercise, walking and resistance training. IDEAL as a 16-week calorie restriction and high-volume resistance training program. HERTG was a 20-week aerobic training program using cycles while WGT was a high volume 8 week endurance training cycle program. The duration of the interventions varied ~ 7 fold, the total volume of training (time spent exercising) varied ~ 6.5 fold while the gene-expression responses were very consistent. Data plots created in www.networkanalyst.ca.

Figure S4

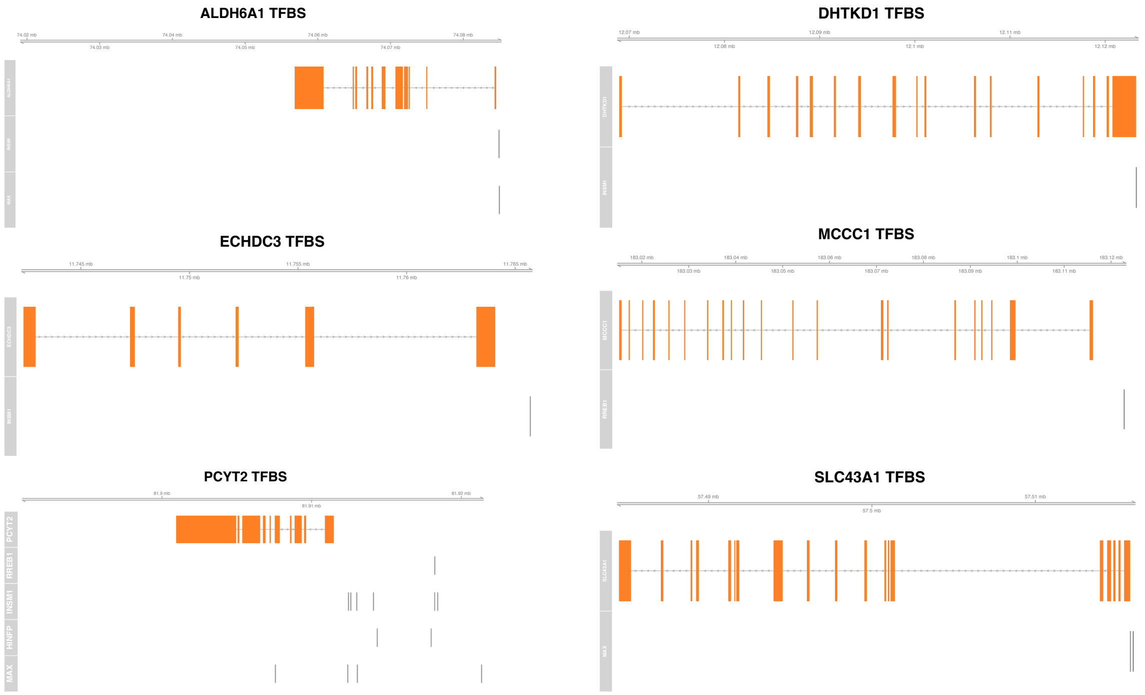

Figure S4. Conserved transcription factor (TF) binding-site analysis up and down-stream of the two gene-sets (149), contrasting with TF enriched in all Core-IS genes and all exercise-regulated Core-IS genes (Figure 2a), identified 4 TF binding sites unique to each list and not enriched in the entire set of exercise-responsive CORE-IS genes. Multi-tissue 'guilt-by-association' analysis (151) identified that the nine positive S% genes and distinct TF's (n=4), were themselves co-expressed with 52 genes (threshold of $p<1x10^{-6}$) that was 77-times enriched (over the genome rate; DAVID) for **branched-chain** amino acid catabolic genes (p<1x10-5); in contrast, the 52-gene list contained a further five candidate genes for genetic association with type 2 diabetes (HNF4A, HMGCS2, KHK, IDH2 and RREB1, (28)) over and above the GWAS loci within the CORE-IS list. TF model for 16 core genes (http://opossum.cisreg.ca/oPOSSUM3/ -Anchored Combination Site Analysis (aCSA)

Figure S5

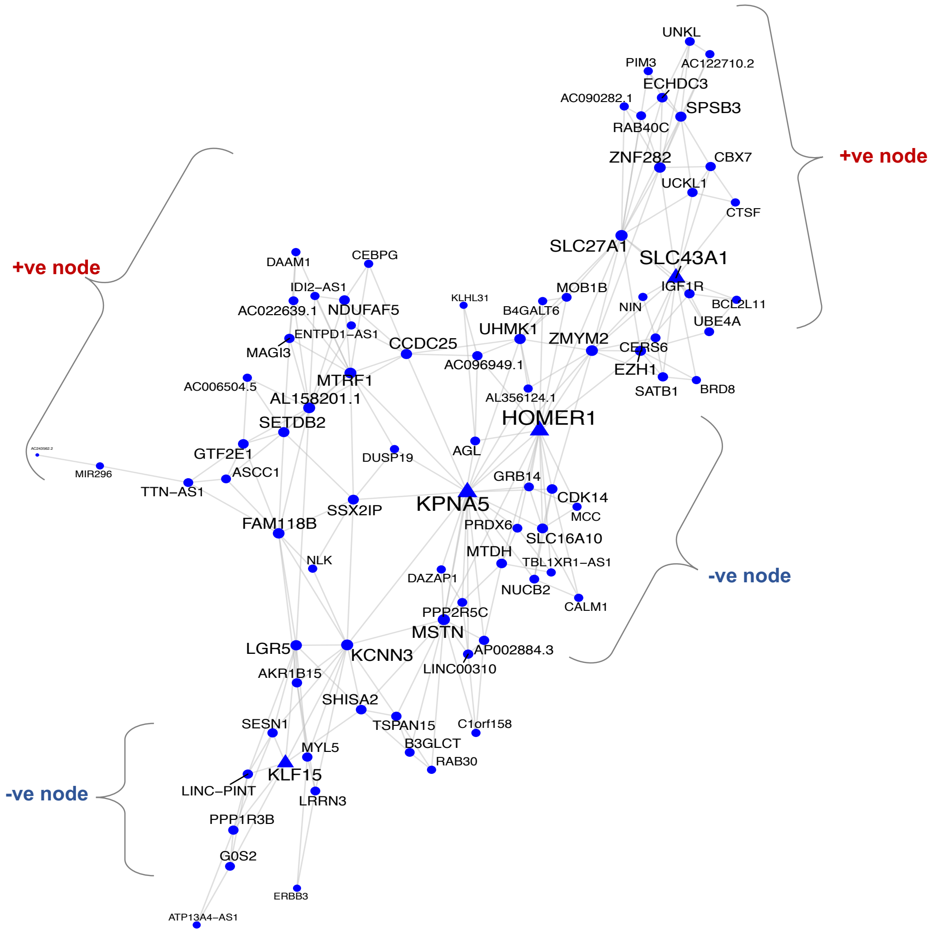

Figure S5. A metabolic gene network responsive to life-style but not drug-treatment. Causal probabilistic networks are utilized to establish a causal relationship between molecules and disease (5). We utilized the dynamic response to diverse forms of treatment aimed at reversing insulin resistance, along with measured fasting relationships to estimate if planar filtered networks (72), based on data from 191 individuals, was enriched in genes dynamically regulated, harboring causal genetic variant related to metabolic disease, or dominated by proven T2DMM disease pathways. MEGENA (72) was used to identify discrete planar filtered networks using the CORE-IS gene list as input (FDR<1% and spearman gene correlation; p<0.01 for module significance and p<0.01 for network connectivity). Protein-Protein interaction analysis (74) facilitated the interpretation of the identified networks while providing additional biological plausibility to each module (73). Node and label size are proportional to the node degree value within each distinct module. This was the third ranked discrete planar filtered network (83 genes, 20% ncRNA, 66% negatively co-varying with *in vivo* fasting IS). The network contained four genes (GOS2, AGL, LAT3 and ECHDC3) regulated *in vivo* in proportion to changes in fasting IS, and contained numerous genes with established links to insulin signaling, metabolic regulation and metabolic disease (e.g. IGF1R, GOS2, AGL, KLF15). Interestingly, the majority of this module was not, on average, responsive to anti-diabetes drug treatment (PPAR[illegible] agonist), given to insulin resistant subjects for three months (51). The protein-protein interaction analysis characterized 33 protein-coding members of the input list forming a network defined as 'positive regulation of cellular metabolic processes and signaling' (FDR<0.02%) as well as including a number of insulin- and lipid-related signaling

molecules (B4GALT6, CDK14, CERS6, FATP1, NLK, IGF1R and SESN1) (93–100) including GRB14**,** one of the most reproducible 'metabolic disease' risk (101–103). GRB14 (Chr2, receptor tyrosine kinase adaptor protein,(137)) is most closely co-expressed with four additional genes, from distinct chromosomes, that are also negatively correlated with IS (KPNA5 (nuclear importin protein; (161) HOMER1 (scaffolding protein; (162) MCC (candidate tumor suppressor; (163) and SLC16A10 (TAT1, is aromatic amino acid transporter) while co-expressed with PRDX6 (antioxidant protein (164)) which was positively associated with IS.

Supplemental references from literature review

1. Wu,Y., Williams,E.G., Dubuis,S., Mottis,A., Jovaisaite,V., Houten,S.M., Argmann,C.A., Faridi,P., Wolski,W., Kutalik,Z., *et al.* (2014) Multilayered genetic and omics dissection of mitochondrial activity in a mouse reference population. *Cell*, **158**, 1415–30.
2. Dayeh,T., Tuomi,T., Almgren,P., Perfilyev,A., Jansson,P.-A., de Mello,V.D., Pihlajamäki,J., Vaag,A., Groop,L., Nilsson,E., *et al.* (2016) DNA methylation of loci within *ABCG1* and *PHOSPHO1* in blood DNA is associated with future type 2 diabetes risk. *Epigenetics*, **11**, 482–488.
3. EBI (2017) GWAS Catalog 2017. *https://www.ebi.ac.uk/gwas/home*.
4. M.,N.K., V.B.S.C.,T., G.K.,V., B.,C.S., Guntupalli,S. and J.S.,B. (2016) Molecular characterization of human ABHD2 as TAG lipase and ester hydrolase. *Biosci. Rep.*, **36**, e00358–e00358.
5. Jans,A., Sparks,L.M., van Hees,A.M.J., Gjelstad,I.M.F., Tierney,A.C., Risérus,U., Drevon,C. a, Roche,H.M., Schrauwen,P. and Blaak,E.E. (2011) Transcriptional Metabolic Inflexibility in Skeletal Muscle Among Individuals With Increasing Insulin Resistance. *Obesity (Silver Spring).*, **19**, 2158–2166.
6. Han,S. and Cohen,D.E. (2012) Functional characterization of thioesterase superfamily member 1/Acyl-CoA thioesterase 11: implications for metabolic regulation. *J. Lipid Res.*, **53**, 2620–31.
7. Zhang,Y., Li,Y., Niepel,M.W., Kawano,Y., Han,S., Liu,S., Marsili,A., Larsen,P.R., Lee,C.-H.C.-H. and Cohen,D.E. (2012) Targeted deletion of thioesterase superfamily member 1 promotes energy expenditure and protects against obesity and insulin resistance. *Proc Natl Acad Sci U S A*, **109**, 5417–22.
8. Gao,X., Lin,S.-H., Ren,F., Li,J.-T., Chen,J.J.-J., Yao,C.-B., Yang,H.-B., Jiang,S.-X., Yan,G.-Q., Wang,D., *et al.* (2016) Acetate functions as an epigenetic metabolite to promote lipid synthesis under hypoxia. *Nat. Commun.*, **7**, 11960.
9. Castro,L.F.C., Lopes-Marques,M., Wilson,J.M., Rocha,E., Reis-Henriques,M.A., Santos,M.M. and Cunha,I. (2012) A novel Acetyl-CoA synthetase short-chain subfamily member 1 (Acss1) gene indicates a dynamic history of paralogue retention and loss in vertebrates. *Gene*, **497**, 249–255.
10. Ko,J.S., Moon,J.S., Seo,J.K., Yang,H.R., Chang,J.Y. and Park,S.S. (2014) A mutation analysis of the AGL gene in Korean patients with glycogen storage disease type III. *J. Hum. Genet.*, **59**, 42–45.
11. Xie,W., Wood,A.R., Lyssenko,V., Weedon,M.N., Knowles,J.W., Alkayyali,S., Assimes,T.L., Quertermous,T., Abbasi,F., Paananen,J., *et al.* (2013) Genetic variants associated with glycine metabolism and their role in insulin sensitivity and type 2 diabetes. *Diabetes*, **62**, 2141–50.
12. Dharuri,H., 't Hoen,P.A.C., van Klinken,J.B., Henneman,P., Laros,J.F.J., Lips,M.A., el Bouazzaoui,F., van Ommen,G.-J.B., Janssen,I., van Ramshorst,B., *et al.* (2014) Downregulation of the acetyl-CoA metabolic network in adipose tissue of obese diabetic individuals and recovery after weight loss. *Diabetologia*, **57**, 2384–2392.
13. Saini,C., Petrenko,V., Pulimeno,P., Giovannoni,L., Berney,T., Hebrok,M., Howald,C., Dermitzakis,E.T. and Dibner,C. (2016) A functional circadian clock is required for proper insulin secretion by human pancreatic islet cells. *Diabetes, Obes. Metab.*, **18**, 355–365.
14. Bozic,J., Markotic,A., Cikes-Culic,V., Novak,A., Borovac,J.A., Vucemilovic,H., Trgo,G. and Ticinovic Kurir,T. (2017) Ganglioside GM3 content in skeletal muscles is increased in type 2 but decreased in type 1 diabetes rat models: implications of glycosphingolipid metabolism in pathophysiology of diabetes. *J. Diabetes*, 10.1111/1753-0407.12569.
15. Mayo,L., Trauger,S.A., Blain,M., Nadeau,M., Patel,B., Alvarez,J.I., Mascanfroni,I.D., Yeste,A., Kivisäkk,P., Kallas,K., *et al.* (2014) Regulation of astrocyte activation by glycolipids drives chronic CNS inflammation. *Nat. Med.*, **20**, 1147–1156.
16. Maurer,G.D., Brucker,D.P., Bähr,O., Harter,P.N., Hattingen,E., Walenta,S., Mueller-Klieser,W., Steinbach,J.P. and Rieger,J. (2011) Differential utilization of ketone bodies by neurons and glioma cell lines: a rationale for ketogenic diet as experimental glioma therapy. *BMC Cancer*, **11**, 315.
17. Couture,J.-P., Nolet,G., Beaulieu,E., Blouin,R. and Gévry,N. (2012) The p400/Brd8 Chromatin

Remodeling Complex Promotes Adipogenesis by Incorporating Histone Variant H2A.Z at PPARγ Target Genes. *Endocrinology*, **153**, 5796–5808.
18. Chen,Z., Ding,L., Yang,W., Wang,J., Chen,L., Chang,Y., Geng,B., Cui,Q., Guan,Y. and Yang,J. (2017) Hepatic Activation of the FAM3C-HSF1-CaM Pathway Attenuates Hyperglycemia of Obese Diabetic Mice. *Diabetes*, **66**, 1185–1197.
19. Chen,M., Zhang,J. and Manley,J.L. (2010) Turning on a fuel switch of cancer: hnRNP proteins regulate alternative splicing of pyruvate kinase mRNA. *Cancer Res.*, **70**, 8977–8980.
20. Márquez-Quiñones,A., Mutch,D.M., Debard,C., Wang,P., Combes,M., Roussel,B., Holst,C., Martinez,J.A., Handjieva-Darlenska,T., Kalouskova,P., *et al.* (2010) Adipose tissue transcriptome reflects variations between subjects with continued weight loss and subjects regaining weight 6 mo after caloric restriction independent of energy intake. *Am. J. Clin. Nutr.*, **92**, 975–984.
21. Stegen,S., Stegen,B., Aldini,G., Altomare,A., Cannizzaro,L., Orioli,M., Gerlo,S., Deldicque,L., Ramaekers,M., Hespel,P., *et al.* (2015) Plasma carnosine, but not muscle carnosine, attenuates high-fat diet-induced metabolic stress. *Appl. Physiol. Nutr. Metab.*, **40**, 868–876.
22. Albrecht,T., Schilperoort,M., Zhang,S., Braun,J.D., Qiu,J., Rodriguez,A., Pastene,D.O., Krämer,B.K., Köppel,H., Baelde,H., *et al.* (2017) Carnosine Attenuates the Development of both Type 2 Diabetes and Diabetic Nephropathy in BTBR ob/ob Mice. *Sci. Rep.*, **7**, 44492.
23. Rah,S.-Y. and Kim,U.-H. (2015) CD38-mediated Ca2+ signaling contributes to glucagon-induced hepatic gluconeogenesis. *Sci. Rep.*, **5**, 10741.
24. Ota,H., Tamaki,S., Itaya-Hironaka,A., Yamauchi,A., Sakuramoto-Tsuchida,S., Morioka,T., Takasawa,S. and Kimura,H. (2012) Attenuation of glucose-induced insulin secretion by intermittent hypoxia via down-regulation of CD38. *Life Sci.*, **90**, 206–211.
25. Karczewska-Kupczewska,M., Stefanowicz,M., Matulewicz,N., Nikołajuk,A. and Strączkowski,M. (2016) Wnt Signaling Genes in Adipose Tissue and Skeletal Muscle of Humans With Different Degrees of Insulin Sensitivity. *J. Clin. Endocrinol. Metab.*, **101**, 3079–3087.
26. Li,S., Song,W., Jiang,M., Zeng,L., Zhu,X. and Chen,J. (2014) Phosphorylation of cyclin Y by CDK14 induces its ubiquitination and degradation. *FEBS Lett.*, **588**, 1989–1996.
27. Kim,S.Y., Lee,J.-H., Merrins,M.J., Gavrilova,O., Bisteau,X., Kaldis,P., Satin,L.S. and Rane,S.G. (2017) Loss of Cyclin-dependent Kinase 2 in the Pancreas Links Primary β-Cell Dysfunction to Progressive Depletion of β-Cell Mass and Diabetes. *J. Biol. Chem.*, **292**, 3841–3853.
28. Fuchsberger,C., Flannick,J., Teslovich,T.M., Mahajan,A., Agarwala,V., Gaulton,K.J., Ma,C., Fontanillas,P., Moutsianas,L., McCarthy,D.J., *et al.* (2016) The genetic architecture of type 2 diabetes. *Nature*, **536**, 41–7.
29. Shungin,D., Winkler,T.W., Croteau-Chonka,D.C., Ferreira,T., Locke,A.E., Mägi,R., Strawbridge,R.J., Pers,T.H., Fischer,K., Justice,A.E., *et al.* (2015) New genetic loci link adipose and insulin biology to body fat distribution. *Nature*, **518**, 187–196.
30. Hogan,N.T., Whalen,M.B., Stolze,L.K., Hadeli,N.K., Lam,M.T., Springstead,J.R., Glass,C.K. and Romanoski,C.E. (2017) Transcriptional networks specifying homeostatic and inflammatory programs of gene expression in human aortic endothelial cells. *Elife*, **6**.
31. Lim,G.E., Albrecht,T., Piske,M., Sarai,K., Lee,J.T.C., Ramshaw,H.S., Sinha,S., Guthridge,M.A., Acker-Palmer,A., Lopez,A.F., *et al.* (2015) 14-3-3ζ coordinates adipogenesis of visceral fat. *Nat. Commun.*, **6**, 7671.
32. Mori,T., Sakaue,H., Iguchi,H., Gomi,H., Okada,Y., Takashima,Y., Nakamura,K., Nakamura,T., Yamauchi,T., Kubota,N., *et al.* (2005) Role of Kruppel-like Factor 15 (KLF15) in Transcriptional Regulation of Adipogenesis. *J. Biol. Chem.*, **280**, 12867–12875.
33. Takashima,M., Ogawa,W., Hayashi,K., Inoue,H., Kinoshita,S., Okamoto,Y., Sakaue,H., Wataoka,Y., Emi,A., Senga,Y., *et al.* (2010) Role of KLF15 in regulation of hepatic gluconeogenesis and metformin action. *Diabetes*, **59**, 1608–15.
34. Elbein,S.C., Kern,P.A., Rasouli,N., Yao-Borengasser,A., Sharma,N.K. and Das,S.K. (2011) Global gene expression profiles of subcutaneous adipose and muscle from glucose-tolerant, insulin-sensitive, and insulin-resistant individuals matched for BMI. *Diabetes*, **60**, 1019–1029.

35. Lynch,C.J. and Adams,S.H. (2014) Branched-chain amino acids in metabolic signalling and insulin resistance. *Nat. Rev. Endocrinol.*, **10**, 723–736.
36. Turpin,S.M.M., Nicholls,H.T.T., Willmes,D.M.M., Mourier,A., Brodesser,S., Wunderlich,C.M.M., Mauer,J., Xu,E., Hammerschmidt,P., Brönneke,H.S.S., *et al.* (2014) Obesity-induced CerS6-dependent C16:0 ceramide production promotes weight gain and glucose intolerance. *Cell Metab.*, **20**, 678–686.
37. Williamson,R.C., Cowell,C.A.M., Hammond,C.L., Bergen,D.J.M., Roper,J.A., Feng,Y., Rendall,T.C.S., Race,P.R. and Bass,M.D. (2014) Coronin-1C and RCC2 guide mesenchymal migration by trafficking Rac1 and controlling GEF exposure. *J. Cell Sci.*, **127**, 4292–4307.
38. Nozaki,S., Takeda,T., Kitaura,T., Takenaka,N., Kataoka,T. and Satoh,T. (2013) Akt2 regulates Rac1 activity in the insulin-dependent signaling pathway leading to GLUT4 translocation to the plasma membrane in skeletal muscle cells. *Cell. Signal.*, **25**, 1361–71.
39. Lee,K.M., Yang,S.-J., Choi,J.-H. and Park,C.-S. (2014) Functional Effects of a Pathogenic Mutation in Cereblon (CRBN) on the Regulation of Protein Synthesis via the AMPK-mTOR Cascade. *J. Biol. Chem.*, **289**, 23343–23352.
40. Van Nguyen,T., Lee,J.E., Sweredoski,M.J., Yang,S.-J., Jeon,S.-J., Harrison,J.S., Yim,J.-H., Lee,S.G., Handa,H., Kuhlman,B., *et al.* (2016) Glutamine Triggers Acetylation-Dependent Degradation of Glutamine Synthetase via the Thalidomide Receptor Cereblon. *Mol. Cell*, **61**, 809–820.
41. Ju,Y., Untereiner,A., Wu,L. and Yang,G. (2015) H2S-induced S-sulfhydration of pyruvate carboxylase contributes to gluconeogenesis in liver cells. *Biochim. Biophys. Acta - Gen. Subj.*, **1850**, 2293–2303.
42. Cai,J., Shi,X., Wang,H., Fan,J., Feng,Y., Lin,X., Yang,J., Cui,Q., Tang,C., Xu,G., *et al.* (2016) Cystathionine γ lyase–hydrogen sulfide increases peroxisome proliferator-activated receptor γ activity by sulfhydration at C139 site thereby promoting glucose uptake and lipid storage in adipocytes. *Biochim. Biophys. Acta - Mol. Cell Biol. Lipids*, **1861**, 419–429.
43. Malaguarnera,R., Nicolosi,M.L., Sacco,A., Morcavallo,A., Vella,V., Voci,C., Spatuzza,M., Xu,S.-Q., Iozzo,R. V., Vigneri,R., *et al.* (2015) Novel cross talk between IGF-IR and DDR1 regulates IGF-IR trafficking, signaling and biological responses. *Oncotarget*, **6**, 16084–16105.
44. Stambergova,H., Skarydova,L., Dunford,J.E. and Wsol,V. (2014) Biochemical properties of human dehydrogenase/reductase (SDR family) member 7. *Chem. Biol. Interact.*, **207**, 52–57.
45. Arai,T., Ono,Y., Arimura,Y., Sayama,K., Suzuki,T., Shinjo,S., Kanai,M., Abe,S., Semba,K. and Goda,N. (2017) Type I neuregulin1α is a novel local mediator to suppress hepatic gluconeogenesis in mice. *Sci. Rep.*, **7**, 42959.
46. Caillaud,K., Boisseau,N., Ennequin,G., Chavanelle,V., Etienne,M., Li,X., Denis,P., Dardevet,D., Lacampagne,A. and Sirvent,P. (2016) Neuregulin 1 improves glucose tolerance in adult and old rats. *Diabetes Metab.*, **42**, 96–104.
47. Martini,S., Nair,V., Patel,S.R., Eichinger,F., Nelson,R.G., Weil,E.J., Pezzolesi,M.G., Krolewski,A.S., Randolph,A., Keller,B.J., *et al.* (2013) From Single Nucleotide Polymorphism to Transcriptional Mechanism: A Model for FRMD3 in Diabetic Nephropathy. *Diabetes*, **62**, 2605–2612.
48. Liang,J., Le,T.H., Edwards,D.R.V., Tayo,B.O., Gaulton,K.J., Smith,J.A., Lu,Y., Jensen,R.A., Chen,G., Yanek,L.R., *et al.* (2017) Single-trait and multi-trait genome-wide association analyses identify novel loci for blood pressure in African-ancestry populations. *PLOS Genet.*, **13**, e1006728.
49. Laurens,C., Badin,P.-M., Louche,K., Mairal,A., Tavernier,G., Marette,A., Tremblay,A., Weisnagel,S.J., Joanisse,D.R., Langin,D., *et al.* (2016) G0/G1 Switch Gene 2 controls adipose triglyceride lipase activity and lipid metabolism in skeletal muscle. *Mol. Metab.*, **5**, 1–11.
50. Ma,T., Dong,J.P., Sekula,D.J., Fei,D.L., Lamph,W.W., Henderson,M., Lu,Y., Blumen,S., Freemantle,S.J. and Dmitrovsky,E. (2013) Repression of exogenous gene expression by the retinoic acid target gene G0S2. *Int. J. Oncol.*, **42**, 1743–1753.
51. Shan,D., Li,J. -l., Wu,L., Li,D., Hurov,J., Tobin,J.F., Gimeno,R.E. and Cao,J. (2010) GPAT3 and GPAT4 are regulated by insulin-stimulated phosphorylation and play distinct roles in adipogenesis. *J. Lipid Res.*, **51**, 1971–1981.

52. Park,J.-J., Berggren,J.R., Hulver,M.W., Houmard,J.A. and Hoffman,E.P. (2006) GRB14, GPD1, and GDF8 as potential network collaborators in weight loss-induced improvements in insulin action in human skeletal muscle. *Physiol. Genomics*, **27**, 114–121.
53. Desbuquois,B., Carré,N. and Burnol,A.-F.F. (2013) Regulation of insulin and type 1 insulin-like growth factor signaling and action by the Grb10/14 and SH2B1/B2 adaptor proteins. *FEBS J.*, **280**, 794–816.
54. Wang,S., Cao,Z., Xue,J., Li,H., Jiang,W., Cheng,Y., Li,G. and Zhang,X. (2017) A positive feedback loop between Pim-1 kinase and HBP1 transcription factor contributes to hydrogen peroxide-induced premature senescence and apoptosis. *J. Biol. Chem.*, **292**, 8207–8222.
55. Dhar-Mascareno,M., Ramirez,S.N., Rozenberg,I., Rouille,Y., Kral,J.G. and Mascareno,E.J. (2016) Hexim1, a Novel Regulator of Leptin Function, Modulates Obesity and Glucose Disposal. *Mol. Endocrinol.*, **30**, 314–324.
56. Hong,P., Chen,K., Huang,B., Liu,M., Cui,M., Rozenberg,I., Chaqour,B., Pan,X., Barton,E.R., Jiang,X.-C., *et al.* (2012) HEXIM1 controls satellite cell expansion after injury to regulate skeletal muscle regeneration. *J. Clin. Invest.*, **122**, 3873–3887.
57. Lu,J., Gan,J., Fu,G., Ding,L. and Zheng,Q. (2015) The Impact of Small RNA Interference Against Homer1 on Rats with Type 2 Diabetes and ERK Phosphorylation. *Cell Biochem. Biophys.*, **73**, 597–601.
58. Feng,W., Tu,J., Yang,T., Vernon,P.S., Allen,P.D., Worley,P.F. and Pessah,I.N. (2002) Homer Regulates Gain of Ryanodine Receptor Type 1 Channel Complex. *J. Biol. Chem.*, **277**, 44722–44730.
59. Federici,M., Lauro,D., D'Adamo,M., Giovannone,B., Porzio,O., Mellozzi,M., Tamburrano,G., Sbraccia,P. and Sesti,G. (1998) Expression of insulin/IGF-I hybrid receptors is increased in skeletal muscle of patients with chronic primary hyperinsulinemia. *Diabetes*, **47**, 87–92.
60. Ryall,C.L., Viloria,K., Lhaf,F., Walker,A.J., King,A., Jones,P., Mackintosh,D., McNeice,R., Kocher,H., Flodstrom-Tullberg,M., *et al.* (2014) Novel Role for Matricellular Proteins in the Regulation of Islet β Cell Survival. *J. Biol. Chem.*, **289**, 30614–30624.
61. Sell,S.M., Reese,D. and Ossowski,V.M. (1994) Insulin-inducible Changes in Insulin Receptor mRNA Splice Variants *. *J. Biol. Chem.*
62. Anveden,Å., Sjöholm,K., Jacobson,P., Palsdottir,V., Walley,A.J., Froguel,P., Al-Daghri,N., McTernan,P.G., Mejhert,N., Arner,P., *et al.* (2012) ITIH-5 Expression in Human Adipose Tissue Is Increased in Obesity. *Obesity*, **20**, 708–714.
63. Ronn,T., Volkov,P., Gillberg,L., Kokosar,M., Perfilyev,A., Jacobsen,A.L., Jorgensen,S.W., Brons,C., Jansson,P.-A., Eriksson,K.-F., *et al.* (2015) Impact of age, BMI and HbA1c levels on the genome-wide DNA methylation and mRNA expression patterns in human adipose tissue and identification of epigenetic biomarkers in blood. *Hum. Mol. Genet.*, **24**, 3792–813.
64. Zhang,M., Houamed,K., Kupershmidt,S., Roden,D. and Satin,L.S. (2005) Pharmacological Properties and Functional Role of K_{slow} Current in Mouse Pancreatic β-Cells. *J. Gen. Physiol.*, **126**, 353–363.
65. Brighenti,E., Carnicelli,D., Brigotti,M. and Fiume,L. (2017) The inhibition of lactate dehydrogenase A hinders the transcription of histone 2B gene independently from the block of aerobic glycolysis. *Biochem. Biophys. Res. Commun.*, **485**, 742–745.
66. Oliva,K., Barker,G., Rice,G.E., Bailey,M.J. and Lappas,M. (2013) 2D-DIGE to identify proteins associated with gestational diabetes in omental adipose tissue. *J. Endocrinol.*, **218**, 165–178.
67. Liu,X., Feng,Q., Chen,Y., Zuo,J., Gupta,N., Chang,Y. and Fang,F. (2009) Proteomics-Based Identification of Differentially-Expressed Proteins Including Galectin-1 in the Blood Plasma of Type 2 Diabetic Patients. *J. Proteome Res.*, **8**, 1255–1262.
68. Cibrián,D. and Sánchez-Madrid,F. (2017) CD69: from activation marker to metabolic gatekeeper. *Eur. J. Immunol.*, 10.1002/eji.201646837.
69. Yan,K.S., Janda,C.Y., Chang,J., Zheng,G.X.Y., Larkin,K.A., Luca,V.C., Chia,L.A., Mah,A.T., Han,A., Terry,J.M., *et al.* (2017) Non-equivalence of Wnt and R-spondin ligands during Lgr5+ intestinal

stem-cell self-renewal. *Nature*, **545**, 238–242.
70. Møller,A.B., Kampmann,U., Hedegaard,J., Thorsen,K., Nordentoft,I., Vendelbo,M.H., Møller,N. and Jessen,N. (2017) Altered gene expression and repressed markers of autophagy in skeletal muscle of insulin resistant patients with type 2 diabetes. *Sci. Rep.*, **7**, 43775.
71. Walton,R.G., Zhu,B., Unal,R., Spencer,M., Sunkara,M., Morris,A.J., Charnigo,R., Katz,W.S., Daugherty,A., Howatt,D.A., *et al.* (2015) Increasing Adipocyte Lipoprotein Lipase Improves Glucose Metabolism in High Fat Diet-induced Obesity. *J. Biol. Chem.*, **290**, 11547–11556.
72. Ng,M.C.Y., Graff,M., Lu,Y., Justice,A.E., Mudgal,P., Liu,C.-T., Young,K., Yanek,L.R., Feitosa,M.F., Wojczynski,M.K., *et al.* (2017) Discovery and fine-mapping of adiposity loci using high density imputation of genome-wide association studies in individuals of African ancestry: African Ancestry Anthropometry Genetics Consortium. *PLOS Genet.*, **13**, e1006719.
73. Liu,P., Gan,W., Inuzuka,H., Lazorchak,A.S., Gao,D., Arojo,O., Liu,D., Wan,L., Zhai,B., Yu,Y., *et al.* (2013) Sin1 phosphorylation impairs mTORC2 complex integrity and inhibits downstream Akt signalling to suppress tumorigenesis. *Nat. Cell Biol.*, **15**, 1340–1350.
74. Sajuthi,S.P., Sharma,N.K., Chou,J.W., Palmer,N.D., McWilliams,D.R., Beal,J., Comeau,M.E., Ma,L., Calles-Escandon,J., Demons,J., *et al.* (2016) Mapping adipose and muscle tissue expression quantitative trait loci in African Americans to identify genes for type 2 diabetes and obesity. *Hum. Genet.*, **135**, 869–880.
75. Cao,Z., Xia,Z., Zhou,Y., Yang,X., Hao,H., Peng,N., Liu,S. and Zhu,Y. (2016) Methylcrotonoyl-CoA carboxylase 1 potentiates RLR-induced NF-κB signaling by targeting MAVS complex. *Sci. Rep.*, **6**, 33557.
76. Pongratz,R.L., Kibbey,R.G., Shulman,G.I. and Cline,G.W. (2007) Cytosolic and mitochondrial malic enzyme isoforms differentially control insulin secretion. *J. Biol. Chem.*, **282**, 200–7.
77. KLIMENTIDIS,Y.C., BEA,J.W., THOMPSON,P., KLIMECKI,W.T., HU,C., WU,G., NICHOLAS,J.S., RYCKMAN,K.K. and CHEN,Z. (2016) Genetic Variant in ACVR2B Is Associated with Lean Mass. *Med. Sci. Sport. Exerc.*, **48**, 1270–1275.
78. Rhein,V.F., Carroll,J., Ding,S., Fearnley,I.M. and Walker,J.E. (2016) NDUFAF5 Hydroxylates NDUFS7 at an Early Stage in the Assembly of Human Complex I. *J. Biol. Chem.*, **291**, 14851–14860.
79. Zurita Rendon,O. and Shoubridge,E.A. (2012) Early complex I assembly defects result in rapid turnover of the ND1 subunit. *Hum. Mol. Genet.*, **21**, 3815–3824.
80. Ramalingam,L., Yoder,S.M., Oh,E. and Thurmond,D.C. (2014) Munc18c: A controversial regulator of peripheral insulin action. *Trends Endocrinol. Metab.*, **25**, 601–608.
81. Xiong,Q., Yu,C., Zhang,Y., Ling,L., Wang,L. and Gao,J. (2017) Key proteins involved in insulin vesicle exocytosis and secretion (Review). *Biomed. Reports*, **6**, 134–139.
82. Dore,R., Levata,L., Lehnert,H. and Schulz,C. (2017) Nesfatin-1: functions and physiology of a novel regulatory peptide. *J. Endocrinol.*, **232**, R45–R65.
83. Santoso,P., Nakata,M., Shiizaki,K., Boyang,Z., Parmila,K., Otgon-Uul,Z., Hashimoto,K., Satoh,T., Mori,M., Kuro-o,M., *et al.* (2017) Fibroblast growth factor 21, assisted by elevated glucose, activates paraventricular nucleus NUCB2/Nesfatin-1 neurons to produce satiety under fed states. *Sci. Rep.*, **7**, 45819.
84. Volkov,P., Bacos,K., Ofori,J.K., Esguerra,J.L.S., Eliasson,L., Rönn,T. and Ling,C. (2017) Whole-Genome bisulfite sequencing of human pancreatic islets reveals novel differentially methylated regions in type 2 diabetes pathogenesis. *Diabetes*, **66**, 1074–1085.
85. Taylor,A., Schenkel,L.C., Yokich,M. and Bakovic,M. (2017) Adaptations to excess choline in insulin resistant and *Pcyt2* deficient skeletal muscle. *Biochem. Cell Biol.*, **95**, 223–231.
86. Fullerton,M.D., Hakimuddin,F., Bonen,A. and Bakovic,M. (2009) The Development of a Metabolic Disease Phenotype in CTP:Phosphoethanolamine Cytidylyltransferase-deficient Mice. *J. Biol. Chem.*, **284**, 25704–25713.
87. Vijayakrishnan,S., Callow,P., Nutley,M.A., McGow,D.P., Gilbert,D., Kropholler,P., Cooper,A., Byron,O. and Lindsay,J.G. (2011) Variation in the organization and subunit composition of the

mammalian pyruvate dehydrogenase complex E2/E3BP core assembly. *Biochem. J.*, **437**, 565–574.
88. Pedersen,H.K., Gudmundsdottir,V. and Brunak,S. (2017) Pancreatic Islet Protein Complexes and Their Dysregulation in Type 2 Diabetes. *Front. Genet.*, **8**, 43.
89. Magalhaes,A.C., Dunn,H. and Ferguson,S.S.G. (2012) Regulation of GPCR activity, trafficking and localization by GPCR-interacting proteins. *Br. J. Pharmacol.*, **165**, 1717–1736.
90. Wang,S., Jiang,B., Zhang,T., Liu,L., Wang,Y., Wang,Y., Chen,X., Lin,H., Zhou,L., Xia,Y., *et al.* (2015) Insulin and mTOR Pathway Regulate HDAC3-Mediated Deacetylation and Activation of PGK1. *PLOS Biol.*, **13**, e1002243.
91. Li,X., Jiang,Y., Meisenhelder,J., Yang,W., Hawke,D.H., Zheng,Y., Xia,Y., Aldape,K., He,J., Hunter,T., *et al.* (2016) Mitochondria-Translocated PGK1 Functions as a Protein Kinase to Coordinate Glycolysis and the TCA Cycle in Tumorigenesis. *Mol. Cell*, **61**, 705–719.
92. Kelley,D.E., Goodpaster,B.H. and Storlien,L. (2002) Muscle triglyceride and insulin resistance. *Annu Rev Nutr*, **22**, 325–346.
93. Nakatsu,D., Horiuchi,Y., Kano,F., Noguchi,Y., Sugawara,T., Takamoto,I., Kubota,N., Kadowaki,T. and Murata,M. (2015) L-cysteine reversibly inhibits glucose-induced biphasic insulin secretion and ATP production by inactivating PKM2. *Proc Natl Acad Sci U S A*, **112**, E1067-76.
94. Luo W Hu H,C.R.Z.J.K.M.O.R.C.R.N.P.A.S.G.L., Luo,W., Hu,H., Chang,R., Zhong,J., Knabel,M., O'Meally,R., Cole,R.N., Pandey,A. and Semenza,G.L. (2011) Pyruvate kinase M2 is a PHD3-stimulated coactivator for hypoxia-inducible factor 1. *Cell*, **145**, 732–744.
95. Précourt,L.-P., Amre,D., Denis,M.-C., Lavoie,J.-C., Delvin,E., Seidman,E. and Levy,E. (2011) The three-gene paraoxonase family: Physiologic roles, actions and regulation. *Atherosclerosis*, **214**, 20–36.
96. Khan,S., Kumar,S. and Jena,G. (2016) Valproic acid reduces insulin-resistance, fat deposition and FOXO1-mediated gluconeogenesis in type-2 diabetic rat. *Biochimie*, **125**, 42–52.
97. Kusano,R., Fujita,K., Shinoda,Y., Nagaura,Y., Kiyonari,H., Abe,T., Watanabe,T., Matsui,Y., Fukaya,M., Sakagami,H., *et al.* (2016) Targeted disruption of the mouse protein phosphatase *ppm1l* gene leads to structural abnormalities in the brain. *FEBS Lett.*, **590**, 3606–3615.
98. Chen,Y., Zhu,J., Lum,P.Y., Yang,X., Pinto,S., MacNeil,D.J., Zhang,C., Lamb,J., Edwards,S., Sieberts,S.K., *et al.* (2008) Variations in DNA elucidate molecular networks that cause disease. *Nature*, **452**, 429–435.
99. Mehta,M.B., Shewale,S. V., Sequeira,R.N., Millar,J.S., Hand,N.J. and Rader,D.J. (2017) Hepatic protein phosphatase 1 regulatory subunit 3B (Ppp1r3b) promotes hepatic glycogen synthesis and thereby regulates fasting energy homeostasis. *J. Biol. Chem.*, 10.1074/jbc.M116.766329.
100. Raffield,L.M., Louie,T., Sofer,T., Jain,D., Ipp,E., Taylor,K.D., Papanicolaou,G.J., Avilés-Santa,L., Lange,L.A., Laurie,C.C., *et al.* (2017) Genome-wide association study of iron traits and relation to diabetes in the Hispanic Community Health Study/Study of Latinos (HCHS/SOL): potential genomic intersection of iron and glucose regulation? *Hum. Mol. Genet.*, **26**, 1966–1978.
101. Hahn,K., Miranda,M., Francis,V.A., Vendrell,J., Zorzano,A. and Teleman,A.A. (2010) PP2A Regulatory Subunit PP2A-B' Counteracts S6K Phosphorylation. *Cell Metab.*, **11**, 438–444.
102. Pacifici,F., Arriga,R., Sorice,G.P., Capuani,B., Scioli,M.G., Pastore,D., Donadel,G., Bellia,A., Caratelli,S., Coppola,A., *et al.* (2014) Peroxiredoxin 6, a Novel Player in the Pathogenesis of Diabetes. *Diabetes*, **63**, 3210–3220.
103. Costford,S.R., Kavaslar,N., Ahituv,N., Chaudhry,S.N., Schackwitz,W.S., Dent,R., Pennacchio,L.A., McPherson,R. and Harper,M.-E. (2007) Gain-of-Function R225W Mutation in Human AMPKγ3 Causing Increased Glycogen and Decreased Triglyceride in Skeletal Muscle. *PLoS One*, **2**, e903.
104. Li,H. and Marshall,A.J. (2015) Phosphatidylinositol (3,4) bisphosphate-specific phosphatases and effector proteins: A distinct branch of PI3K signaling. *Cell. Signal.*, **27**, 1789–1798.
105. Benyamin,B., Middelberg,R.P., Lind,P.A., Valle,A.M., Gordon,S., Nyholt,D.R., Medland,S.E., Henders,A.K., Heath,A.C., Madden,P.A.F., *et al.* (2011) GWAS of butyrylcholinesterase activity identifies four novel loci, independent effects within BCHE and secondary associations with

metabolic risk factors. *Hum. Mol. Genet.*, **20**, 4504–14.
106. Obrochta,K.M., Krois,C.R., Campos,B. and Napoli,J.L. (2015) Insulin Regulates Retinol Dehydrogenase Expression and All- *trans* -retinoic Acid Biosynthesis through FoxO1. *J. Biol. Chem.*, **290**, 7259–7268.
107. Arregi,I., Climent,M., Iliev,D., Strasser,J., Gouignard,N., Johansson,J.K., Singh,T., Mazur,M., Semb,H., Artner,I., *et al.* (2016) Retinol Dehydrogenase-10 Regulates Pancreas Organogenesis and Endocrine Cell Differentiation via Paracrine Retinoic Acid Signaling. *Endocrinology*, **157**, 4615–4631.
108. Liu,B., Dong,H., Lin,X., Yang,X., Yue,X., Yang,J., Li,Y., Wu,L., Zhu,X., Zhang,S., *et al.* (2016) RND3 promotes Snail 1 protein degradation and inhibits glioblastoma cell migration and invasion. *Oncotarget*, **7**, 82411–82423.
109. Malik,Y.S., Sheikh,M.A., Lai,M., Cao,R. and Zhu,X. (2013) RING finger protein 10 regulates retinoic acid-induced neuronal differentiation and the cell cycle exit of P19 embryonic carcinoma cells. *J. Cell. Biochem.*, **114**, 2007–2015.
110. Huang,K., Nair,A.K., Muller,Y.L., Piaggi,P., Bian,L., del Rosario,M., Knowler,W.C., Kobes,S., Hanson,R.L., Bogardus,C., *et al.* (2014) Whole exome sequencing identifies variation in *CYB5A* and *RNF10* associated with adiposity and type 2 diabetes. *Obesity*, **22**, 984–988.
111. Yang,Y.H.C., Manning Fox,J.E., Zhang,K.L., MacDonald,P.E. and Johnson,J.D. (2013) Intraislet SLIT-ROBO signaling is required for beta-cell survival and potentiates insulin secretion. *Proc. Natl. Acad. Sci.*, **110**, 16480–16485.
112. Lasky-Su,J., Lyon,H.N., Emilsson,V., Heid,I.M., Molony,C., Raby,B.A., Lazarus,R., Klanderman,B., Soto-Quiros,M.E., Avila,L., *et al.* (2008) On the Replication of Genetic Associations: Timing Can Be Everything! *Am. J. Hum. Genet.*, **82**, 849–858.
113. Hwang,D.-Y., Kohl,S., Fan,X., Vivante,A., Chan,S., Dworschak,G.C., Schulz,J., van Eerde,A.M., Hilger,A.C., Gee,H.Y., *et al.* (2015) Mutations of the SLIT2–ROBO2 pathway genes SLIT2 and SRGAP1 confer risk for congenital anomalies of the kidney and urinary tract. *Hum. Genet.*, **134**, 905–916.
114. Chen,Y.L., Pei,D., Hung,Y.J., Lee,C.H., Hsiao,F.C., Wu,C.Z., Lin,J.D., Hsu,C.H., Chang,J.B. and Hsieh,C.H. (2015) Associations between genetic variants and the severity of metabolic syndrome in subjects with type 2 diabetes. *Genet. Mol. Res.*, **14**, 2518–2526.
115. Sentinelli,F., Minicocci,I., Montali,A., Nanni,L., Romeo,S., Incani,M., Cavallo,M.G., Lenzi,A., Arca,M. and Baroni,M.G. (2013) Association of RXR-Gamma Gene Variants with Familial Combined Hyperlipidemia: Genotype and Haplotype Analysis. *J. Lipids*, **2013**, 1–7.
116. Lloyd,D.J., Wheeler,M.C. and Gekakis,N. (2010) A Point Mutation in Sec61 1 Leads to Diabetes and Hepatosteatosis in Mice. *Diabetes*, **59**, 460–470.
117. Moreno-Castellanos,N., Rodríguez,A., Rabanal-Ruiz,Y., Fernández-Vega,A., López-Miranda,J., Vázquez-Martínez,R., Frühbeck,G. and Malagón,M.M. (2017) The cytoskeletal protein septin 11 is associated with human obesity and is involved in adipocyte lipid storage and metabolism. *Diabetologia*, **60**, 324–335.
118. Lee,J.H., Budanov,A.V., Talukdar,S., Park,E.J., Park,H.L.H.-W.H.L., Park,H.L.H.-W.H.L., Bandyopadhyay,G., Li,N., Aghajan,M., Jang,I., *et al.* (2012) Maintenance of Metabolic Homeostasis by Sestrin2 and Sestrin3. *Cell Metab.*, **16**, 311–321.
119. Tao,R., Xiong,X., Liangpunsakul,S. and Dong,X.C. (2015) Sestrin 3 Protein Enhances Hepatic Insulin Sensitivity by Direct Activation of the mTORC2-Akt Signaling. *Diabetes*, **64**, 1211–1223.
120. Roqueta-Rivera,M., Esquejo,R.M., Phelan,P.E., Sandor,K., Daniel,B., Foufelle,F., Ding,J., Li,X., Khorasanizadeh,S. and Osborne,T.F. (2016) SETDB2 Links Glucocorticoid to Lipid Metabolism through Insig2a Regulation. *Cell Metab.*, **24**, 474–484.
121. Brix,J., Krzizek,E., Hoebaus,C., Ludvik,B., Schernthaner,G. and Schernthaner,G. (2016) Secreted Frizzled-Related Protein 4 (SFRP4) is Elevated in Patients with Diabetes Mellitus. *Horm. Metab. Res.*, **48**, 345–348.
122. Liu,F., Qu,H., Li,Y., Tang,Q., Yang,Z., Wang,H. and Deng,H. (2015) Relationship between serum

secreted frizzled-related protein 4 levels and the first-phase of glucose-stimulated insulin secretion in individuals with different glucose tolerance. *Endocr. J.*, **62**, 733–740.

123. Chen,M., Zhang,X., Fang,Q., Wang,T., Li,T. and Qiao,H. (2016) Three single nucleotide polymorphisms associated with type 2 diabetes mellitus in a Chinese population. *Exp. Ther. Med.*, **13**, 121–126.
124. Sugimoto,M., Shimizu,Y., Zhao,S., Ukon,N., Nishijima,K. ichi, Wakabayashi,M., Yoshioka,T., Higashino,K., Numata,Y., Okuda,T., *et al.* (2016) Characterization of the role of sphingomyelin synthase 2 in glucose metabolism in whole-body and peripheral tissues in mice. *Biochim. Biophys. Acta - Mol. Cell Biol. Lipids*, **1861**, 688–702.
125. Park,M., Kaddai,V., Ching,J., Fridianto,K.T., Sieli,R.J., Sugii,S. and Summers,S.A. (2016) A Role for Ceramides, but Not Sphingomyelins, as Antagonists of Insulin Signaling and Mitochondrial Metabolism in C2C12 Myotubes. *J. Biol. Chem.*, **291**, 23978–23988.
126. Halestrap,A.P. (2013) The SLC16 gene family – Structure, role and regulation in health and disease. *Mol. Aspects Med.*, **34**, 337–349.
127. Arif,A., Terenzi,F., Potdar,A.A., Jia,J., Sacks,J., China,A., Halawani,D., Vasu,K., Li,X., Brown,J.M., *et al.* (2017) EPRS is a critical mTORC1–S6K1 effector that influences adiposity in mice. *Nature*, **542**, 357–361.
128. Shiota,A., Shimabukuro,M., Fukuda,D., Soeki,T., Sato,H., Uematsu,E., Hirata,Y., Kurobe,H., Maeda,N., Sakaue,H., *et al.* (2012) Telmisartan ameliorates insulin sensitivity by activating the AMPK/SIRT1 pathway in skeletal muscle of obese db/db mice. *Cardiovasc. Diabetol.*, **11**, 139.
129. Bodoy,S., Fotiadis,D., Stoeger,C., Kanai,Y. and Palacín,M. (2013) The small SLC43 family: Facilitator system l amino acid transporters and the orphan EEG1. *Mol. Aspects Med.*, **34**, 638–645.
130. Kos,K. and Wilding,J.P.H. (2010) SPARC: a key player in the pathologies associated with obesity and diabetes. *Nat. Rev. Endocrinol.*, **6**, 225–235.
131. Klinger,M., Wang,W., Kuhns,S., Barenz,F., Drager-Meurer,S., Pereira,G. and Gruss,O.J. (2014) The novel centriolar satellite protein SSX2IP targets Cep290 to the ciliary transition zone. *Mol. Biol. Cell*, **25**, 495–507.
132. Chang,Y.-C., Chiu,Y.-F., Liu,P.-H., Shih,K.-C., Lin,M.-W., Sheu,W.H.-H., Quertermous,T., Curb,J.D., Hsiung,C.A., Lee,W.-J., *et al.* (2012) Replication of genome-wide association signals of type 2 diabetes in Han Chinese in a prospective cohort. *Clin. Endocrinol. (Oxf).*, **76**, 365–372.
133. Barberio,M.D., Huffman,K.M., Giri,M., Hoffman,E.P., Kraus,W.E. and Hubal,M.J. (2016) Pyruvate Dehydrogenase Phosphatase Regulatory Gene Expression Correlates with Exercise Training Insulin Sensitivity Changes. *Med. Sci. Sports Exerc.*, **48**, 2387–2397.
134. Phillips,B.E., Williams,J.P., Gustafsson,T., Bouchard,C., Rankinen,T., Knudsen,S., Smith,K., Timmons,J.A. and Atherton,P.J. (2013) Molecular Networks of Human Muscle Adaptation to Exercise and Age. *PLoS Genet.*, **9**, e1003389.
135. Sood,S., Gallagher,I.J., Lunnon,K., Rullman,E., Keohane,A., Crossland,H., Phillips,B.E., Cederholm,T., Jensen,T., van Loon,L.J.C., *et al.* (2015) A novel multi-tissue RNA diagnostic of healthy ageing relates to cognitive health status. *Genome Biol.*, **16**, 185.
136. Sood,S., Szkop,K.J., Nakhuda,A., Gallagher,I.J., Murie,C., Brogan,R.J., Kaprio,J., Kainulainen,H., Atherton,P.J., Kujala,U.M., *et al.* (2016) iGEMS: An integrated model for identification of alternative exon usage events. *Nucleic Acids Res.*, **44**, 1–14.
137. Gallagher,I.J., Scheele,C., Keller,P., Nielsen,A.R., Remenyi,J., Fischer,C.P., Roder,K., Babraj,J., Wahlestedt,C., Hutvagner,G., *et al.* (2010) Integration of microRNA changes in vivo identifies novel molecular features of muscle insulin resistance in type 2 diabetes. *Genome Med.*, **2**, 9.
138. Nakhuda,A., Josse,A.R., Gburcik,V., Crossland,H., Raymond,F., Metairon,S., Good,L., Atherton,P.J., Phillips,S.M. and Timmons,J.A. (2016) Biomarkers of browning of white adipose tissue and their regulation. *Am. J. Clin. Nutr.*, 10.3945/ajcn.116.132563.1.
139. Hangelbroek,R.W.J., Fazelzadeh,P., Tieland,M., Boekschoten,M. V, Hooiveld,G.J.E.J., van Duynhoven,J.P.M., Timmons,J.A., Verdijk,L.B., de Groot,L.C.P.G.M., van Loon,L.J.C., *et al.* (2016)

Expression of protocadherin gamma in skeletal muscle tissue is associated with age and muscle weakness. *J. Cachexia. Sarcopenia Muscle*, 10.1002/jcsm.12099.
140. Phillips,B., Kelly,B., Lilja,M., Ponce-González,J., Brogan,R., Morris,D., Gustafsson,T., Kraus,W., Atherton,P., Vollaard,N., *et al.* (2017) A practical and time-efficient high-intensity interval training programme modifies cardio-metabolic risk-factors in adults with risk-factors for Type II diabetes. *Front. Endocrinol. (Lausanne).*, doi: 10.3389/fendo.2017.00229.
141. Sears,D.D., Hsiao,G., Hsiao,A., Yu,J.G., Courtney,C.H., Ofrecio,J.M., Chapman,J. and Subramaniam,S. (2009) Mechanisms of human insulin resistance and thiazolidinedione-mediated insulin sensitization. *Proc. Natl. Acad. Sci. U. S. A.*, **106**, 18745–50.
142. Böhm,A., Hoffmann,C., Irmler,M., Schneeweiss,P., Schnauder,G., Sailer,C., Schmid,V., Hudemann,J., Machann,J., Schick,F., *et al.* (2016) TGF-β contributes to impaired exercise response by suppression of mitochondrial key regulators in skeletal muscle. *Diabetes*, **65**, 2849–2861.
143. Wallace,T.M., Levy,J.C., Matthews,D.R. and Homa,T. (2004) Use and Abuse of HOMA Modeling. *Diabetes Care*, **27**, 1487–1495.
144. Josse,A.R., Atkinson,S.A., Tarnopolsky,M.A. and Phillips,S.M. (2011) Increased Consumption of Dairy Foods and Protein during Diet- and Exercise-Induced Weight Loss Promotes Fat Mass Loss and Lean Mass Gain in Overweight and Obese Premenopausal Women 1 – 4. 10.3945/jn.111.141028.The.
145. Wang,X., Kang,D.D., Shen,K., Song,C., Lu,S., Chang,L.C., Liao,S.G., Huo,Z., Tang,S., Ding,Y., *et al.* (2012) An r package suite for microarray meta-analysis in quality control, differentially expressed gene analysis and pathway enrichment detection. *Bioinformatics*, **28**, 2534–2536.
146. Song,W.M. and Zhang,B. (2015) Multiscale Embedded Gene Co-expression Network Analysis. *PLoS Comput. Biol.*, **11**.
147. McKenzie,A.T., Katsyv,I., Song,W.-M., Wang,M. and Zhang,B. (2016) DGCA: A comprehensive R package for Differential Gene Correlation Analysis. *BMC Syst. Biol.*, **10**, 106.
148. Xia,J., Benner,M.J. and Hancock,R.E.W. (2014) NetworkAnalyst - Integrative approaches for protein-protein interaction network analysis and visual exploration. *Nucleic Acids Res.*, **42**, 167–174.
149. Sui Ho,S.J., Fulton,D.L., Arenillas,D.J., Kwon,A.T. and Wasserman,W.W. (2007) OPOSSUM: Integrated tools for analysis of regulatory motif over-representation. *Nucleic Acids Res.*, **35**, 245–252.
150. van Dam,S., Võsa,U., van der Graaf,A., Franke,L. and de Magalhães,J.P. (2017) Gene co-expression analysis for functional classification and gene–disease predictions. *Brief. Bioinform.*, 10.1093/bib/bbw139.
151. van Dam,S., Craig,T. and de Magalhães,J.P. (2015) GeneFriends: a human RNA-seq-based gene and transcript co-expression database. *Nucleic Acids Res.*, **43**, D1124–D1132.
