# Supplementary figures and images for: A coding and non-coding transcriptomic perspective on the genomics of human metabolic disease
Source: Nucleic Acids Res. 2018 Jul 9;46(15):7772–92. doi: 10.1093/nar/gky570 (PMC6125682; doi:10.1093/nar/gky570)

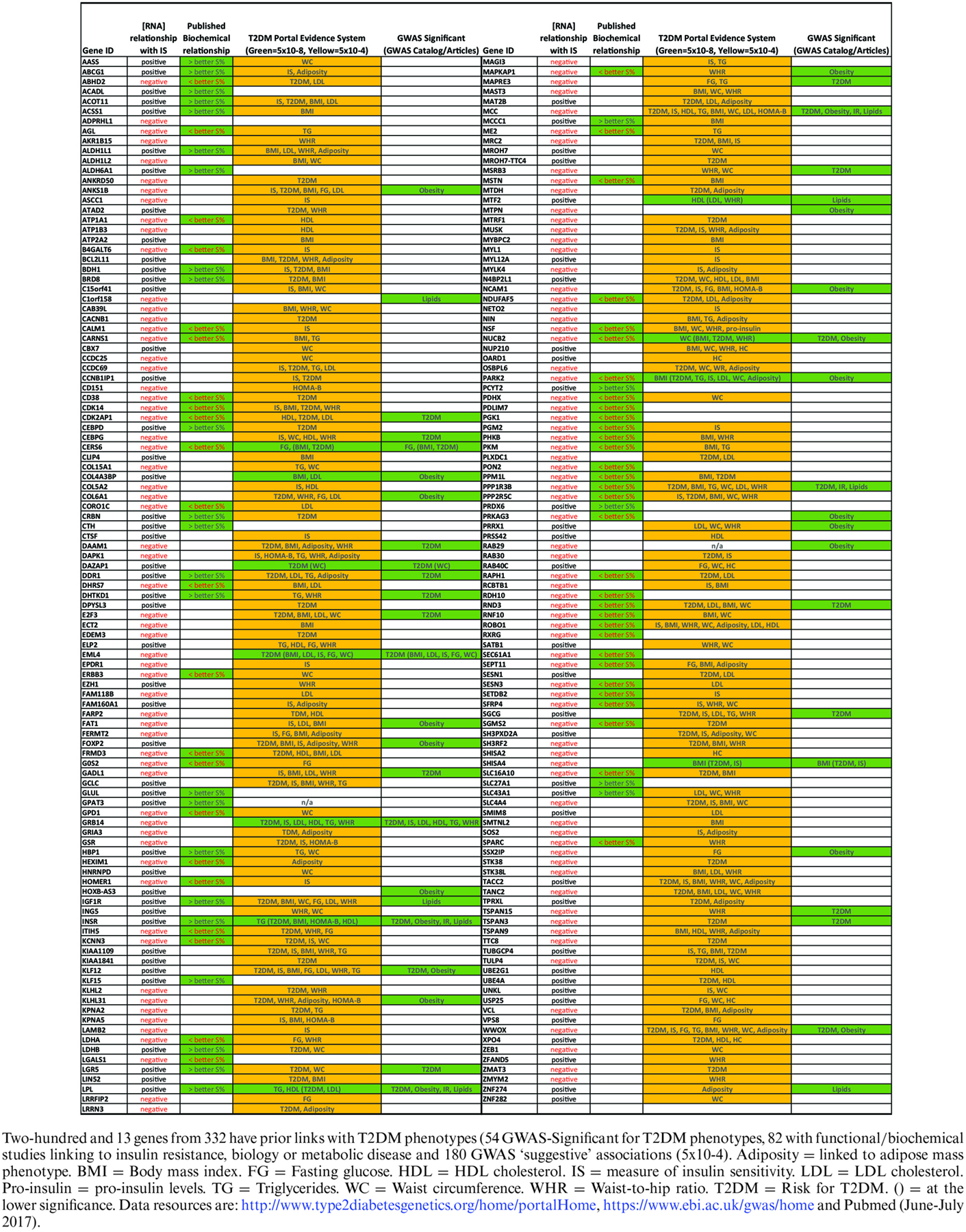

Supplement: gky570_Supplemental_Files [file gky570_supplemental_files.zip › gky570Table 2.jpg]
